# SRF Rearrangements in Soft Tissue Tumors with Muscle Differentiation

**DOI:** 10.3390/biom12111678

**Published:** 2022-11-12

**Authors:** Alice Costa, Livia Gozzellino, Milena Urbini, Valentina Indio, Margherita Nannini, Maria Abbondanza Pantaleo, Silvia Stacchiotti, Annalisa Astolfi, Gianandrea Pasquinelli

**Affiliations:** 1 Department of Experimental, Diagnostic and Specialty Medicine (DIMES), University of Bologna, 40138 Bologna, Italy; 2 Biosciences Laboratory, IRCCS Istituto Romagnolo per lo Studio dei Tumori (IRST) “Dino Amadori”, 47014 Meldola, Italy; 3 Department of Veterinary Medical Sciences, University of Bologna, Ozzano dell’Emilia, 40064 Bologna, Italy; 4 Division of Oncology, IRCCS Azienda Ospedaliero-Universitaria di Bologna, 40138 Bologna, Italy; 5 Department of Medical Oncology, IRCCS Fondazione Istituto Nazionale Tumori (INT), 20133 Milan, Italy; 6 Division of Pathology, IRCCS Azienda Ospedaliero-Universitaria di Bologna, 40138 Bologna, Italy

**Keywords:** myoepithelial neoplasm, SRF, fusion, E2F1, myogenesis

## Abstract

The Serum Response Factor (SRF) is a transcription factor that regulates the expression of a wide set of genes involved in cell proliferation, migration, cytoskeletal organization and myogenesis. Accumulating evidence suggests that *SRF* may play a role in carcinogenesis and tumor progression in various neoplasms, where it is often involved in different fusion events. Here we investigated *SRF* rearrangements in soft tissue tumors, along with a gene expression profile analysis to gain insight into the oncogenic mechanism driven by *SRF* fusion. Whole transcriptome analysis of cell lines transiently overexpressing the SRF::E2F1 chimeric transcript uncovered the specific gene expression profile driven by the aberrant gene fusion, including overexpression of SRF-dependent target genes and of signatures related to myogenic commitment, inflammation and immune activation. This result was confirmed by the analysis of two cases of myoepitheliomas harboring *SRF::E2F1* fusion with respect to *EWSR1*-fusion positive tumors. The recognition of the specific gene signature driven by *SRF* rearrangement in soft tissue tumors could aid the molecular classification of this rare tumor entity and support therapeutic decisions.

## 1. Introduction

Gene fusions, which arise from chromosomal rearrangements, have been largely described in literature as pivotal driver mutations in many types of neoplasia, including benign as well as malignant tumors of hematologic, epithelial and mesenchymal origin [1]. In recent years, gene fusion events have been specifically associated with definite tumor histotypes, leading to their possible role as molecular diagnostic markers. Moreover, some chimeric proteins derived from gene fusions could be direct or indirect therapeutic targets, making the detection of gene fusions ever more relevant [2,3].

Translocation events involving the *SRF* gene have been described in literature for different types of soft tissue tumors. Indeed, *SRF* is often fused to various 3′ partner genes, including *RELA* [4,5,6], *ICA1L* [7], *FOXO* [8] and *STAT6* [9], leading to the production of aberrant transcription factors and thus to the enrichment of *SRF*-involving pathways.

In this paper, we analyze the specific gene expression profile induced by *SRF* gene fusion, focusing on the *SRF::E2F1* chimeric transcript that we previously identified in two cases of myoepithelial neoplasms of the soft tissues [10].

The serum response factor (*SRF*) gene on 6p21.1 encodes a MADS box transcription factor that binds the core sequence of the CArG boxes (CC (A/T) 6 GG) in the promoter of the target genes, leading to the regulation of a wide set of genes, including immediate early genes (as *C-FOS*, JUN and *EGR*), as well as genes involved in cell growth, migration, angiogenesis, cytoskeletal organization, energy metabolism and myogenesis [11,12,13,14]. *SRF* is also highly expressed in skeletal muscle, where it regulates the expression of skeletal muscle-specific genes, as dystrophin, muscle creatine kinase, myoD, and several genes encoding sarcomeric proteins, like α-skeletal actin, myosin light chain, and tropomyosin [15]. Accumulating evidence suggests that *SRF* is involved in the carcinogenesis and tumor progression of various neoplasms, especially in the mesenchymal transition of epithelial cells [16,17].

*E2F1* is a transcription factor belonging to the E2F family, which is implicated in cell cycle regulation and apoptosis [18,19], and displays dual behavior by promoting or inhibiting tumorigenesis, depending on the cellular context [20,21]. To date, fusion events involving *E2F1*, like the *SRF::E2F1* rearrangement investigated in this study, have not been previously reported in other tumors.

## 2. Materials and Methods

### 2.1. Cell Culture and Transfection of SRF::E2F1

HEK293 and HT1080 were obtained from CLS Cell Lines Service (Eppelheim, Germany) and cultured in Dulbecco’s modified Eagle’s medium high glucose (DMEM, Gibco) supplemented with 10% fetal bovine serum (FBS), 1% L-glu and 1% penicillin-streptomycin (complete medium). Plasmid DNA containing the full-length SRF::E2F1 [10] under control of the CMV promoter was used for transfection experiments. Cells were seeded on 6-well plates (9 × 10^5^ cells/well) in DMEM complete medium, and after 24 h, transiently transfected with SRF::E2F1, mock or empty pcDNA3.1 vector using Lipofectamine 2000 (Life Technologies, Delhi, India). Untreated and mock (treated with only Lipofectamine) or pcDNA3.1-transfected cells were used as controls. Total RNA was extracted 48 h after transfection using the *Quick*-RNA^TM^ Miniprep Kit (Zymo Research, Irvine, CA, USA), and reverse transcribed by PrimeScript™ RT reagent Kit with gDNA Eraser (Takara Bio, Shiga, Japan) into cDNA.

### 2.2. RNA-Sequencing

Total RNA (250 ng) extracted from HEK293 and HT1080 transfected cells was used to prepare the RNA libraries with the Illumina Stranded mRNA Prep, Ligation Kit (Illumina, San Diego, CA, USA) following manufacturers’ instructions. Libraries were quantified with the Qubit dsDNA BR Assay Kit (Invitrogen, Carlsbad CA, USA) and sized with Agilent 2100 Bioanalyzer System. Pooled libraries were sequenced at 75 bp in paired end on a NextSeq 500/550 High Output V2 flow cell with an Illumina NextSeq 500 instrument (Illumina).

### 2.3. Bioinformatic Analysis

Paired reads were mapped on the reference human genome hg38 using STAR (https://github.com/alexdobin/STAR accessed on 2 September 2022); duplicates removal, sorting and indexing were performed with Samtools (http://www.htslib.org accessed on 2 September 2022). Gene expression was quantified and normalized as counts per million (CPM) using the python package HTseq-count to obtain the raw gene counts (https://htseq.readthedocs.io/ accessed on 2 September 2022). Subsequently, normalization factors were computed with the R-bioconductor package edgeR (https://bioconductor.org/packages/release/bioc/html/edgeR.html accessed on 2 September 2022). CPM were employed to perform the principal component analysis (PCA) and the evaluation of differential expression (DE). The R package prcomp (https://cran.r-project.org/package=nsprcomp accessed on 2 September 2022) was adopted to perform the PCA. The DE analysis between controls and samples overexpressing the SRF::E2F1 transcript was conducted with the R-bioconductor package edgeR (https://bioconductor.org/packages/release/bioc/html/edgeR.html accessed on 2 September 2022), setting a *p*-value < 0.05. The Gene Set Enrichment Analysis (GSEA) (https://www.gsea-msigdb.org/gsea/index.jsp accessed on 2 September 2022) was performed using the whole expression matrix and selecting the hallmarks, regulatory target (TFT: transcription factor targets), kegg, reactome and oncogenic gene sets. Additionally, parameters were set as follows: “number of permutation” = “1000”; “permutation type” = “gene set”; “enrichment statistic” = “weighted”; “metric for ranking gene” = “log2 ratio of classes” and “normalization mode” = “meandiv”.

Bioinformatics analyses were likewise performed on *SRF::E2F1* fusion-positive tumor samples (L107 and L108) versus *EWSR1*-positive samples (L161 and L162), previously published [10].

### 2.4. Real-Time PCR Analysis

Fusion gene expression and mRNA expression level of *SRF* target genes *(EGR1*, *FOS*, *CALD1*, and *ACGT2)* were evaluated in transfected HEK293 and HT1080 cells by quantitative RT-PCR (CX96 Touch Real-Time-PCR Detection System, Biorad) with Premix Ex Taq™ DNA Polymerase (Takara Bio). Fold changes were evaluated in comparison to untreated samples using the ΔΔCt method. *GAPDH* was used as housekeeping gene. GraphPad PRISM Software was used for statistical analysis. The P value was estimated against pcDNA3.1 by a one-way ANOVA (** *p* < 0.01; *** *p* < 0.001).

## 3. Results

### 3.1. SRF Rearrangement in Myoepithelial Neoplasms

We have previously reported [10] a novel in-frame *SRF::E2F1* fusion in two cases of myoepithelial neoplasms (MN) of the soft tissue: a mixed-type tumor harboring *FUS::KLF17*-rearrangement (case L108) and a spindle cell myoepithelioma with no evidence of other gene fusions (case L107), both lacking pathological evidence of malignancy.

**Figure 1:**
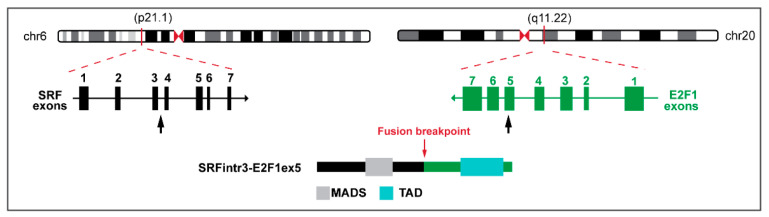
Schematic representation of *SRF::E2F1* fusion transcript and predicted chimeric protein, depicting the exons and protein domains involved. The genomic exon structure of *SRF* and *E2F1* are reported on the left and on the right, respectively. Black arrows indicate the breakpoint. The resulting chimeric protein is shown below [10]. MADS: MAD box DNA binding domain; TAD: Transactivation domain.

In the two *SRF::E2F1* positive patients, the breakpoint was detected in the middle of intron 3 of *SRF* and the middle of exon 5 of *E2F1*, leading to a chimeric protein retaining the MAD box domain of SRF and the TAD domain of E2F1 (Figure 1).

### 3.2. Gene Expression Analysis Driven by SRF Fusion

**Figure 2:**
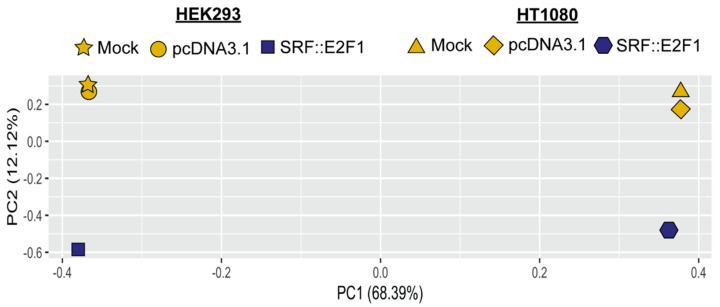
Principal component analysis (PCA) performed on HEK293 and HT1080 cells transfected with SRF::E2F1 fusion transcript analyzed by RNA-sequencing. The unsupervised PCA analysis shows the segregation along the first component between the two different cell lines (HEK293 and HT1080), and, for both cell lines, along the second component between *SRF::E2F1*-overexpressing cells (in blue) and controls (in yellow).

To gain further insight into the oncogenic mechanism driven by *SRF* gene fusion and to uncover its specific gene expression profile, we performed gene expression analysis on *SRF::E2F1*-transfected cell lines. We transiently overexpressed the SRF::E2F1 chimeric transcript into HEK293 and HT1080 recipient cell lines and profiled the whole transcriptome 48 h after transfection. Unsupervised PCA analysis showed a distinct clustering of the samples, with *SRF::E2F1* overexpressing cells separating along the second principal component in both HEK293 and HT1080 with respect to control cells, and displaying a specific gene expression profile driven by *SRF*-fusion de novo expression (Figure 2).

To detect specific genes induced in HEK293 and HT1080 lines transfected with *SRF::E2F1*, we performed a supervised analysis (Supplementary Table S1), identifying 331 differentially expressed genes (*p*-value < 0.05). As expected, *SRF* and *E2F1* were significantly upregulated in *SRF::E2F1*-transfected samples, as well as the immediate early genes *EGR1*, *EGR2*, *EGR* and *EGR4,* with an overexpression of at least seven-fold over control cells. To confirm that this gene expression signature was present and relevant also in *SRF* fusion-positive tumors, we analyzed the gene expression profile of two myoepitheliomas carrying *SRF::E2F1* chimeric fusion with respect to two *EWSR1*-rearranged MN. Supervised analysis on MN tumor samples identified 2,249 differentially expressed genes (*p*-value < 0.05) in *SRF*-fused positive tumors with respect to *EWSR1*-fused positive tumors (Supplementary Table S2). Among the selected genes, *SRF*, *E2F1*, *EGR2*, *EGR3* and *EGR4* were equally upregulated in samples harboring *SRF* rearrangement, with a fold induction of at least four times over *EWSR1*-fused positive tumors.

**Figure 3:**
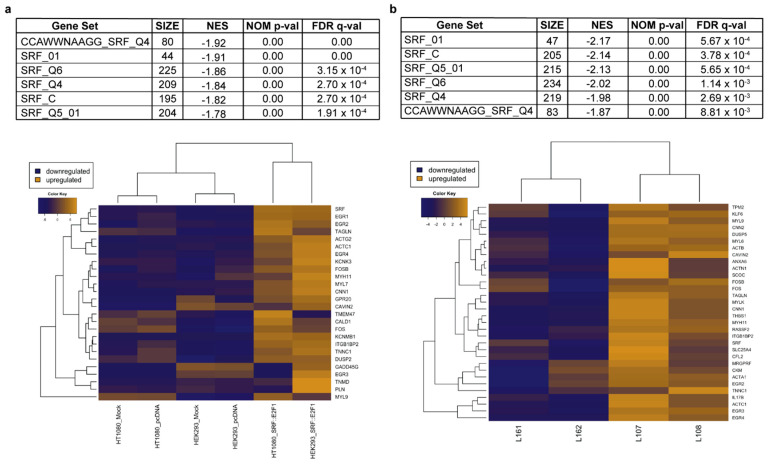
GSEA analyses of transcription factor binding sites’ enrichment in *SRF*-fusion positive samples. Tables and hierarchical clustering showing that SRF binding sites were among the top scoring enriched gene sets in (**a**) *SRF::E2F1* transfected HEK293 and HT1080 cells and (**b**) *SRF*-fused positive tumors. NES: Normalized Enrichment Score; NOM *p*-val: Nominal *p*-value; FDR q-val: False Discovery Rate q-value.

Interestingly, the overriding activation of the *SRF* signaling pathway was confirmed by enrichment analysis of genes harboring specific transcription factor binding sites (GSEA TFT database). Figure 3 shows that in both transfected cell lines and tumor samples, the prevalent transcription-factor-activated signature is the one driven by *SRF*, since all the different SRF-binding motif lists are significantly enriched in *SRF*-fused positive samples versus controls.

**Figure 4:**
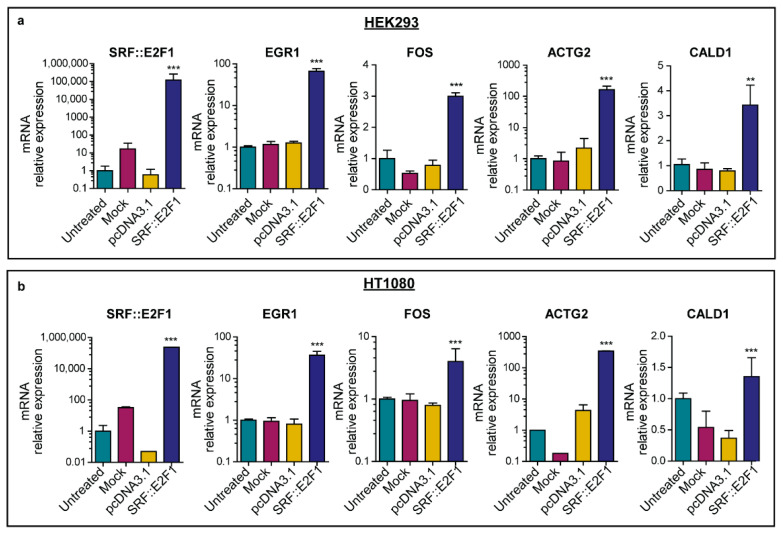
*SRF::E2F1* expression in transfected cell lines. mRNA relative expression of *SRF::E2F1, EGR1, FOS, ACTG2* and *CALD1*, target genes of *SRF*, in HEK293 (**a**) and HT1080 (**b**) cell lines, 48 h after transfection. Fold changes were evaluated in comparison to untreated samples. *GAPDH* was used as housekeeping gene. P value was estimated against pcDNA3.1 by a one-way ANOVA (** *p* < 0.01; *** *p <* 0.001).

Additionally, *FOS* and *FOSB*, two other *SRF* immediate early genes, were among the most enriched genes in both *SRF::E2F1*-transfected cell lines and *SRF* fusion-positive MN. In line with the gene expression results, real time PCR performed on HEK293 and HT1080 samples showed a remarkable increase of *EGR1* and *FOS* mRNA levels in *SRF::E2F1* overexpressing cells with respect to untreated, mock-transfected and empty vector controls (Figure 4), as reported in our previous work for the HEK293 cell line [10].

**Figure 5:**
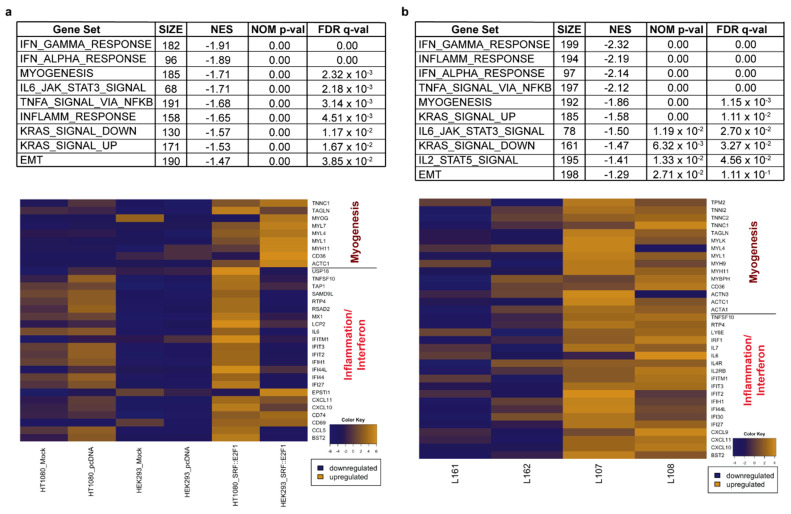
GSEA analyses of hallmark gene sets in *SRF* fusion-positive samples. Tables and heatmaps showing that myogenesis and inflammation/interferon were among the top enriched pathways (**a**) in *SRF::E2F1*-transfected HEK293 and HT1080 and (**b**) in *SRF*-fusion-positive tumors (L107 and L108) vs *EWSR1*-rearranged MN (L161 and L162). NES: Normalized Enrichment Score; NOM p-val: Nominal *p*-value; FDR q-val: False Discovery Rate q-value.

Extending pathway analysis to the hallmarks gene set (GSEA hallmarks database), we found that the most significantly enriched biological processes in the cell lines transfected with *SRF::E2F1* were related to myogenesis, inflammation and IFN-response (Figure 5a). Real-time PCR analysis confirmed, in both HEK293 and HT1080 *SRF::E2F1* samples, a significant increase in myogenic marker expression (*ACTG2* and *CALD1*) when compared to all the control samples (Figure 4). Notably, the same hallmark pathways were upregulated in *SRF*-fusion positive myoepitheliomas (L107 and L108) with respect to *EWSR1*-fused MN (Figure 5b). Additional significantly enriched gene sets referring to immune activation and inflammation are shown in Supplementary Figure S1.

## 4. Discussion

**Figure 6:**
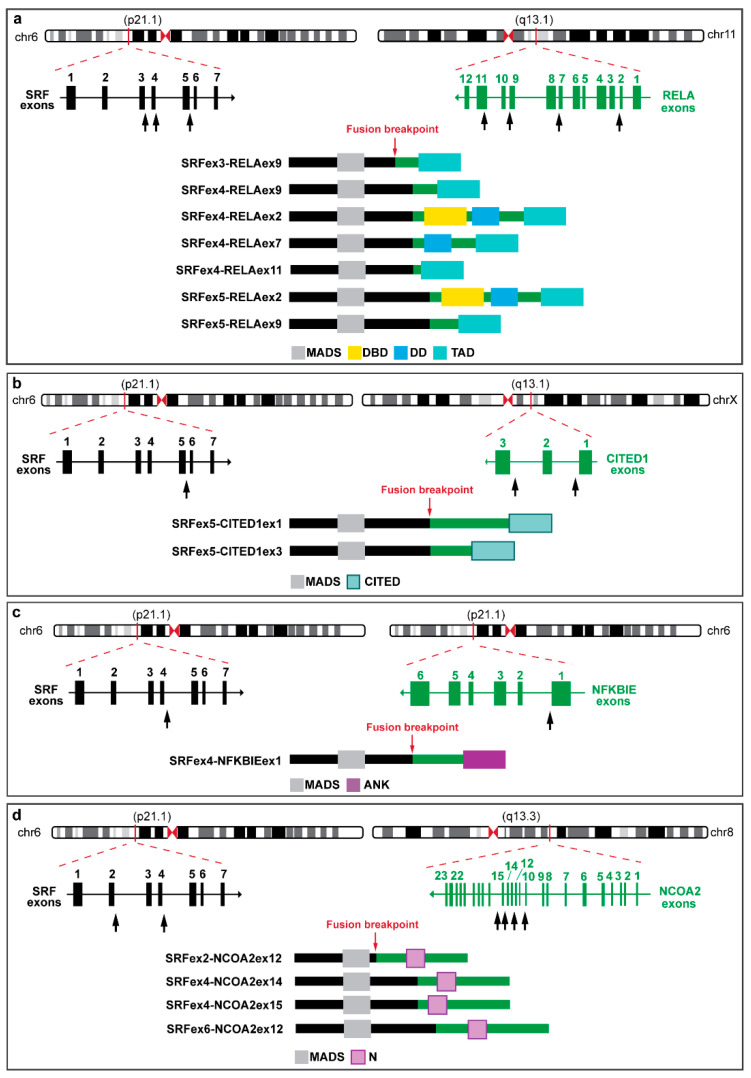
Schematic representation of *SRF* fusion transcripts and predicted chimeric proteins, depicting the exons and protein domains involved. The genomic exon structure of *SRF* and fusion partner genes is reported on the left and on the right, respectively: *SRF::RELA* (**a**) [4,5,6], *SRF::CITED1* (**b**) [5,9], *SRF::NFKBIE* (**c**) and *SRF::NCOA2* (**d**) [5,23,24]. Black arrows indicate breakpoints. Resulting chimeric proteins for each fusion transcript are reported at the bottom of each figure. MADS: MAD box DNA binding domain; DBD: DNA binding domain; DD: dimerization domain; TAD: transactivation domain; CITED: CBP/p300-interacting transactivator with ED-rich tail domain; ANK: ankyrin repeats and N: nuclear receptor coactivator.

The involvement of *SRF* in different rearrangement events has been identified in various soft tissue tumor types, as in perivascular myoid tumors and rhabdomyosarcomas. Little is known regarding gene fusions in myofibromas, and up to now the few cases analyzed led to the identification of recurrent *SRF* rearrangements with various 3′ partner genes in a subset of cellular variants of myofibroma [4,5]. *SRF* fusion events have been observed in myopericytomas, a group of tumors composed of relatively monomorphic, oval-spindle-shaped myoid-like cells, originating from perivascular myoid cells, and showing overlapping morphological features with myofibromas [22]. In particular, Antonescu and colleagues [4] detected *SRF* rearrangements in eight cases of myofibroma/myopericytoma (among which six cases had identical *SRF::RELA* fusions) displaying a significant downregulation of *SRF* mRNA (Figure 6a). These tumors exhibited a clear smooth-muscle-like immunophenotype showing diffuse reactivity and co-expression of SMA and desmin and abundant expression of actin and caldesmon.

Other perivascular myoid tumors harboring *SRF::RELA* fusions (Figure 6a) and displaying pericytic differentiation with expression of the smooth-muscle actin and caldesmon were newly described by Karanian and colleagues [5]. In addition, the histopathological and molecular features of three uterine tumors carrying *SRF::RELA* fusions (Figure 6a) were recently identified and compared to *SRF::RELA*-positive perivascular myoid tumors arising in other anatomical districts [6]. Also in this case, the immunohistochemical analysis showed desmin, caldesmon and SMA expression, supporting the myogenic commitment.

Other genetic alterations involving *SRF* gene fused to *CITED1*, *CITED2*, *NFKBIE* or *NCOA2* (Figure 6b–d) were observed in perivascular myoid tumors [5] and myofibromas [9]. Moreover, *SRF::NCOA2* fusion-positive pediatric RMS (rhabdomyosarcoma) showing diffuse staining for desmin and multifocal nuclear positivity for myogenin are reported (Figure 6d) [23,24].

**Figure 7:**
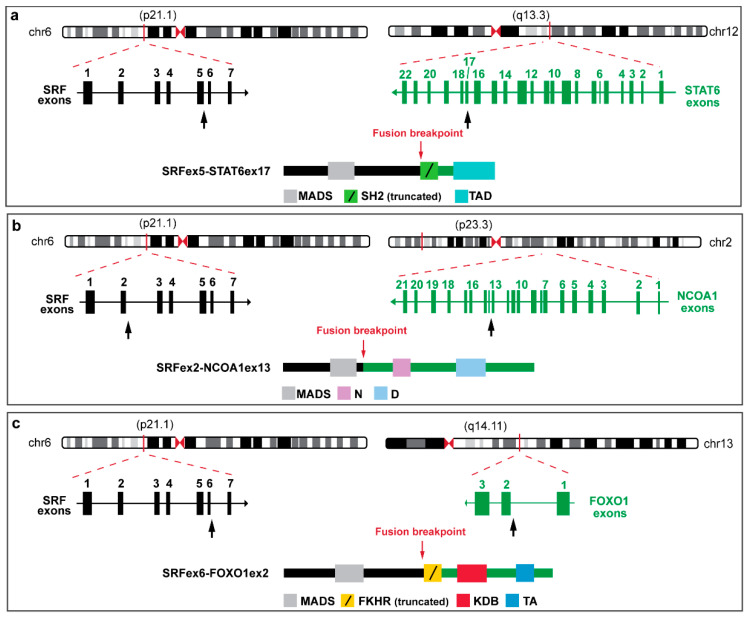
Schematic representation of *SRF*-fusion transcripts and predicted chimeric proteins, depicting the exons and protein domains involved. The genomic exon structure of the *SRF* and fusion partner genes are reported on the left and on the right, respectively: *SRF::STAT6* (**a**) [9], *SRF::NCOA1* (**b**) [8] and *SRF:: FOXO1* (**c**) [8]. Black arrows indicate breakpoints. Resulting chimeric proteins for each fusion transcript are reported at the bottom of each figure. MADS: MAD box DNA-binding domain; SH2: Src homology domain 2; TAD: transactivation domain; N: nuclear receptor coactivator; D: domain of unknown function; FKHR: forkhead DNA-binding domain; KBD: KIX-binding domain; (protein interacting domain) and TA: transcription activator domain.

A novel *SRF::ICA1L* fusion [7] was also found in a subset of cellular myofibromas of the deep soft tissues arising in adult patients. These *SRF*-fused tumors displayed an incomplete smooth muscle cell differentiation with a diffuse expression of SMA, calponin, and smooth-muscle-heavy myosin isoform, but no expression of desmin or caldesmon. Lastly, *SRF::STAT6* fusion (Figure 7a) was reported in a case of deep soft-tissue tumor of the arm in a 15-year-old boy, expressing a full smooth-muscle phenotype [25] and overlapping features with the *SRF::RELA* myofibromas [4]. Mitotic activity was low, and the tumor showed diffuse expression of α-SMA, desmin, caldesmon, and calponin, without expression of myogenin, MyoD1, CD34, EMA or PS100.

Recently, well-differentiated RMS with *SRF::NCOA1* (Figure 7b) or *SRF::FOXO1* (Figure 7c) fusions were reported in infantile localized paraspinal muscle tumors showing well-differentiated rhabdomyoblastic proliferations with nuclear atypia, infiltrative borders, and diffuse expression of desmin, myogenin, and MyoD1 [8]. Unsupervised gene expression analysis of these cases of *SRF*-fused RMSs and a cohort comprising different types of RMS showed that they clustered together and away from other skeletal muscle tumor types. Supervised gene expression analysis comparing *SRF*-fused RMSs with other types of RMS, identified a strong upregulation of genes involved in muscle differentiation and function, and a downregulation of cell cycle/proliferation pathways. A cell model of this specific fusion was recently established from an infantile spindle cell RMS tumor harboring *SRF::NCOA2* gene fusion, confirming the rhabdomyoblastic features shown by MYOD1 and myogenin expression [14].

Overall, *SRF*-fusion transcripts described in the literature mostly retain the MAD domain of *SRF* necessary for the target genes binding, and the C-terminal transcription activating domains of partner genes. This is the case with *SRF::RELA* [4,5], *SRF::ICA1L* [7,9], *SRF::NCOA2* [5] and *SRF::STAT6* [25] neoplasms, which present similar *SRF* breakpoints. Therefore, in these *SRF*-fused tumors, aberrant transcription factors are produced, and consequently, pathways involving *SRF* are enriched [5]. Exploration of the follow-up data regarding *SRF*-fused tumors demonstrates that neoplasms harboring *SRF* rearrangements have a generally benign behavior, regardless of *SRF* partner genes.

Here, we further investigated the novel *SRF::E2F1* fusion in myoepitheliomas, which, together with the mixed tumors/chondroid syringomas, represent a class of myoepithelial tumors of soft tissue with benign behavior [26]. In these samples, the expression of SRF::E2F1 transcript led to the production of a functionally active chimeric protein [10]. Bioinformatics analyses performed herein revealed a distinct expression profile of MN harboring *SRF* rearrangement with respect to *EWSR1* fusion-positive tumors, with the overexpression of *SRF* target genes as the immediate early genes *EGR2*, *EGR3*, *EGR4*, *FOS* and *FOSB*. This specific *SRF* signature is shared by tumor subtypes carrying other *SRF*-fused partner genes, such as the perivascular tumors harboring *SRF::RELA* fusion [5]. Indeed, GSEA performed by Karanian and colleagues on *SRF::RELA* tumors [5] revealed that the top enriched gene sets were the SRF binding sites, similar to our *SRF::E2F1* MN cases.

Additionally, myoepithelial tumors harboring *SRF::E2F1* fusion displayed a strong overexpression of genes involved in myogenic differentiation, as myosin heavy/light chain, troponin and actin gene families; some of these genes were also identified as differentially expressed in *SRF*-fused perivascular tumors [5].

Interestingly, we observed an upregulation of genes involved in inflammation and immune response in *SRF* fusion-positive myoepitheliomas with respect to *EWSR1*-fused MN. These results seem to be in line with the few reports showing *SRF*’s role in immune activation [27,28,29,30,31], despite that *SRF* involvement in the regulation of inflammation and immunity has not been systematically explored yet. As proof, the comparison between the differentially expressed genes in *SRF::RELA* tumors [5] and our *SRF::E2F1* myoepitheliomas showed 356 overlapping genes, including those involved in myogenesis (*MYH11*, *TAGLN* and *MYLK*), inflammation (*TNSF10*, *IFI27*, *CXCL11*, *BST2*and *IFI44L*) and the immediate early genes *EGR2* and *EGR4*. The specificity of gene expression signature induced by the fusion transcript in myoepitheliomas was confirmed by bioinformatics analyses on HEK293 and HT1080 transiently expressing *SRF::E2F1* fusion.

Of note, the *SRF::E2F1*-positive MN carrying the *FUS* rearrangement (L108) could display a specific genetic signature induced by the *FUS::KLF17* fusion. However, the reduced number of cases in the *SRF::E2F1* group and the lack of published data concerning the *FUS::KLF1*7-activated pathways prevents the identification of a specific gene expression profile determined by *FUS* rearrangement in this sample.

**Figure 8:**
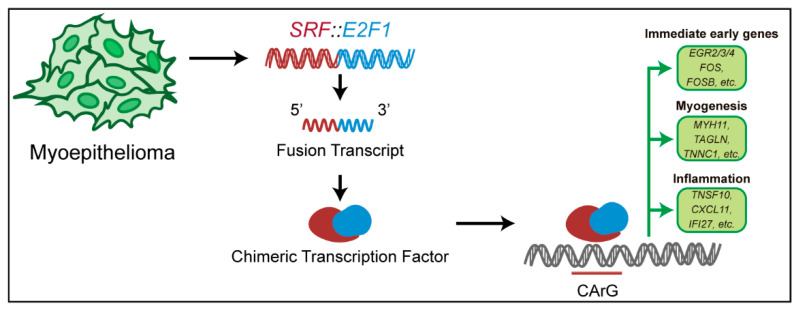
Schematic representation of the proposed pathways involving *SRF::E2F1*. In *SRF* fusion-positive myoepitheliomas, the *SRF::E2F1* fusion encodes a chimeric transcript, and consequently, an aberrant transcription factor is produced. The chimeric protein binds the CArG sequence on the DNA, inducing the expression of immediate early genes and those involved in myogenesis and inflammation.

This work proves the central role of the SRF chimeric fusion protein in inducing the myogenic differentiation program in *SRF*-fused tumors, as well as the inflammatory and immune signature, with the upregulation of the Interferon-induced genes (Figure 8).

In conclusion, it is tempting to speculate that the relatively benign behavior of this molecular subtype of myoid tumors could at least be partly dependent on the induction of an inflammatory response and immune recognition. The identification of the specific gene signature driven by *SRF* fusion could aid the molecular diagnostic process and guide the therapeutic decisions with respect to the clinical behavior of this rare tumor entity.

## Data Availability

The data presented in this study are openly available at the SRA repository (https://www.ncbi.nlm.nih.gov/sra/) with the bioproject accession number PRJNA881928.

## Supplementary Materials

The following supporting information can be downloaded at: https://www.mdpi.com/article/10.3390/biom12111678/s1, Table S1: Differentially expressed genes between SRF::E2F1-transfected cells and controls (logFC = log2 fold change; logCPM = log2 counts/million).; Table S2: Differentially expressed genes between *SRF*-Fused and *EWSR1*-Fused tumors (logFC = log2 fold change; logCPM = log2 counts/million); Figure S1: GSEA analyses of *SRF::E2F1* myoepitheliomas versus *EWSR1*-Fused positive tumors.
