# Supplementary material for: SRF Rearrangements in Soft Tissue Tumors with Muscle Differentiation
Source: Biomolecules. 2022 Nov 12;12(11):1678. doi: 10.3390/biom12111678 (PMC9687304; doi:10.3390/biom12111678)
Supplement: Supplementary file 1 [file biomolecules-12-01678-s001.zip › Supplementary Figure S1.pdf]

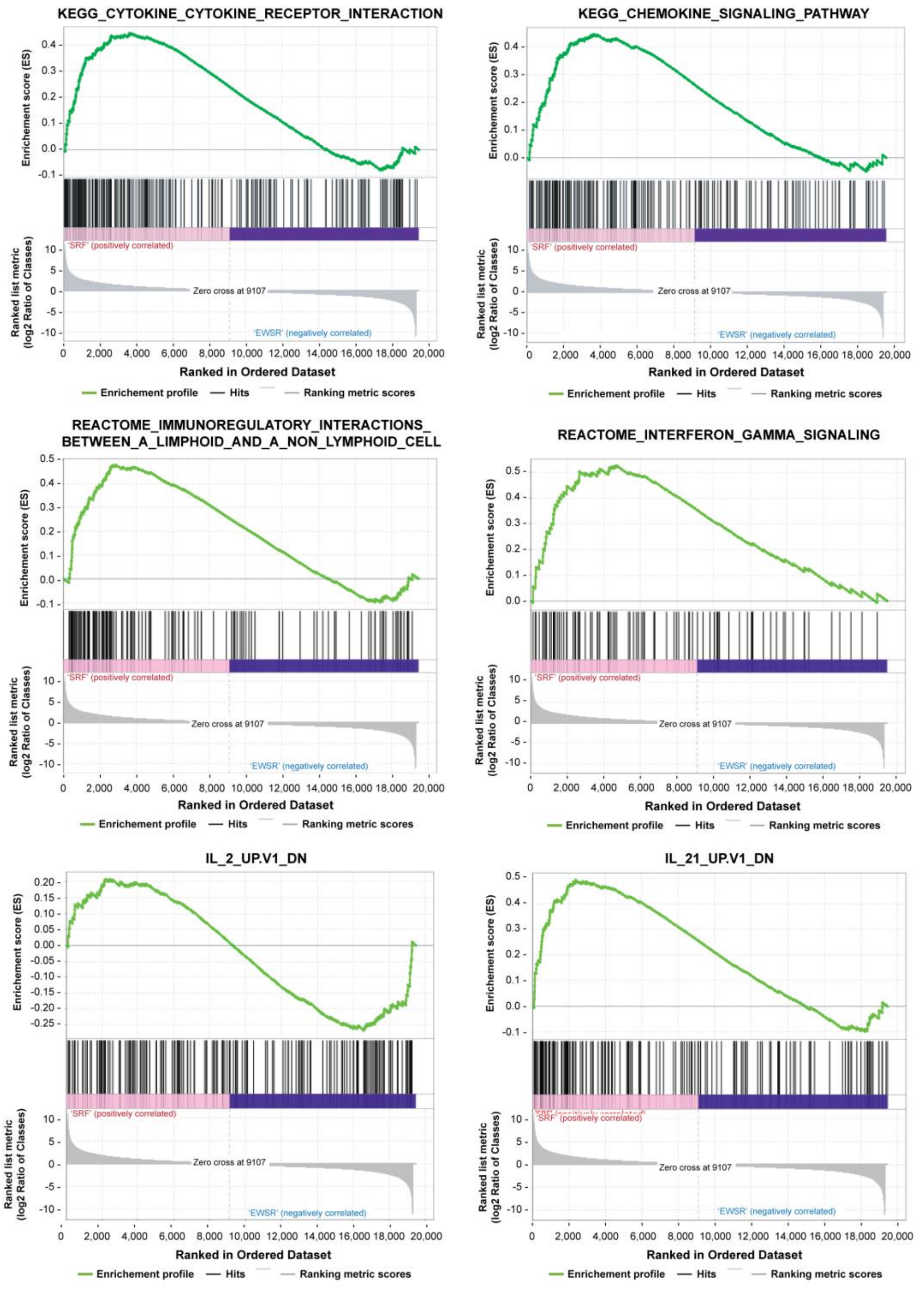

Gene Set	SIZE	NES	NOM p-val	FDR q-val
CYTOKINE_CYTOKINE_RECEPTOR_INTERACTION	199	1,89	0,0	8,84E-03
CHEMOKINE_SIGNALING_PATHWAY	169	1,84	0,0	1,31E-02
REACTOME_IMMUNOREGULATORY_INTERACTION_BETWEEN_A_LYMPHOID_AND_A_NON_LYMPHOID_CELL	133	1,91	0,0	1,42E-02
REACTOME_INTERFERON_GAMMA_SIGNALING	89	2,00	0,0	3,86E-03
IL_2_UP.V1_DN	170	-1,21	0,0	8,27E-01
IL_21_UP.V1_DN	159	2,04	0,0	0,0

Supplementary Figure S1. **GSEA analyses of *SRF::E2F1* myoepitheliomas versus *EWSR1*-fused positive tumors**. **a**) Enrichment curves of the significantly enriched gene sets referring to immune activation and inflammation.NES: Normalized Enrichment Scores NOM p-val: Nominal p-value; FDR q-val: False Discovery Rate q-value
