# Supplementary material for: SRF Rearrangements in Soft Tissue Tumors with Muscle Differentiation
Source: Biomolecules. 2022 Nov 12;12(11):1678. doi: 10.3390/biom12111678 (PMC9687304; doi:10.3390/biom12111678)
Supplement: Supplementary file 1 [file biomolecules-12-01678-s001.zip › Supplementary Table S1.pdf]

Supplementary Table S1. Differentially expressed genes between *SRF::E2F1*-transfected cells and controls (logFC=log2 fold change; logCPM=log2 counts/million).

GENE SYMBOL	logFC	logCPM	PValue	GENE ID
EGR1	4,99	7,08	1,49E-12	ENSG00000120738
EGR4	10,31	3,94	4,56E-10	ENSG00000135625
SRF	3,79	8,85	1,79E-09	ENSG00000112658
RPE65	10,54	3,46	2,04E-09	ENSG00000116745
KRT17	6,58	3,96	1,25E-08	ENSG00000128422
TCHH	7,63	3,76	1,69E-08	ENSG00000159450
A2M	8,23	5,40	6,98E-08	ENSG00000175899
HSH2D	8,05	2,34	2,19E-07	ENSG00000196684
GBA3	9,72	1,25	7,81E-07	ENSG00000249948
TAGLN	5,22	6,29	1,18E-06	ENSG00000149591
HJV	9,65	1,18	1,26E-06	ENSG00000168509
WFDC12	9,00	0,57	2,59E-06	ENSG00000168703
ACTA2	3,32	6,08	3,24E-06	ENSG00000107796
XIRP1	8,11	1,10	3,54E-06	ENSG00000168334
ASPA	7,49	2,90	4,49E-06	ENSG00000108381
JAML	7,53	1,25	4,77E-06	ENSG00000160593
GPR183	7,72	2,12	5,07E-06	ENSG00000169508
E2F1	2,93	7,73	5,62E-06	ENSG00000101412
ACTG2	7,17	1,75	6,33E-06	ENSG00000163017
FMO3	8,97	0,55	6,52E-06	ENSG00000007933
KRT12	9,51	1,05	6,77E-06	ENSG00000187242
OPALIN	9,12	0,69	7,71E-06	ENSG00000197430
DNAJA4	7,87	1,05	8,09E-06	ENSG00000140403
ACTC1	7,83	1,90	9,71E-06	ENSG00000159251
AC079313.2	7,85	-0,44	1,11E-05	ENSG00000258137
KRT9	7,73	-0,55	1,28E-05	ENSG00000171403
C1orf194	7,85	-0,44	1,30E-05	ENSG00000179902
FRG2FP	7,19	1,48	1,53E-05	ENSG00000232783
PRSS58	7,34	1,07	1,64E-05	ENSG00000258223
MYL1	7,27	1,49	2,21E-05	ENSG00000168530
GLYATL1P1	7,59	-0,66	2,28E-05	ENSG00000255189
AL596330.1	7,55	-0,69	2,31E-05	ENSG00000229400
DNAJA1P5	7,70	-0,57	2,32E-05	ENSG00000162699
TNMD	7,67	3,00	3,16E-05	ENSG00000000005
AP000892.3	6,84	2,80	3,39E-05	ENSG00000280143
MB	7,50	-0,73	3,79E-05	ENSG00000198125
TDRD9	7,83	-0,46	4,43E-05	ENSG00000156414
AC008074.2	5,11	2,45	4,83E-05	ENSG00000260101
AL160171.1	7,63	-0,63	5,07E-05	ENSG00000285923
TLR7	5,47	-0,16	5,22E-05	ENSG00000196664
A1CF	6,51	0,26	5,25E-05	ENSG00000148584
KRT85	8,21	-0,13	5,40E-05	ENSG00000135443
ITIH3	6,19	0,95	5,75E-05	ENSG00000162267
MYH6	8,06	-0,27	6,28E-05	ENSG00000197616
LRRC19	7,63	-0,63	6,37E-05	ENSG00000184434
TLR8-AS1	7,95	-0,36	6,59E-05	ENSG00000233338
AL513321.2	6,89	-1,20	8,48E-05	ENSG00000282906
IL33	5,70	4,39	9,22E-05	ENSG00000137033
EGR2	4,97	2,36	9,83E-05	ENSG00000122877
ROS1	5,37	0,61	1,01E-04	ENSG00000047936
ITLN2	7,44	-0,78	1,02E-04	ENSG00000158764
ATP1B4	6,91	0,00	1,20E-04	ENSG00000101892
MYL7	6,02	-0,70	1,37E-04	ENSG00000106631
LINC02153	6,63	-1,38	1,37E-04	ENSG00000253199
FRMD7	6,26	-0,51	1,40E-04	ENSG00000165694
AL807740.1	6,77	-1,28	1,40E-04	ENSG00000283400
GRM1	5,18	1,62	1,41E-04	ENSG00000152822
IGSF22	5,30	-0,29	1,47E-04	ENSG00000179057
VCAM1	7,17	-0,99	1,55E-04	ENSG00000162692
PLN	7,33	2,11	1,69E-04	ENSG00000198523

TBATA	5,76	-0,94	1,82E-04	ENSG00000166220
CHRM1	6,48	-1,48	1,92E-04	ENSG00000168539
LINC01010	6,83	-0,08	1,92E-04	ENSG00000236700
LMNTD1	5,43	0,14	1,97E-04	ENSG00000152936
NPPB	6,48	-1,48	2,05E-04	ENSG00000120937
CD52	5,75	-0,89	2,06E-04	ENSG00000169442
CXCL11	5,37	5,42	2,59E-04	ENSG00000169248
OPN5	4,78	-0,68	2,63E-04	ENSG00000124818
CEACAM21	6,39	-1,54	2,64E-04	ENSG00000007129
TRPC7	6,31	-1,59	2,67E-04	ENSG00000069018
ARC	4,19	4,41	2,85E-04	ENSG00000198576
KRT1	5,70	-0,98	3,10E-04	ENSG00000167768
TRDN	4,98	-0,98	3,36E-04	ENSG00000186439
CARMN	6,22	-1,65	3,38E-04	ENSG00000249669
PZP	6,31	-1,59	3,50E-04	ENSG00000126838
ATP13A4	5,31	-1,26	4,21E-04	ENSG00000127249
HEPHL1	6,31	-1,59	4,32E-04	ENSG00000181333
GP5	4,87	-1,06	4,40E-04	ENSG00000178732
EIF4A1P9	5,49	-1,08	4,85E-04	ENSG00000262953
C9orf84	4,53	-1,27	4,88E-04	ENSG00000165181
ERG	5,83	2,39	5,00E-04	ENSG00000157554
COL1A2	5,33	-0,18	5,08E-04	ENSG00000164692
AC091152.4	4,34	1,84	5,26E-04	ENSG00000279879
KLHL10	5,32	1,70	5,36E-04	ENSG00000161594
C9	6,12	-1,71	5,39E-04	ENSG00000113600
AC083949.1	4,46	-0,60	6,03E-04	ENSG00000224875
AL355607.2	6,08	2,60	6,10E-04	ENSG00000260454
C11orf16	6,12	-1,71	6,20E-04	ENSG00000176029
MCF2	4,47	2,54	6,37E-04	ENSG00000101977
FAM71F1	5,41	0,35	6,55E-04	ENSG00000135248
RDH8	5,24	-1,25	7,37E-04	ENSG00000080511
LINC02244	5,24	-1,31	7,71E-04	ENSG00000259590
AC103760.1	4,35	0,59	7,87E-04	ENSG00000254231
TGM3	5,38	-1,16	7,89E-04	ENSG00000125780
AC074131.1	6,21	-1,65	8,01E-04	ENSG00000250831
MYOZ2	4,69	-0,18	8,22E-04	ENSG00000172399
AL391335.1	5,04	-0,87	8,71E-04	ENSG00000250734
SLC16A6	3,73	3,22	8,89E-04	ENSG00000108932
TNIP3	4,91	1,22	9,44E-04	ENSG00000050730
LRRC53	5,90	-1,83	9,50E-04	ENSG00000162621
KIAA1210	4,56	-1,28	1,02E-03	ENSG00000250423
ALOX5AP	4,13	-1,15	1,07E-03	ENSG00000132965
CNN1	4,82	0,04	1,09E-03	ENSG00000130176
NCAM2	5,32	-1,20	1,10E-03	ENSG00000154654
PCDHB17P	4,90	-1,49	1,12E-03	ENSG00000255622
FCGR3A	6,02	-1,77	1,15E-03	ENSG00000203747
COL11A1	4,12	-0,02	1,16E-03	ENSG00000060718
KRT14	5,17	-1,35	1,19E-03	ENSG00000186847
KCNMB1	5,78	-1,90	1,27E-03	ENSG00000145936
LINC02599	4,85	-1,56	1,27E-03	ENSG00000233858
AOX2P	5,21	-1,30	1,29E-03	ENSG00000243478
AC009812.2	5,78	-1,90	1,40E-03	ENSG00000253760
LGI1	4,79	0,18	1,49E-03	ENSG00000108231
SPATA31E1	4,99	-1,44	1,49E-03	ENSG00000177992
FYB2	4,72	-1,61	1,55E-03	ENSG00000187889
AC119674.1	5,64	-1,97	1,68E-03	ENSG00000260971
CRYAB	4,43	-1,36	1,68E-03	ENSG00000109846
LCP2	5,01	0,59	1,73E-03	ENSG00000043462
CXCL10	4,85	2,35	1,82E-03	ENSG00000169245
BOLL	4,51	-1,26	1,87E-03	ENSG00000152430
CD36	5,64	2,31	1,91E-03	ENSG00000135218
GABRR1	5,20	-0,05	1,95E-03	ENSG00000146276
TMIGD3	4,41	-1,25	1,98E-03	ENSG00000121933
TPH1	4,08	1,88	1,99E-03	ENSG00000129167

ENDOU	5,16	1,64	2,02E-03	ENSG00000111405
AL359762.1	4,30	-0,28	2,07E-03	ENSG00000284882
CCL22	5,65	-1,97	2,09E-03	ENSG00000102962
AC080080.1	3,75	1,51	2,12E-03	ENSG00000279048
FER1L6	4,46	0,08	2,14E-03	ENSG00000214814
TRIM54	4,06	0,46	2,14E-03	ENSG00000138100
CASQ1	4,23	-0,13	2,19E-03	ENSG00000143318
AC245297.1	4,03	-0,21	2,22E-03	ENSG00000215861
GALNT15	4,52	0,25	2,31E-03	ENSG00000131386
TG	3,49	-1,08	2,37E-03	ENSG00000042832
SLC44A4	4,66	-1,67	2,44E-03	ENSG00000204385
CMKLR1	4,63	-1,66	2,55E-03	ENSG00000174600
DEC1	3,91	-1,26	2,76E-03	ENSG00000173077
MYL4	3,87	-0,54	2,90E-03	ENSG00000198336
BSND	4,60	-1,66	3,22E-03	ENSG00000162399
CERKL	4,48	0,34	3,25E-03	ENSG00000188452
AC026310.2	3,79	-0,78	3,29E-03	ENSG00000255921
SLC22A10	5,49	-2,05	3,45E-03	ENSG00000184999
AL136979.1	5,49	-2,05	3,47E-03	ENSG00000227914
OR52N2	5,49	-2,05	3,47E-03	ENSG00000180988
AC092818.1	4,04	-0,01	3,56E-03	ENSG00000254038
MYOZ1	4,26	0,38	3,71E-03	ENSG00000177791
DNAI1	3,49	-0,22	3,71E-03	ENSG00000122735
KCNK3	4,67	-1,67	3,85E-03	ENSG00000171303
DIRC3	4,50	1,21	3,86E-03	ENSG00000231672
AC233724.5	5,50	-2,05	3,88E-03	ENSG00000248542
AC084816.1	3,87	-1,26	3,96E-03	ENSG00000256995
DNAH12	5,03	0,88	4,64E-03	ENSG00000174844
LINC01556	3,68	-1,48	4,80E-03	ENSG00000204709
GRM3	3,79	-1,37	4,91E-03	ENSG00000198822
COL5A3	3,90	-0,47	5,05E-03	ENSG00000080573
LDB3	3,28	-0,11	5,08E-03	ENSG00000122367
ZEB2-AS1	4,07	-1,50	5,14E-03	ENSG00000238057
PTGS1	3,80	0,29	5,21E-03	ENSG00000095303
NT5E	3,62	1,77	5,28E-03	ENSG00000135318
LIMS2	3,50	0,84	5,40E-03	ENSG00000072163
TEX41	5,33	-2,13	5,40E-03	ENSG00000226674
AC099329.2	4,44	-1,79	5,41E-03	ENSG00000273328
ADAM5	5,33	-2,12	5,43E-03	ENSG00000196115
AL139125.2	3,90	-1,38	5,48E-03	ENSG00000278982
AIF1	4,45	-1,79	5,49E-03	ENSG00000204472
FSD2	4,27	-0,78	5,68E-03	ENSG00000186628
LCN1	4,03	-1,23	5,70E-03	ENSG00000160349
PLCXD3	4,21	0,96	5,76E-03	ENSG00000182836
THSD7B	4,19	-1,83	5,89E-03	ENSG00000144229
CD74	3,58	0,10	6,03E-03	ENSG00000019582
MYH11	3,88	-0,82	6,31E-03	ENSG00000133392
AC243571.2	3,70	-1,29	6,69E-03	ENSG00000277501
SEPT7P9	4,27	-1,85	6,85E-03	ENSG00000120555
ADAMTS4	3,87	0,96	6,87E-03	ENSG00000158859
C1orf61	4,16	1,10	6,90E-03	ENSG00000125462
OOSP3	4,07	-1,50	6,90E-03	ENSG00000285231
PRKCG	4,30	1,61	7,02E-03	ENSG00000126583
TEKT5	3,44	-0,82	7,15E-03	ENSG00000153060
MTMR7	3,70	3,81	7,38E-03	ENSG00000003987
EXOC3L2	3,67	-0,50	7,57E-03	ENSG00000283632
CMAHP	4,45	2,41	7,63E-03	ENSG00000168405
SHD	4,32	0,02	7,66E-03	ENSG00000105251
AL390037.1	3,83	-1,60	7,68E-03	ENSG00000277901
LINC00923	5,14	-2,21	8,04E-03	ENSG00000251209
CALHM4	5,14	-2,21	8,05E-03	ENSG00000164451
RHOXF1-AS1	5,14	-2,21	8,05E-03	ENSG00000258545
AP003472.1	5,14	-2,21	8,07E-03	ENSG00000253824
AC126178.1	5,14	-2,21	8,08E-03	ENSG00000257194

IFI44L	3,28	2,70	8,30E-03	ENSG00000137959
TYRP1	3,99	-0,44	8,39E-03	ENSG00000107165
EGF	3,15	2,77	8,42E-03	ENSG00000138798
GRIN2B	3,83	-1,68	8,73E-03	ENSG00000273079
KLHL41	3,81	0,54	1,00E-02	ENSG00000239474
OR52K3P	3,53	-0,17	1,02E-02	ENSG00000225101
A2MP1	3,25	-1,50	1,05E-02	ENSG00000256069
AC006994.2	4,49	-0,58	1,07E-02	ENSG00000279317
TUBB8P8	3,88	-1,61	1,09E-02	ENSG00000213113
METTL11B	3,97	-0,01	1,10E-02	ENSG00000203740
TESPA1	3,62	-1,72	1,12E-02	ENSG00000135426
GPA33	4,38	0,82	1,13E-02	ENSG00000143167
CES1P2	5,16	-0,08	1,15E-02	ENSG00000260765
CASS4	3,43	1,10	1,19E-02	ENSG00000087589
FHL5	3,43	-1,12	1,20E-02	ENSG00000112214
AL033527.2	3,45	-1,39	1,22E-02	ENSG00000236546
FOXS1	3,50	-0,75	1,23E-02	ENSG00000179772
DLG2	2,90	1,50	1,25E-02	ENSG00000150672
CRAT37	4,92	-2,29	1,25E-02	ENSG00000258551
TIMD4	4,92	-2,29	1,25E-02	ENSG00000145850
DAOA-AS1	4,92	-2,29	1,25E-02	ENSG00000232307
AL356108.1	4,92	-2,29	1,26E-02	ENSG00000236230
AC068205.1	4,92	-2,29	1,26E-02	ENSG00000283217
ANKUB1	3,61	-1,46	1,26E-02	ENSG00000206199
PKHD1	4,00	-1,42	1,31E-02	ENSG00000170927
AC092919.1	3,69	-1,74	1,35E-02	ENSG00000240497
JCHAIN	3,25	-1,70	1,37E-02	ENSG00000132465
KCNIP2-AS1	2,77	-1,02	1,40E-02	ENSG00000226009
NPHP3-AS1	3,09	-0,69	1,40E-02	ENSG00000248724
HNRNPA1P11	3,48	-1,78	1,41E-02	ENSG00000264315
AC004990.1	3,80	-1,68	1,42E-02	ENSG00000232756
BANK1	4,03	-2,00	1,46E-02	ENSG00000153064
CARD16	3,70	-1,74	1,46E-02	ENSG00000204397
AKR1B15	3,33	-1,63	1,48E-02	ENSG00000227471
AC090515.2	2,82	-0,31	1,50E-02	ENSG00000245975
ADNP-AS1	2,90	1,00	1,53E-02	ENSG00000259456
AL442663.3	2,97	1,21	1,53E-02	ENSG00000258813
APOE	3,42	2,79	1,55E-02	ENSG00000130203
AL596220.1	3,36	-0,97	1,55E-02	ENSG00000229739
NECAB1	3,01	-0,79	1,55E-02	ENSG00000123119
SLC6A12	3,07	-0,55	1,62E-02	ENSG00000111181
RPL7P38	3,07	-1,45	1,65E-02	ENSG00000239473
HOMER1	1,52	6,13	1,66E-02	ENSG00000152413
MALINC1	2,63	2,96	1,67E-02	ENSG00000245146
TRIM55	3,13	0,43	1,68E-02	ENSG00000147573
SLC12A8	3,45	2,45	1,70E-02	ENSG00000221955
OTOGL	2,67	-0,82	1,71E-02	ENSG00000165899
GZMA	4,01	1,32	1,73E-02	ENSG00000145649
ITK	3,21	-1,41	1,73E-02	ENSG00000113263
GJC3	3,19	0,60	1,74E-02	ENSG00000176402
LINC02392	4,82	2,18	1,76E-02	ENSG00000258183
FOSB	2,65	2,64	1,78E-02	ENSG00000125740
CCDC190	3,66	0,39	1,87E-02	ENSG00000185860
LINC02228	2,72	-0,14	1,92E-02	ENSG00000251273
C6orf163	3,21	0,73	1,93E-02	ENSG00000203872
ABCA4	2,84	-0,55	1,97E-02	ENSG00000198691
USH2A	3,16	-0,66	1,97E-02	ENSG00000042781
IMPDH1P10	3,60	1,28	2,01E-02	ENSG00000232133
C3orf38	1,40	6,34	2,06E-02	ENSG00000179021
TSPAN19	2,78	1,68	2,09E-02	ENSG00000231738
ALDH1A1	2,76	-0,26	2,11E-02	ENSG00000165092
AC010834.2	2,97	-1,81	2,12E-02	ENSG00000253848
ABCD2	3,17	2,07	2,13E-02	ENSG00000173208
AC105339.3	3,74	-2,04	2,18E-02	ENSG00000259442

LINC00907	3,74	-2,04	2,18E-02	ENSG00000267586
RERGL	3,21	-1,41	2,19E-02	ENSG00000111404
AC100803.2	2,95	-1,33	2,19E-02	ENSG00000261655
CCDC81	3,07	0,71	2,20E-02	ENSG00000149201
FILIP1L	3,93	3,16	2,22E-02	ENSG00000168386
FLG	3,21	1,59	2,28E-02	ENSG00000143631
GRIN2A	2,97	-1,59	2,30E-02	ENSG00000183454
LRTM2	3,40	-1,57	2,38E-02	ENSG00000166159
PTGS2	2,52	2,65	2,39E-02	ENSG00000073756
AC087482.1	2,68	-0,65	2,45E-02	ENSG00000259347
MYO7B	3,43	-1,87	2,51E-02	ENSG00000169994
ENTPD1	2,66	-0,73	2,61E-02	ENSG00000138185
AC090197.1	3,06	-1,65	2,62E-02	ENSG00000253837
PDZPH1P	2,90	-1,57	2,62E-02	ENSG00000226926
MAMDC2	3,07	0,05	2,66E-02	ENSG00000165072
B3GALT2	3,69	-0,52	2,67E-02	ENSG00000162630
SLC15A2	2,71	1,08	2,67E-02	ENSG00000163406
EGR3	4,46	2,73	2,71E-02	ENSG00000179388
CFLAR-AS1	3,25	0,79	2,80E-02	ENSG00000226312
AL391001.1	2,83	-1,08	2,82E-02	ENSG00000269887
POMC	3,49	-0,82	2,85E-02	ENSG00000115138
RRAD	2,83	-0,63	2,91E-02	ENSG00000166592
KLHL1	3,65	-2,14	2,91E-02	ENSG00000150361
GNG12-AS1	2,98	0,67	2,91E-02	ENSG00000232284
MGAM	2,64	1,05	2,94E-02	ENSG00000257335
CALHM5	1,88	4,84	2,96E-02	ENSG00000178033
DRC1	3,30	-1,94	3,02E-02	ENSG00000157856
AC112503.2	3,30	-1,94	3,02E-02	ENSG00000273454
AC104653.1	2,45	-1,12	3,06E-02	ENSG00000228857
CCL26	3,59	-1,46	3,17E-02	ENSG00000006606
RYR3	3,17	-0,77	3,18E-02	ENSG00000198838
CYP19A1	3,00	-2,10	3,19E-02	ENSG00000137869
KDM3A	1,29	6,72	3,30E-02	ENSG00000115548
DOK7	3,24	0,11	3,32E-02	ENSG00000175920
LHFPL1	3,24	-1,92	3,36E-02	ENSG00000182508
SPEF1	2,51	0,46	3,38E-02	ENSG00000101222
ITGB1BP2	2,40	0,29	3,46E-02	ENSG00000147166
CHRNA9	2,84	-0,54	3,47E-02	ENSG00000174343
AC092650.1	2,81	-1,88	3,83E-02	ENSG00000230773
PTGES3L	2,39	-0,44	3,85E-02	ENSG00000267060
LRIT3	-5,46	-1,46	3,92E-02	ENSG00000183423
C12orf50	2,67	-1,41	3,92E-02	ENSG00000165805
APOC1P1	3,40	-1,56	3,94E-02	ENSG00000214855
ENTPD3	2,91	-1,51	3,97E-02	ENSG00000168032
EXOC5P1	2,41	0,31	3,97E-02	ENSG00000180673
COL28A1	2,89	-1,52	3,98E-02	ENSG00000215018
GSN-AS1	2,74	-2,17	3,98E-02	ENSG00000235865
KRT18P31	2,56	-1,45	4,02E-02	ENSG00000249850
PRKG1-AS1	2,54	-0,98	4,10E-02	ENSG00000236671
NCKAP1L	2,66	1,15	4,16E-02	ENSG00000123338
TEK	2,82	0,90	4,17E-02	ENSG00000120156
PTH	3,29	-1,15	4,18E-02	ENSG00000152266
ANKRD30B	2,74	-1,29	4,22E-02	ENSG00000180777
MYO1A	2,70	-1,54	4,30E-02	ENSG00000166866
PIFO	2,98	0,19	4,33E-02	ENSG00000173947
PLA1A	3,03	-1,98	4,33E-02	ENSG00000144837
GAPDHP46	3,47	-2,23	4,33E-02	ENSG00000224678
TNC	1,44	7,06	4,36E-02	ENSG00000041982
SUSD4	2,40	0,94	4,38E-02	ENSG00000143502
ANGPTL1	2,42	-0,59	4,39E-02	ENSG00000116194
SPAG17	3,17	0,05	4,45E-02	ENSG00000155761
AKAP6	2,28	2,36	4,47E-02	ENSG00000151320
HGD	2,69	-1,96	4,51E-02	ENSG00000113924
FRG2B	2,76	-1,62	4,53E-02	ENSG00000225899

RGS22	2,45	-1,00	4,55E-02	ENSG00000132554
CHORDC2P	2,28	-1,33	4,57E-02	ENSG00000259050
AP003108.3	-5,27	-1,58	4,59E-02	ENSG00000279246
AC004854.2	2,33	0,58	4,60E-02	ENSG00000272768
LINC00595	2,64	-0,46	4,66E-02	ENSG00000230417
DUOX1	2,74	-1,86	4,75E-02	ENSG00000137857
CPO	2,78	-1,88	4,76E-02	ENSG00000144410
AL831711.1	2,47	-1,43	4,76E-02	ENSG00000283317
ZFHX4-AS1	2,47	0,05	4,87E-02	ENSG00000253661
LINC02458	3,39	0,43	4,92E-02	ENSG00000246363
LINC00639	-6,42	-0,77	4,98E-02	ENSG00000259070
MX1	2,57	3,63	5,00E-02	ENSG00000157601
LINGO2	2,51	2,79	5,00E-02	ENSG00000174482
NUDT17	1,95	3,70	5,01E-02	ENSG00000186364
CALD1	1,34	7,90	5,04E-02	ENSG00000122786
