# Supplementary material for: SRF Rearrangements in Soft Tissue Tumors with Muscle Differentiation
Source: Biomolecules. 2022 Nov 12;12(11):1678. doi: 10.3390/biom12111678 (PMC9687304; doi:10.3390/biom12111678)
Supplement: Supplementary file 1 [file biomolecules-12-01678-s001.zip › Supplementary Table S2.pdf]

Supplementary Table S2. Differentially expressed genes between *SRF-Fused* and *EWSR1-Fused* tumors (logFC=log2 fold change; logCPM=log2 counts/ million).

GENE SYMBOL	logFC	logCPM	PValue	GENE ID
KRT12	11,06	7,94	1,12E-11	ENSG00000187242
MPZ	9,24	10,36	1,36E-11	ENSG00000158887
KLHDC8A	8,79	6,38	3,40E-11	ENSG00000162873
STMN2	8,07	6,89	4,87E-11	ENSG00000104435
NCMAP	13,39	6,52	6,19E-11	ENSG00000184454
SOX10	9,44	6,76	3,16E-10	ENSG00000100146
AC004231.3	12,77	5,91	3,17E-10	ENSG00000265359
DCSTAMP	12,55	5,68	4,13E-10	ENSG00000164935
PLP1	9,23	8,57	5,03E-10	ENSG00000123560
TACSTD2	8,67	9,19	6,31E-10	ENSG00000184292
RPE65	12,70	5,84	1,03E-09	ENSG00000116745
PRIMA1	8,23	5,80	1,06E-09	ENSG00000175785
CALY	9,81	6,69	1,17E-09	ENSG00000130643
ARC	7,92	6,34	1,50E-09	ENSG00000198576
CLVS2	-12,10	5,24	2,00E-09	ENSG00000146352
NGFR	8,23	8,30	3,60E-09	ENSG00000064300
ZFP42	12,34	5,47	4,21E-09	ENSG00000179059
FGFBP2	12,08	5,22	1,23E-08	ENSG00000137441
SCN2A	-6,13	5,80	1,51E-08	ENSG00000136531
ASPA	8,92	6,78	2,28E-08	ENSG00000108381
P2RX1	6,61	6,02	2,84E-08	ENSG00000108405
ILDR2	8,13	5,31	3,07E-08	ENSG00000143195
EGR4	11,59	4,73	3,52E-08	ENSG00000135625
ADGRG2	-10,54	8,35	3,96E-08	ENSG00000173698
GJB2	7,70	5,51	4,64E-08	ENSG00000165474
ERBB3	5,76	5,79	1,20E-07	ENSG00000065361
UGT8	6,93	5,20	1,25E-07	ENSG00000174607
ITGA11	-5,43	8,47	1,71E-07	ENSG00000137809
DGAT2L6	11,58	4,73	2,09E-07	ENSG00000184210
WIPF3	-6,02	5,20	2,09E-07	ENSG00000122574
TRIM54	7,48	5,26	2,36E-07	ENSG00000138100
KRT17	6,65	5,29	3,59E-07	ENSG00000128422
GAP43	7,43	6,79	4,16E-07	ENSG00000172020
LINC01010	11,43	4,57	4,36E-07	ENSG00000236700
FA2H	7,83	5,17	5,15E-07	ENSG00000103089
EGR3	5,24	6,70	5,70E-07	ENSG00000179388
ACTC1	6,31	7,46	6,22E-07	ENSG00000159251
FOXD3	8,73	4,25	7,43E-07	ENSG00000187140
AIF1L	5,09	5,56	7,46E-07	ENSG00000126878
COL9A3	5,08	6,05	9,20E-07	ENSG00000092758
PCOLCE2	-5,78	5,32	1,23E-06	ENSG00000163710
FAM71F1	7,80	4,65	1,30E-06	ENSG00000135248
AC006148.1	-13,78	6,91	1,55E-06	ENSG00000242593
AC005906.2	-7,77	5,46	1,61E-06	ENSG00000256654
LPAR3	-7,64	3,86	1,64E-06	ENSG00000171517
FMO2	6,22	4,40	1,69E-06	ENSG00000094963
MT1H	10,70	3,86	1,73E-06	ENSG00000205358
RTN2	5,98	5,50	2,00E-06	ENSG00000125744
GATM	6,26	7,59	2,39E-06	ENSG00000171766
CLDN1	6,03	4,79	2,61E-06	ENSG00000163347
FGF1	5,26	5,45	2,62E-06	ENSG00000113578
MIR4697HG	6,03	5,78	3,72E-06	ENSG00000280237
CAMK2N1	4,60	6,69	4,53E-06	ENSG00000162545
NPPC	-10,36	3,53	4,53E-06	ENSG00000163273
SHC4	6,20	6,02	4,75E-06	ENSG00000185634
SV2B	10,42	3,59	4,99E-06	ENSG00000185518
KCNA2	6,93	4,20	5,07E-06	ENSG00000177301
IGFBP1	10,52	3,68	5,17E-06	ENSG00000146678
CX3CR1	6,52	4,64	5,24E-06	ENSG00000168329
FSTL5	6,72	5,30	5,25E-06	ENSG00000168843

PRSS12	-7,79	5,63	5,97E-06	ENSG00000164099
C4orf19	-6,41	4,07	5,99E-06	ENSG00000154274
FOXD3-AS1	8,39	3,92	6,00E-06	ENSG00000230798
PRX	5,47	6,39	6,12E-06	ENSG00000105227
COL19A1	7,39	4,29	6,17E-06	ENSG00000082293
KCNK12	6,97	3,83	6,45E-06	ENSG00000184261
AC006487.1	10,08	3,24	6,50E-06	ENSG00000249906
VSIG1	-7,26	3,92	6,62E-06	ENSG00000101842
CHRNA9	10,61	3,78	6,72E-06	ENSG00000174343
GALR1	7,08	4,39	7,34E-06	ENSG00000166573
CLDN19	7,78	4,13	7,36E-06	ENSG00000164007
NTS	-11,56	4,71	7,57E-06	ENSG00000133636
CLDN11	5,23	6,49	8,09E-06	ENSG00000013297
AC093330.1	5,79	5,10	8,10E-06	ENSG00000266844
H19	5,46	9,21	8,16E-06	ENSG00000130600
L1TD1	10,61	3,77	8,80E-06	ENSG00000240563
CTXND1	6,79	4,02	9,41E-06	ENSG00000259417
UCHL1	-4,40	7,65	1,09E-05	ENSG00000154277
AL596330.1	7,38	3,67	1,18E-05	ENSG00000229400
DHH	5,69	5,74	1,21E-05	ENSG00000139549
PNMA5	-10,06	6,97	1,24E-05	ENSG00000198883
CD177	9,75	2,88	1,28E-05	ENSG00000204936
NRXN3	5,94	3,80	1,36E-05	ENSG00000021645
IGSF9	-8,23	8,12	1,40E-05	ENSG00000085552
CES1	5,81	5,85	1,42E-05	ENSG00000198848
PMP2	7,73	3,27	1,53E-05	ENSG00000147588
ARSI	6,43	5,60	1,54E-05	ENSG00000183876
OPALIN	8,00	3,55	1,55E-05	ENSG00000197430
RF01973	5,16	5,53	1,56E-05	ENSG00000274866
AC004637.1	8,82	4,27	1,57E-05	ENSG00000267304
AC133065.2	8,54	4,00	1,60E-05	ENSG00000262488
RF01972	5,25	5,74	1,61E-05	ENSG00000275266
SIM2	-4,24	6,02	1,61E-05	ENSG00000159263
ACPP	6,48	7,38	1,67E-05	ENSG00000014257
SLC9A2	-8,59	5,74	1,73E-05	ENSG00000115616
PCSK1	7,26	4,21	1,75E-05	ENSG00000175426
VANGL2	-4,53	5,84	1,76E-05	ENSG00000162738
BCAS1	6,88	7,27	1,81E-05	ENSG00000064787
NOG	-9,81	3,01	1,86E-05	ENSG00000183691
SDC1	-5,50	8,38	2,00E-05	ENSG00000115884
CNN1	4,65	7,53	2,10E-05	ENSG00000130176
ADGRB1	4,41	6,61	2,18E-05	ENSG00000181790
PRND	6,42	3,30	2,18E-05	ENSG00000171864
IL7R	5,83	6,45	2,26E-05	ENSG00000168685
GUCY1B2	-6,87	3,11	2,47E-05	ENSG00000123201
FOXQ1	-7,60	3,79	2,83E-05	ENSG00000164379
BIRC7	7,50	4,30	2,86E-05	ENSG00000101197
ROBO2	-6,71	4,54	3,04E-05	ENSG00000185008
TFAP2A	6,02	4,62	3,07E-05	ENSG00000137203
LMO3	-6,57	6,19	3,22E-05	ENSG00000048540
PIEZO2	-4,03	7,08	3,48E-05	ENSG00000154864
TMEM132C	-8,83	5,61	3,50E-05	ENSG00000181234
EXTL1	4,95	4,31	3,60E-05	ENSG00000158008
AC021683.2	-4,93	4,35	3,73E-05	ENSG00000267506
ID4	3,90	5,58	3,81E-05	ENSG00000172201
MFAP5	4,76	5,25	3,91E-05	ENSG00000197614
SIM1	-11,05	4,21	3,99E-05	ENSG00000112246
VAT1L	6,72	3,04	4,06E-05	ENSG00000171724
KCNF1	6,07	4,90	4,29E-05	ENSG00000162975
COL22A1	5,96	7,00	4,39E-05	ENSG00000169436
CYP2E1	-4,98	6,35	4,47E-05	ENSG00000130649
SLC6A10P	-11,67	8,05	4,51E-05	ENSG00000214617
NRG1	-4,63	5,63	4,61E-05	ENSG00000157168
MBP	4,14	6,63	4,66E-05	ENSG00000197971

LINC01819	7,36	4,32	4,70E-05	ENSG00000231826
KCNA1	-5,62	3,96	4,94E-05	ENSG00000111262
PITX2	-6,43	4,66	5,43E-05	ENSG00000164093
HCN1	-7,03	4,03	5,47E-05	ENSG00000164588
S100A16	4,52	7,44	5,59E-05	ENSG00000188643
CHL1	5,12	5,38	5,95E-05	ENSG00000134121
KISS1	-10,21	6,50	5,95E-05	ENSG00000170498
SLC44A5	-9,66	5,91	6,01E-05	ENSG00000137968
FAM171B	-3,90	6,67	6,46E-05	ENSG00000144369
AC091152.4	9,12	2,32	6,59E-05	ENSG00000279879
PCP4L1	6,71	3,08	6,61E-05	ENSG00000248485
BMP8B	4,67	6,51	7,08E-05	ENSG00000116985
ENTHD1	7,07	3,50	7,08E-05	ENSG00000176177
LINC00707	6,61	2,93	7,09E-05	ENSG00000238266
OLR1	6,12	4,03	7,16E-05	ENSG00000173391
RTN1	-4,06	5,56	7,33E-05	ENSG00000139970
GRAMD1B	4,48	5,78	7,42E-05	ENSG00000023171
ARNTL2	4,79	4,78	7,78E-05	ENSG00000029153
PTPRD	-4,15	6,02	7,81E-05	ENSG00000153707
AFAP1-AS1	-6,23	6,95	7,83E-05	ENSG00000272620
SEMA3B	4,19	7,56	7,95E-05	ENSG00000012171
ITIH3	4,56	5,97	8,03E-05	ENSG00000162267
KRT23	10,54	3,65	8,38E-05	ENSG00000108244
AC133561.1	-10,24	6,56	8,57E-05	ENSG00000198555
ADAMTS15	4,42	5,52	8,69E-05	ENSG00000166106
FLRT3	6,24	3,53	8,73E-05	ENSG00000125848
FOLH1	-6,42	6,72	9,15E-05	ENSG00000086205
AC004656.1	-3,87	7,19	9,18E-05	ENSG00000260822
CYB5R2	4,34	5,08	9,66E-05	ENSG00000166394
DNAJB5	4,16	5,26	9,66E-05	ENSG00000137094
ALPK3	4,31	5,62	9,70E-05	ENSG00000136383
LINC00482	-7,95	3,36	9,79E-05	ENSG00000185168
CXCL10	4,46	4,34	9,83E-05	ENSG00000169245
ASB2	5,58	3,23	9,84E-05	ENSG00000100628
STK32B	4,71	5,21	9,96E-05	ENSG00000152953
PAPPA2	-9,08	7,61	1,02E-04	ENSG00000116183
STEAP1B	6,08	2,98	1,08E-04	ENSG00000105889
CALHM5	4,34	4,80	1,08E-04	ENSG00000178033
FEZF1-AS1	-10,23	3,41	1,09E-04	ENSG00000230316
NFIA-AS2	5,50	4,31	1,09E-04	ENSG00000237928
OPCML	7,41	2,97	1,09E-04	ENSG00000183715
GRID2	-10,53	3,71	1,12E-04	ENSG00000152208
PLEKHA4	3,64	7,63	1,15E-04	ENSG00000105559
EGFL6	-5,20	6,80	1,19E-04	ENSG00000198759
CCL2	3,58	5,65	1,23E-04	ENSG00000108691
KREMEN2	6,19	3,03	1,23E-04	ENSG00000131650
AC234582.1	-3,64	6,88	1,25E-04	ENSG00000231064
PLEKHB1	4,10	6,70	1,25E-04	ENSG00000021300
GLDC	5,56	3,89	1,28E-04	ENSG00000178445
KIF19	5,73	2,62	1,29E-04	ENSG00000196169
IL1B	9,28	2,49	1,29E-04	ENSG00000125538
FAM83F	6,74	3,18	1,30E-04	ENSG00000133477
LINC00702	4,85	3,94	1,33E-04	ENSG00000233117
CMTM5	4,90	4,63	1,36E-04	ENSG00000166091
CAMK2B	4,95	4,74	1,37E-04	ENSG00000058404
AC008708.2	9,25	2,46	1,39E-04	ENSG00000254187
VWC2	-9,92	3,11	1,39E-04	ENSG00000188730
ADH1C	6,95	2,52	1,41E-04	ENSG00000248144
CNTF	6,38	2,71	1,46E-04	ENSG00000242689
LINC01426	5,42	3,60	1,47E-04	ENSG00000234380
KLHL4	5,61	2,96	1,49E-04	ENSG00000102271
PKIA	5,31	3,77	1,50E-04	ENSG00000171033
FRG1GP	-8,81	5,01	1,54E-04	ENSG00000283023
SV2A	-3,88	5,99	1,55E-04	ENSG00000159164

MYLK	4,00	8,28	1,56E-04	ENSG00000065534
BARX1	-6,46	5,14	1,60E-04	ENSG00000131668
CLCF1	4,49	6,19	1,68E-04	ENSG00000175505
NFASC	4,43	6,27	1,71E-04	ENSG00000163531
MGAT3	4,06	4,69	1,73E-04	ENSG00000128268
LINC00511	4,67	4,64	1,77E-04	ENSG00000227036
MINAR1	-4,08	4,53	1,79E-04	ENSG00000169330
KIAA0319	-7,74	3,89	1,81E-04	ENSG00000137261
SEMA3D	-9,83	6,17	1,82E-04	ENSG00000153993
APOD	4,04	10,29	1,85E-04	ENSG00000189058
AQP4	-9,79	6,67	1,91E-04	ENSG00000171885
EPHA7	-7,04	6,10	1,92E-04	ENSG00000135333
FMO1	4,99	3,71	1,94E-04	ENSG00000010932
PADI2	4,67	4,16	1,95E-04	ENSG00000117115
TSPAN15	4,32	6,54	1,96E-04	ENSG00000099282
KCNMB1	4,25	5,08	1,96E-04	ENSG00000145936
P2RY14	5,29	3,12	1,97E-04	ENSG00000174944
NDNF	-6,40	4,90	1,98E-04	ENSG00000173376
ALDH1A3	4,40	6,85	2,01E-04	ENSG00000184254
FRMPD3	-7,29	3,47	2,03E-04	ENSG00000147234
OASL	4,69	4,27	2,07E-04	ENSG00000135114
RDH5	6,80	2,30	2,07E-04	ENSG00000135437
AC015712.2	4,45	6,13	2,15E-04	ENSG00000259583
CORIN	-4,04	4,52	2,16E-04	ENSG00000145244
AC110772.1	-8,79	4,16	2,21E-04	ENSG00000249742
Z84468.1	-9,18	2,42	2,22E-04	ENSG00000281732
AL355974.2	9,11	2,34	2,27E-04	ENSG00000275830
ATCAY	6,24	3,23	2,29E-04	ENSG00000167654
GALNT5	-5,08	6,33	2,37E-04	ENSG00000136542
HEPH	4,08	5,85	2,45E-04	ENSG00000089472
RPS6KA1	4,28	7,78	2,46E-04	ENSG00000117676
IL17B	5,73	3,52	2,47E-04	ENSG00000127743
UROC1	-7,39	3,48	2,47E-04	ENSG00000159650
SGCA	4,57	3,50	2,51E-04	ENSG00000108823
AC015656.1	8,67	1,91	2,53E-04	ENSG00000279036
CLEC9A	6,13	2,52	2,54E-04	ENSG00000197992
LHPP	3,66	5,86	2,64E-04	ENSG00000107902
PICSAR	8,52	1,75	2,65E-04	ENSG00000275874
LINC01451	7,71	3,21	2,66E-04	ENSG00000279141
HERC2P4	-6,78	5,18	2,75E-04	ENSG00000230267
OPRD1	5,28	4,40	2,75E-04	ENSG00000116329
IRF6	6,68	2,19	2,80E-04	ENSG00000117595
KRT14	5,42	2,38	2,85E-04	ENSG00000186847
SYCE1	-6,56	6,17	2,89E-04	ENSG00000171772
GRIP1	-4,73	4,87	2,91E-04	ENSG00000155974
F5	-7,08	2,53	2,96E-04	ENSG00000198734
S100B	4,78	7,40	2,97E-04	ENSG00000160307
AP001350.2	6,37	2,82	2,99E-04	ENSG00000280010
PAX7	-9,27	5,61	3,03E-04	ENSG00000009709
COLCA2	-4,92	5,16	3,04E-04	ENSG00000214290
OLFM1	-5,50	9,39	3,09E-04	ENSG00000130558
HKDC1	5,86	3,53	3,10E-04	ENSG00000156510
MUC5B	4,85	4,08	3,22E-04	ENSG00000117983
SEZ6L2	4,37	4,29	3,26E-04	ENSG00000174938
CEACAM21	5,47	3,84	3,29E-04	ENSG00000007129
AC104793.1	6,99	2,58	3,33E-04	ENSG00000249568
CHD5	-6,86	6,73	3,35E-04	ENSG00000116254
SMPDL3B	-5,21	3,52	3,37E-04	ENSG00000130768
TAGLN	3,54	9,73	3,39E-04	ENSG00000149591
INMT	3,61	4,86	3,40E-04	ENSG00000241644
NELL2	-6,63	7,07	3,42E-04	ENSG00000184613
MTCL1	-3,46	6,89	3,48E-04	ENSG00000168502
GRIN2A	5,50	3,14	3,50E-04	ENSG00000183454
LINC01141	6,11	2,51	3,50E-04	ENSG00000236963

CSPG4	3,76	8,18	3,52E-04	ENSG00000173546
CXCL11	5,01	3,01	3,54E-04	ENSG00000169248
AC011603.1	6,50	2,89	3,54E-04	ENSG00000257346
FABP5	3,76	4,56	3,57E-04	ENSG00000164687
SPOCD1	4,50	5,76	3,61E-04	ENSG00000134668
NTRK3	4,50	6,32	3,64E-04	ENSG00000140538
BVES	4,60	6,03	3,68E-04	ENSG00000112276
ST6GALNAC2	6,59	2,10	3,68E-04	ENSG00000070731
DIRAS2	5,43	3,64	3,73E-04	ENSG00000165023
TNN	-8,10	7,95	3,77E-04	ENSG00000120332
MAP1B	3,87	7,81	3,80E-04	ENSG00000131711
AC010980.2	-8,13	1,46	3,82E-04	ENSG00000267034
PRDM6	-3,92	4,57	3,84E-04	ENSG00000061455
CALN1	-7,09	5,48	3,84E-04	ENSG00000183166
CELSR2	3,85	5,21	3,87E-04	ENSG00000143126
PLXNC1	-3,32	6,69	3,99E-04	ENSG00000136040
NSG2	-6,95	5,07	4,03E-04	ENSG00000170091
TENM2	4,10	5,52	4,05E-04	ENSG00000145934
MTMR11	-3,38	7,34	4,06E-04	ENSG00000014914
THBS3	-3,35	8,58	4,09E-04	ENSG00000169231
SLC5A12	-7,07	4,04	4,10E-04	ENSG00000148942
APLP1	4,55	4,62	4,11E-04	ENSG00000105290
MOXD1	-5,22	8,12	4,13E-04	ENSG00000079931
SHH	5,69	3,48	4,20E-04	ENSG00000164690
LINC01667	-8,22	4,49	4,28E-04	ENSG00000280081
LY6G6C	-7,33	3,95	4,30E-04	ENSG00000204421
GALNT13	-7,10	6,10	4,31E-04	ENSG00000144278
AC105020.4	4,27	4,70	4,36E-04	ENSG00000260892
C9orf129	-5,69	2,00	4,37E-04	ENSG00000204352
FZD10	-4,80	3,99	4,38E-04	ENSG00000111432
CXCL13	-7,73	8,02	4,38E-04	ENSG00000156234
HS3ST4	6,99	3,74	4,40E-04	ENSG00000182601
LARGE2	-6,85	2,33	4,45E-04	ENSG00000165905
GIPC3	4,01	4,43	4,50E-04	ENSG00000179855
COLCA1	-4,14	4,90	4,50E-04	ENSG00000196167
POU2AF1	-5,39	3,73	4,53E-04	ENSG00000110777
PHF2P2	-9,05	2,30	4,54E-04	ENSG00000226057
NES	4,12	8,13	4,57E-04	ENSG00000132688
GJB1	8,60	1,85	4,58E-04	ENSG00000169562
ANO1-AS1	-4,40	6,81	4,61E-04	ENSG00000254902
GALNT3	-3,86	5,15	4,62E-04	ENSG00000115339
DRAXIN	5,04	4,04	4,62E-04	ENSG00000162490
LGI1	8,11	1,35	4,63E-04	ENSG00000108231
LVRN	-8,80	7,98	4,67E-04	ENSG00000172901
SLC30A8	-8,24	3,62	4,70E-04	ENSG00000164756
CXCL12	3,27	7,26	4,81E-04	ENSG00000107562
NKAIN4	6,14	3,54	4,84E-04	ENSG00000101198
LINC01629	6,29	3,29	4,91E-04	ENSG00000258602
GATA6	-3,56	5,62	4,91E-04	ENSG00000141448
NIM1K	5,38	3,56	4,91E-04	ENSG00000177453
MIR1-1HG-AS1	5,29	2,18	4,99E-04	ENSG00000174403
AC008522.1	3,72	5,13	5,14E-04	ENSG00000279232
IL17RE	4,29	4,64	5,17E-04	ENSG00000163701
BOK	3,47	5,50	5,20E-04	ENSG00000176720
CACNA1G	-4,17	4,08	5,23E-04	ENSG00000006283
EMID1	-3,94	4,87	5,26E-04	ENSG00000186998
GPR22	-9,40	2,62	5,33E-04	ENSG00000172209
KREMEN1	-3,42	7,00	5,42E-04	ENSG00000183762
APOL4	4,20	3,98	5,45E-04	ENSG00000100336
CELF2	3,41	6,65	5,46E-04	ENSG00000048740
KANK4	5,30	3,46	5,47E-04	ENSG00000132854
AL354861.3	5,33	2,90	5,57E-04	ENSG00000268926
GGT4P	-9,14	2,38	5,68E-04	ENSG00000280208
SOX2-OT	4,67	3,82	5,75E-04	ENSG00000242808

UMODL1	-4,86	2,97	5,82E-04	ENSG00000177398
IL31RA	5,07	2,95	5,84E-04	ENSG00000164509
AC125611.4	-6,17	5,97	5,98E-04	ENSG00000258334
CHI3L1	3,83	5,42	6,04E-04	ENSG00000133048
PRH2	7,82	5,54	6,05E-04	ENSG00000134551
C3	3,44	7,76	6,05E-04	ENSG00000125730
SCN5A	-6,25	3,44	6,09E-04	ENSG00000183873
AZGP1	5,12	2,46	6,09E-04	ENSG00000160862
HOTTIP	-9,51	2,73	6,13E-04	ENSG00000243766
FADS3	3,48	7,89	6,16E-04	ENSG00000221968
ADGRA1	7,40	3,71	6,16E-04	ENSG00000197177
SEMA3G	3,34	5,02	6,18E-04	ENSG00000010319
BAALC	5,38	3,07	6,21E-04	ENSG00000164929
GAS7	3,55	7,70	6,23E-04	ENSG00000007237
ADAMTS17	-3,81	5,12	6,41E-04	ENSG00000140470
ADAMTS3	-3,85	4,30	6,44E-04	ENSG00000156140
AC092834.1	-8,49	1,77	6,46E-04	ENSG00000249453
IFI27	3,24	6,93	6,54E-04	ENSG00000165949
MAGEA12	-9,46	2,68	6,60E-04	ENSG00000213401
OXTR	4,20	5,12	6,60E-04	ENSG00000180914
CYP3A5	-6,08	5,48	6,61E-04	ENSG00000106258
APOBEC3C	3,39	4,98	6,62E-04	ENSG00000244509
HORMAD1	-8,00	3,39	6,73E-04	ENSG00000143452
RNF212	-4,43	5,08	6,84E-04	ENSG00000178222
AC246817.2	-8,61	1,89	6,93E-04	ENSG00000254319
GNG11	3,23	6,20	7,03E-04	ENSG00000127920
STUM	4,20	3,59	7,10E-04	ENSG00000203685
AKAP5	5,00	4,04	7,11E-04	ENSG00000179841
USH1C	-7,69	5,25	7,15E-04	ENSG00000006611
CSMD1	-5,38	3,60	7,16E-04	ENSG00000183117
GABRD	5,83	7,18	7,18E-04	ENSG00000187730
AC092958.1	6,64	2,17	7,35E-04	ENSG00000239922
CNIH3	3,96	5,83	7,39E-04	ENSG00000143786
CACNA1E	-6,70	7,04	7,46E-04	ENSG00000198216
HS6ST2	-5,42	4,15	7,51E-04	ENSG00000171004
HTN1	7,72	4,91	7,53E-04	ENSG00000126550
ANO1	-3,97	8,67	7,55E-04	ENSG00000131620
TFAP2A-AS1	8,45	1,72	7,64E-04	ENSG00000229950
TTN	-3,79	8,04	7,68E-04	ENSG00000155657
AGTR1	-5,28	4,50	7,74E-04	ENSG00000144891
PPM1N	4,46	3,94	7,78E-04	ENSG00000213889
HMCN1	-3,65	6,73	7,85E-04	ENSG00000143341
EBF2	3,52	4,57	7,99E-04	ENSG00000221818
CXCL9	4,35	4,80	8,09E-04	ENSG00000138755
HLA-DRA	3,20	8,64	8,38E-04	ENSG00000204287
OTX1	-7,20	4,04	8,39E-04	ENSG00000115507
MAGEC2	-9,22	2,46	8,58E-04	ENSG00000046774
FER1L4	-3,21	5,71	8,60E-04	ENSG00000088340
AC007686.4	5,93	2,36	8,64E-04	ENSG00000285966
ITGA7	3,06	7,49	8,75E-04	ENSG00000135424
GREM2	-6,59	5,49	8,79E-04	ENSG00000180875
KRT15	7,78	5,66	8,86E-04	ENSG00000171346
RELN	5,11	2,11	8,86E-04	ENSG00000189056
GFOD1	3,12	5,75	8,99E-04	ENSG00000145990
MEDAG	3,33	4,90	9,03E-04	ENSG00000102802
MAG	5,80	2,22	9,23E-04	ENSG00000105695
WDR72	6,02	2,44	9,25E-04	ENSG00000166415
NRK	-3,93	5,21	9,26E-04	ENSG00000123572
LGALS3BP	3,21	7,99	9,27E-04	ENSG00000108679
ANKRD13B	-3,88	7,54	9,30E-04	ENSG00000198720
AL354919.2	5,65	3,45	9,31E-04	ENSG00000254545
GOLGA8B	-4,31	10,17	9,32E-04	ENSG00000215252
RAMP1	4,69	4,10	9,36E-04	ENSG00000132329
AREG	4,79	3,37	9,49E-04	ENSG00000109321

AL512288.1	-4,96	3,03	9,53E-04	ENSG00000227050
COL25A1	-5,69	5,68	9,54E-04	ENSG00000188517
EEF1A2	4,65	4,46	9,56E-04	ENSG00000101210
GDF6	-6,28	5,19	9,63E-04	ENSG00000156466
LAMC2	-6,34	4,38	9,67E-04	ENSG00000058085
PNMA3	-5,33	4,50	9,69E-04	ENSG00000183837
KCNH5	-8,88	2,14	9,71E-04	ENSG00000140015
DYNC1I1	-3,29	5,08	9,77E-04	ENSG00000158560
HACD1	5,29	4,12	1,02E-03	ENSG00000165996
NPTXR	4,04	5,87	1,02E-03	ENSG00000221890
ACKR2	-6,12	4,81	1,02E-03	ENSG00000144648
EFCAB1	-5,98	2,24	1,03E-03	ENSG00000034239
E2F1	4,25	6,66	1,04E-03	ENSG00000101412
CILP2	-5,05	7,35	1,05E-03	ENSG00000160161
BACH2	-3,51	4,38	1,05E-03	ENSG00000112182
SERPINI1	-4,82	6,21	1,05E-03	ENSG00000163536
LONRF2	4,50	3,93	1,06E-03	ENSG00000170500
LINC00518	6,33	1,96	1,06E-03	ENSG00000183674
TSPAN10	4,49	4,14	1,06E-03	ENSG00000182612
AP000924.1	8,59	1,86	1,07E-03	ENSG00000254416
LMOD1	3,18	6,53	1,07E-03	ENSG00000163431
MYH11	3,06	8,62	1,07E-03	ENSG00000133392
AC020656.1	3,37	4,58	1,07E-03	ENSG00000257764
CLCN4	-3,87	4,87	1,09E-03	ENSG00000073464
OGN	3,20	5,24	1,10E-03	ENSG00000106809
AC025370.1	6,19	1,66	1,11E-03	ENSG00000253851
UNCX	-8,63	5,03	1,12E-03	ENSG00000164853
AC003035.2	6,44	2,07	1,14E-03	ENSG00000233535
BMP3	-6,62	3,85	1,15E-03	ENSG00000152785
AVPR1A	-3,87	5,94	1,16E-03	ENSG00000166148
TH	6,03	2,52	1,16E-03	ENSG00000180176
CACNA2D1	3,92	5,42	1,17E-03	ENSG00000153956
KCNN4	4,02	5,30	1,17E-03	ENSG00000104783
ADAMTS4	3,30	6,92	1,18E-03	ENSG00000158859
EYA4	-5,62	5,64	1,18E-03	ENSG00000112319
GABRA2	-5,59	6,13	1,19E-03	ENSG00000151834
XRCC4	3,76	4,92	1,22E-03	ENSG00000152422
FAM180A	-5,17	5,67	1,22E-03	ENSG00000189320
DIRAS3	4,07	4,97	1,22E-03	ENSG00000162595
DTNA	4,00	3,72	1,25E-03	ENSG00000134769
CLEC18C	5,98	2,47	1,25E-03	ENSG00000157335
KRT8P23	-9,05	2,29	1,27E-03	ENSG00000259565
GPR63	-4,17	4,35	1,28E-03	ENSG00000112218
BHLHE41	3,75	6,02	1,28E-03	ENSG00000123095
AC083841.1	7,83	1,14	1,29E-03	ENSG00000253196
HMCN2	-4,92	6,83	1,29E-03	ENSG00000148357
LMNTD1	8,42	1,71	1,30E-03	ENSG00000152936
SOX2	4,35	5,02	1,31E-03	ENSG00000181449
ADGRG5	5,29	4,00	1,31E-03	ENSG00000159618
SLC47A1	-6,25	5,39	1,31E-03	ENSG00000142494
VEPH1	-4,88	4,69	1,32E-03	ENSG00000197415
CYGB	3,13	5,52	1,32E-03	ENSG00000161544
BFSP1	-4,30	5,01	1,33E-03	ENSG00000125864
ETHE1	3,34	5,07	1,33E-03	ENSG00000105755
SH2D4A	-3,70	4,27	1,33E-03	ENSG00000104611
GSTM1	6,05	3,07	1,33E-03	ENSG00000134184
AC079801.1	-8,84	2,10	1,35E-03	ENSG00000279669
PCDH8	5,36	2,24	1,35E-03	ENSG00000136099
C2CD4C	-5,79	7,52	1,37E-03	ENSG00000183186
IGLV1-51	-4,56	2,53	1,38E-03	ENSG00000211644
VEGFA	-2,97	8,15	1,39E-03	ENSG00000112715
HOXC12	-5,61	2,67	1,41E-03	ENSG00000123407
AL009181.1	5,37	3,22	1,42E-03	ENSG00000233427
KRT7	7,10	2,59	1,42E-03	ENSG00000135480

FRG1FP	-7,60	5,63	1,42E-03	ENSG00000283047
PLXNB3	3,65	4,24	1,43E-03	ENSG00000198753
AC068594.1	4,96	2,28	1,44E-03	ENSG00000263718
AL136131.3	-4,10	3,62	1,44E-03	ENSG00000272114
IDH2	3,15	5,93	1,44E-03	ENSG00000182054
FGFR3	5,22	7,21	1,45E-03	ENSG00000068078
AC022075.1	5,04	2,06	1,48E-03	ENSG00000245648
UST	-4,36	5,70	1,48E-03	ENSG00000111962
PTPRU	-3,54	6,98	1,52E-03	ENSG00000060656
HLA-DPA1	3,26	8,44	1,53E-03	ENSG00000231389
GNG4	4,53	2,17	1,54E-03	ENSG00000168243
GPC4	-4,46	6,20	1,55E-03	ENSG00000076716
GAPT	4,99	3,20	1,56E-03	ENSG00000175857
ADAMTS6	-4,66	6,88	1,58E-03	ENSG00000049192
NLRP3P1	-6,43	2,57	1,59E-03	ENSG00000277883
AL512329.2	-5,32	2,10	1,59E-03	ENSG00000272279
SYT12	5,29	3,97	1,61E-03	ENSG00000173227
KCNH2	4,36	3,28	1,61E-03	ENSG00000055118
AC012354.1	-8,68	1,96	1,63E-03	ENSG00000225156
CTGF	2,88	8,32	1,65E-03	ENSG00000118523
PCDH10	-6,05	5,45	1,65E-03	ENSG00000138650
CA9	-7,37	4,62	1,65E-03	ENSG00000107159
SIGIRR	3,61	7,01	1,66E-03	ENSG00000185187
PRPH	-5,47	6,46	1,66E-03	ENSG00000135406
EMILIN3	-3,88	7,30	1,66E-03	ENSG00000183798
RBM47	3,73	4,83	1,66E-03	ENSG00000163694
ATP10B	8,08	1,21	1,66E-03	ENSG00000118322
SEC14L4	5,96	1,54	1,67E-03	ENSG00000133488
LY6H	4,33	2,62	1,67E-03	ENSG00000176956
AKAP12	3,69	7,89	1,67E-03	ENSG00000131016
NAPSB	3,95	4,33	1,68E-03	ENSG00000131401
LINC01914	7,46	0,77	1,69E-03	ENSG00000234362
RGMB	3,16	5,69	1,71E-03	ENSG00000174136
CERS1	4,41	4,02	1,71E-03	ENSG00000223802
AC010980.1	-5,82	1,40	1,72E-03	ENSG00000237732
SLC9A3-AS1	-3,18	5,78	1,72E-03	ENSG00000225138
PLAGL1	-2,92	8,36	1,72E-03	ENSG00000118495
IGSF9B	4,33	3,66	1,74E-03	ENSG00000080854
ASPHD1	3,85	4,23	1,74E-03	ENSG00000174939
FUT8	3,10	5,56	1,75E-03	ENSG00000033170
CDHR1	5,43	5,19	1,75E-03	ENSG00000148600
GATA6-AS1	-3,85	4,29	1,76E-03	ENSG00000266010
CADPS	4,49	2,67	1,76E-03	ENSG00000163618
HOMER1	3,52	5,44	1,77E-03	ENSG00000152413
MAGEB17	-5,81	2,09	1,81E-03	ENSG00000182798
LINC02434	7,81	1,13	1,81E-03	ENSG00000248370
CFD	3,66	5,30	1,84E-03	ENSG00000197766
LAMP5	-4,85	6,51	1,86E-03	ENSG00000125869
PITPNM2	-3,16	6,39	1,86E-03	ENSG00000090975
HS6ST3	-7,80	1,18	1,87E-03	ENSG00000185352
VSTM2L	5,26	1,71	1,87E-03	ENSG00000132821
SORT1	3,09	6,32	1,87E-03	ENSG00000134243
PDE10A	-3,21	5,30	1,90E-03	ENSG00000112541
HOXB9	6,29	1,94	1,91E-03	ENSG00000170689
OR7E14P	4,99	2,02	1,92E-03	ENSG00000184669
RAB9B	4,40	2,49	1,93E-03	ENSG00000123570
AHRR	-3,98	4,05	1,93E-03	ENSG00000063438
CCKAR	-7,78	1,15	1,94E-03	ENSG00000163394
VIT	5,07	2,10	1,94E-03	ENSG00000205221
ZNF704	-2,95	6,71	1,99E-03	ENSG00000164684
S100A1	3,45	4,39	2,00E-03	ENSG00000160678
ZNF350-AS1	-4,94	1,34	2,00E-03	ENSG00000269235
MEGF11	-5,51	2,26	2,05E-03	ENSG00000157890
LGR5	-4,24	5,40	2,07E-03	ENSG00000139292

CEACAM1	-5,94	5,83	2,09E-03	ENSG00000079385
GPR153	-2,86	6,87	2,09E-03	ENSG00000158292
NLGN1	4,37	3,41	2,10E-03	ENSG00000169760
IGSF1	4,29	4,42	2,11E-03	ENSG00000147255
SERTM1	-7,31	3,90	2,12E-03	ENSG00000180440
AC078993.1	-4,96	3,01	2,13E-03	ENSG00000238178
VGLL3	3,29	4,54	2,15E-03	ENSG00000206538
MAML3	3,81	3,90	2,19E-03	ENSG00000196782
HMGA2	-3,96	3,67	2,19E-03	ENSG00000149948
CLDN7	3,78	4,92	2,20E-03	ENSG00000181885
GRM8	-5,03	4,84	2,20E-03	ENSG00000179603
TARID	-6,12	6,39	2,22E-03	ENSG00000227954
FAM131B	4,56	3,25	2,23E-03	ENSG00000159784
TMEM229B	3,97	3,24	2,26E-03	ENSG00000198133
LRRC32	2,81	6,52	2,27E-03	ENSG00000137507
SORL1	3,40	5,40	2,27E-03	ENSG00000137642
SEMA6B	2,78	5,58	2,31E-03	ENSG00000167680
TFCP2L1	-4,80	5,11	2,32E-03	ENSG00000115112
HAPLN1	-5,66	4,86	2,34E-03	ENSG00000145681
AL033530.1	-8,34	1,65	2,36E-03	ENSG00000285407
TAC1	4,86	1,89	2,36E-03	ENSG00000006128
CTPS1	-2,78	6,82	2,37E-03	ENSG00000171793
AC011893.1	7,92	1,25	2,38E-03	ENSG00000226806
AC002401.4	7,61	0,96	2,39E-03	ENSG00000276851
COL8A1	3,54	7,60	2,39E-03	ENSG00000144810
HIF3A	-3,18	4,84	2,40E-03	ENSG00000124440
PRSS30P	-4,63	3,69	2,41E-03	ENSG00000172460
RAPGEF5	3,03	4,97	2,41E-03	ENSG00000136237
FPR3	3,60	3,50	2,43E-03	ENSG00000187474
DRD2	-8,48	1,76	2,43E-03	ENSG00000149295
EBF4	-2,84	5,40	2,44E-03	ENSG00000088881
FGF19	-6,61	6,38	2,46E-03	ENSG00000162344
MCOLN3	-3,86	3,68	2,49E-03	ENSG00000055732
ABCC3	3,23	5,73	2,51E-03	ENSG00000108846
MEX3A	-4,91	5,39	2,52E-03	ENSG00000254726
KBTBD12	-3,38	4,28	2,52E-03	ENSG00000187715
GAL3ST1	4,63	3,14	2,53E-03	ENSG00000128242
KIAA1549	-3,42	5,07	2,54E-03	ENSG00000122778
NYAP2	-8,27	1,59	2,57E-03	ENSG00000144460
SGK1	3,26	7,58	2,58E-03	ENSG00000118515
SLC16A14	3,50	3,89	2,58E-03	ENSG00000163053
EPHX3	5,43	2,48	2,58E-03	ENSG00000105131
CRTAC1	4,12	2,80	2,62E-03	ENSG00000095713
ISM2	-7,52	0,92	2,63E-03	ENSG00000100593
NUAK2	4,33	2,52	2,63E-03	ENSG00000163545
KLHL14	-5,41	4,44	2,65E-03	ENSG00000197705
UBASH3B	3,68	3,90	2,66E-03	ENSG00000154127
AF165147.1	-6,53	2,02	2,69E-03	ENSG00000232855
LYZ	2,85	5,19	2,70E-03	ENSG00000090382
HLA-G	-4,13	3,24	2,71E-03	ENSG00000204632
MAATS1	-3,24	4,70	2,73E-03	ENSG00000183833
HERC2P3	-3,79	6,57	2,73E-03	ENSG00000180229
SORCS2	-4,02	7,11	2,73E-03	ENSG00000184985
CFL2	3,37	7,55	2,76E-03	ENSG00000165410
B3GAT1	4,19	4,08	2,77E-03	ENSG00000109956
GNAO1	-5,62	6,67	2,78E-03	ENSG00000087258
LYPD8	-4,76	1,61	2,79E-03	ENSG00000259823
ESRP1	-7,54	5,21	2,80E-03	ENSG00000104413
KHDRBS2	-6,08	2,31	2,81E-03	ENSG00000112232
CLEC18B	3,44	4,22	2,81E-03	ENSG00000140839
CHST6	3,60	3,43	2,82E-03	ENSG00000183196
AC243547.1	-4,91	5,48	2,83E-03	ENSG00000244619
HPN-AS1	5,80	1,48	2,84E-03	ENSG00000227392
ZDHHC8P1	5,89	4,04	2,84E-03	ENSG00000133519

SIX3	-7,52	5,19	2,87E-03	ENSG00000138083
RPSAP52	-7,13	0,61	2,88E-03	ENSG00000241749
ACTN2	-5,50	4,01	2,88E-03	ENSG00000077522
AC021683.1	-3,68	3,93	2,89E-03	ENSG00000267466
AP001180.4	-4,96	1,78	2,89E-03	ENSG00000264843
CPAMD8	-3,36	5,57	2,89E-03	ENSG00000160111
AL445253.1	-4,57	3,76	2,92E-03	ENSG00000285873
AC055854.1	4,20	2,13	2,92E-03	ENSG00000253125
SYPL2	4,46	3,29	2,92E-03	ENSG00000143028
METTL7B	3,40	4,05	2,93E-03	ENSG00000170439
RDH10	3,20	5,31	2,94E-03	ENSG00000121039
THBS1	2,84	8,38	3,01E-03	ENSG00000137801
PTGER3	-3,48	5,61	3,07E-03	ENSG00000050628
C2orf66	-4,72	4,86	3,09E-03	ENSG00000187944
BEX1	6,64	3,02	3,10E-03	ENSG00000133169
MEG3	-3,02	9,69	3,12E-03	ENSG00000214548
KRT7-AS	6,39	1,88	3,15E-03	ENSG00000257671
BIRC3	2,98	5,85	3,16E-03	ENSG00000023445
CXADR	3,95	2,86	3,21E-03	ENSG00000154639
CST2	4,07	2,36	3,22E-03	ENSG00000170369
ACVR1C	-7,14	3,51	3,22E-03	ENSG00000123612
PRPH2	5,28	2,34	3,22E-03	ENSG00000112619
AC009065.5	-5,12	1,48	3,23E-03	ENSG00000261123
VSTM2A	-7,87	1,24	3,23E-03	ENSG00000170419
PTX3	-5,71	7,03	3,24E-03	ENSG00000163661
AC092969.1	-7,45	0,88	3,29E-03	ENSG00000240086
MEIS1	-2,73	5,29	3,33E-03	ENSG00000143995
DAG1	3,21	8,36	3,34E-03	ENSG00000173402
PRUNE2	3,54	7,46	3,35E-03	ENSG00000106772
ACSM5	3,81	4,06	3,35E-03	ENSG00000183549
TRPM8	4,67	2,11	3,37E-03	ENSG00000144481
WNT4	-4,63	7,48	3,38E-03	ENSG00000162552
KLRB1	4,32	2,06	3,39E-03	ENSG00000111796
AKR1B10	5,65	1,34	3,40E-03	ENSG00000198074
CPB1	-8,03	1,38	3,40E-03	ENSG00000153002
ANKRD65	3,50	3,86	3,41E-03	ENSG00000235098
GALNTL6	-4,00	4,02	3,42E-03	ENSG00000174473
NPNT	-5,37	8,37	3,43E-03	ENSG00000168743
NFATC1	-3,54	7,17	3,43E-03	ENSG00000131196
SSPN	3,15	5,06	3,45E-03	ENSG00000123096
FOXS1	3,09	4,66	3,45E-03	ENSG00000179772
PKHD1L1	-6,84	4,49	3,50E-03	ENSG00000205038
INSC	4,73	2,66	3,51E-03	ENSG00000188487
DUX4L37	-6,46	1,96	3,52E-03	ENSG00000283020
NEGR1	-3,95	7,21	3,53E-03	ENSG00000172260
MUC1	-2,85	5,69	3,53E-03	ENSG00000185499
APOE	3,01	6,59	3,55E-03	ENSG00000130203
CD1D	3,84	2,95	3,55E-03	ENSG00000158473
PPM1E	3,85	3,62	3,57E-03	ENSG00000175175
APOC1	4,63	2,69	3,58E-03	ENSG00000130208
AC015802.1	4,31	1,72	3,59E-03	ENSG00000267078
B3GALNT1	-2,81	5,39	3,62E-03	ENSG00000169255
WNT5A	-3,32	4,24	3,64E-03	ENSG00000114251
OCSTAMP	6,10	1,78	3,64E-03	ENSG00000149635
DHRS3	2,79	6,02	3,67E-03	ENSG00000162496
TLR7	3,81	3,30	3,67E-03	ENSG00000196664
GOLGA6L9	-3,57	5,03	3,68E-03	ENSG00000197978
CCDC144B	-4,29	1,77	3,70E-03	ENSG00000154874
SCML2	-4,63	2,61	3,70E-03	ENSG00000102098
AL024497.2	-6,51	3,46	3,71E-03	ENSG00000234567
ADAP1	3,48	3,63	3,72E-03	ENSG00000105963
XKR8	-3,34	5,96	3,73E-03	ENSG00000158156
KLHL30	4,59	2,13	3,75E-03	ENSG00000168427
LINC02452	4,83	2,29	3,78E-03	ENSG00000283422

OCLN	4,67	1,67	3,80E-03	ENSG00000197822
HS3ST1	-3,12	5,38	3,80E-03	ENSG00000002587
TIMP3	3,16	9,58	3,80E-03	ENSG00000100234
AL035446.1	-5,43	1,74	3,86E-03	ENSG00000234147
PCSK7	2,71	9,36	3,86E-03	ENSG00000160613
PDK3	-3,02	4,74	3,86E-03	ENSG00000067992
COL9A1	-5,07	5,01	3,86E-03	ENSG00000112280
RALGPS2	3,24	3,91	3,87E-03	ENSG00000116191
RASSF2	2,71	5,62	3,88E-03	ENSG00000101265
BANCR	5,62	1,24	3,89E-03	ENSG00000278910
CNN2	2,58	6,46	3,89E-03	ENSG00000064666
C5AR2	-4,99	5,56	3,91E-03	ENSG00000134830
RPRM	-6,00	3,25	3,92E-03	ENSG00000177519
AC084816.1	-6,59	3,50	3,94E-03	ENSG00000256995
PGAP1	-2,91	6,59	3,97E-03	ENSG00000197121
AC254629.1	4,37	2,38	3,98E-03	ENSG00000274993
AC009902.2	3,58	3,07	4,08E-03	ENSG00000254027
PTH2R	-5,77	3,52	4,09E-03	ENSG00000144407
OR51E1	4,21	1,66	4,10E-03	ENSG00000180785
PLIN4	3,83	3,54	4,12E-03	ENSG00000167676
SLFN5	2,89	6,18	4,14E-03	ENSG00000166750
STAB2	-3,76	2,70	4,14E-03	ENSG00000136011
AC068700.1	7,05	0,44	4,14E-03	ENSG00000260398
SLC27A6	-6,19	5,39	4,16E-03	ENSG00000113396
GRPR	-5,85	2,54	4,19E-03	ENSG00000126010
DCHS2	-4,52	3,72	4,21E-03	ENSG00000197410
PHKA2-AS1	-2,53	5,90	4,23E-03	ENSG00000237836
ASS1	-3,74	8,75	4,23E-03	ENSG00000130707
CSRP1	2,86	9,14	4,24E-03	ENSG00000159176
PGR	-4,60	4,62	4,24E-03	ENSG00000082175
LY86	3,65	3,44	4,27E-03	ENSG00000112799
ASIC2	-4,93	1,31	4,27E-03	ENSG00000108684
AL109615.3	-4,57	2,15	4,27E-03	ENSG00000237686
MAMDC4	-3,01	6,88	4,32E-03	ENSG00000177943
LAMC3	-3,18	5,29	4,33E-03	ENSG00000050555
IGF2BP1	5,21	2,46	4,34E-03	ENSG00000159217
FCER1A	3,55	2,88	4,36E-03	ENSG00000179639
ARHGAP19	2,96	5,35	4,36E-03	ENSG00000213390
LINCR-0001	4,61	4,21	4,36E-03	ENSG00000253641
OPN3	3,39	4,15	4,38E-03	ENSG00000054277
LINC02285	5,54	1,16	4,41E-03	ENSG00000259004
ARFGEF3	-4,08	4,76	4,42E-03	ENSG00000112379
GSG1L	-6,29	4,76	4,45E-03	ENSG00000169181
AC022706.1	4,49	3,39	4,46E-03	ENSG00000267364
SMTN	2,71	7,56	4,48E-03	ENSG00000183963
SPARC	2,68	11,40	4,51E-03	ENSG00000113140
PLEKHN1	-3,36	3,63	4,52E-03	ENSG00000187583
ZBTB42	3,42	3,88	4,53E-03	ENSG00000179627
PHKA2	-2,58	7,07	4,54E-03	ENSG00000044446
SEMA3A	3,98	2,80	4,54E-03	ENSG00000075213
HLA-DRB5	2,83	5,77	4,55E-03	ENSG00000198502
C1orf226	-3,19	4,01	4,56E-03	ENSG00000239887
VWCE	2,90	4,53	4,57E-03	ENSG00000167992
SYN3	4,30	1,68	4,59E-03	ENSG00000185666
C1orf61	4,11	3,01	4,60E-03	ENSG00000125462
TRDC	-5,41	4,66	4,60E-03	ENSG00000211829
SLC24A4	-4,45	3,91	4,63E-03	ENSG00000140090
C8orf34	-7,43	0,87	4,64E-03	ENSG00000165084
F2RL2	-3,05	5,19	4,69E-03	ENSG00000164220
KLHL23	3,36	5,87	4,73E-03	ENSG00000213160
RNF133	-7,75	1,13	4,76E-03	ENSG00000188050
FNDC10	3,31	3,60	4,76E-03	ENSG00000228594
ADAMTSL4	-2,81	5,65	4,77E-03	ENSG00000143382
RPL23AP25	-7,25	0,71	4,78E-03	ENSG00000233084

AC073133.1	-6,87	0,40	4,79E-03	ENSG00000228569
XDH	-5,02	1,82	4,81E-03	ENSG00000158125
SLC6A4	-5,21	4,58	4,82E-03	ENSG00000108576
AC005220.1	4,51	0,94	4,84E-03	ENSG00000236352
GPM6A	-5,57	5,10	4,84E-03	ENSG00000150625
RASGRF1	5,05	4,19	4,85E-03	ENSG00000058335
AL590652.1	-4,51	0,98	4,87E-03	ENSG00000227885
HHEX	3,11	4,02	4,88E-03	ENSG00000152804
NKD2	-3,45	6,72	4,89E-03	ENSG00000145506
HMOX1	2,91	4,54	4,94E-03	ENSG00000100292
INHBA	-2,65	6,47	4,94E-03	ENSG00000122641
FEZF1	-7,70	1,09	4,95E-03	ENSG00000128610
SCN9A	3,99	4,02	4,96E-03	ENSG00000169432
LAMP5-AS1	-6,18	3,10	4,96E-03	ENSG00000225988
VIPR2	4,23	5,11	4,99E-03	ENSG00000106018
AL157955.1	5,32	0,96	4,99E-03	ENSG00000258407
AC016735.1	6,90	0,32	5,00E-03	ENSG00000224739
AL136379.1	-5,91	1,47	5,00E-03	ENSG00000276255
PCSK2	-5,49	2,38	5,04E-03	ENSG00000125851
CAMKK1	2,82	6,67	5,05E-03	ENSG00000004660
AC008074.2	3,93	2,97	5,06E-03	ENSG00000260101
SRD5A2	-5,92	1,47	5,07E-03	ENSG00000277893
MCAM	2,71	8,13	5,09E-03	ENSG00000076706
FILIP1L	2,47	5,75	5,14E-03	ENSG00000168386
IRS4	-7,26	3,71	5,17E-03	ENSG00000133124
UNC13C	-5,01	2,79	5,21E-03	ENSG00000137766
PLXNA3	-2,84	7,67	5,25E-03	ENSG00000130827
GLCCI1	-2,64	5,14	5,26E-03	ENSG00000106415
TPX2	-2,72	4,85	5,28E-03	ENSG00000088325
RPP25	3,14	4,19	5,30E-03	ENSG00000178718
RASSF10	-5,45	2,19	5,30E-03	ENSG00000189431
KIF5C	-4,17	5,15	5,34E-03	ENSG00000168280
SLC9B2	-3,13	4,86	5,34E-03	ENSG00000164038
RGS1	2,94	7,05	5,36E-03	ENSG00000090104
DOC2B	-3,24	6,73	5,37E-03	ENSG00000272636
GPR137B	2,87	4,93	5,38E-03	ENSG00000077585
SLC5A8	-7,22	3,67	5,39E-03	ENSG00000256870
GIMAP4	2,62	5,16	5,39E-03	ENSG00000133574
KRT81	6,85	3,24	5,42E-03	ENSG00000205426
SPINT1	3,56	4,18	5,45E-03	ENSG00000166145
DMRT2	-5,28	5,22	5,46E-03	ENSG00000173253
A1CF	-6,29	1,80	5,46E-03	ENSG00000148584
HAMP	4,72	1,81	5,46E-03	ENSG00000105697
SLFNL1	-3,88	2,64	5,46E-03	ENSG00000171790
PROX1	-4,83	4,02	5,49E-03	ENSG00000117707
AC005392.3	5,28	0,91	5,50E-03	ENSG00000272396
PBX3	-2,92	7,13	5,51E-03	ENSG00000167081
TLR10	4,18	1,92	5,52E-03	ENSG00000174123
AMZ1	4,48	2,39	5,52E-03	ENSG00000174945
GFRA3	4,04	2,53	5,54E-03	ENSG00000146013
NAP1L3	3,39	3,99	5,54E-03	ENSG00000186310
MAPK4	5,79	3,03	5,57E-03	ENSG00000141639
VASN	-2,92	7,11	5,58E-03	ENSG00000168140
LINC02091	-7,13	0,60	5,59E-03	ENSG00000273172
CST4	6,05	3,34	5,65E-03	ENSG00000101441
AL449403.1	6,21	3,09	5,69E-03	ENSG00000237212
CTB-178M22.2	-6,18	1,68	5,72E-03	ENSG00000253978
INTU	-2,79	6,00	5,77E-03	ENSG00000164066
NOS1AP	-5,15	2,05	5,80E-03	ENSG00000198929
ADCY2	-3,39	5,90	5,81E-03	ENSG00000078295
P2RY13	3,66	3,33	5,82E-03	ENSG00000181631
HLA-DQA1	2,60	5,91	5,85E-03	ENSG00000196735
CHTF18	-2,85	6,54	5,87E-03	ENSG00000127586
AC007639.1	7,18	0,60	5,89E-03	ENSG00000263680

CNKSR1	-4,26	5,00	5,90E-03	ENSG00000142675
PCSK1N	4,09	2,12	5,90E-03	ENSG00000102109
STEAP4	2,74	5,18	5,91E-03	ENSG00000127954
AC093607.1	-6,73	0,30	5,92E-03	ENSG00000250137
AC090921.1	-4,74	1,86	5,95E-03	ENSG00000214803
ISL1	-6,46	4,11	5,96E-03	ENSG00000016082
PTH1R	-2,88	5,53	5,97E-03	ENSG00000160801
CCDC141	-5,42	3,39	5,98E-03	ENSG00000163492
PHF24	4,35	2,59	5,98E-03	ENSG00000122733
AC112721.2	5,43	1,16	6,03E-03	ENSG00000222032
SPTLC3	-2,88	4,61	6,07E-03	ENSG00000172296
LRTM2	6,14	3,49	6,07E-03	ENSG00000166159
FCGR3A	2,72	5,25	6,08E-03	ENSG00000203747
SOBP	-2,56	5,58	6,10E-03	ENSG00000112320
FZD10-DT	-3,34	5,07	6,11E-03	ENSG00000250208
SLCO5A1	-4,35	4,59	6,12E-03	ENSG00000137571
ATF3	2,56	6,88	6,13E-03	ENSG00000162772
GREM1	-4,82	4,59	6,13E-03	ENSG00000166923
CELSR1	-2,84	5,69	6,13E-03	ENSG00000075275
PRRT1	-3,64	6,26	6,13E-03	ENSG00000204314
LINC00689	4,03	4,74	6,16E-03	ENSG00000231419
SFRP4	2,81	7,91	6,17E-03	ENSG00000106483
SLCO1B3	-6,83	0,38	6,18E-03	ENSG00000111700
GPC1	2,82	7,71	6,20E-03	ENSG00000063660
WDR97	-3,26	4,01	6,20E-03	ENSG00000179698
LINC02593	-4,07	5,63	6,20E-03	ENSG00000223764
ELFN1-AS1	-5,68	1,89	6,21E-03	ENSG00000236081
MYL9	2,52	9,00	6,23E-03	ENSG00000101335
MXRA7	2,62	7,18	6,24E-03	ENSG00000182534
TP53I3	2,54	7,30	6,24E-03	ENSG00000115129
DDX11	-2,53	5,38	6,26E-03	ENSG00000013573
AC133065.1	5,30	1,79	6,29E-03	ENSG00000262151
MLIP	7,13	0,56	6,31E-03	ENSG00000146147
MYOC	-6,24	6,80	6,32E-03	ENSG00000034971
IL33	3,45	6,19	6,34E-03	ENSG00000137033
PKP3	5,18	2,21	6,35E-03	ENSG00000184363
DRP2	3,75	2,72	6,41E-03	ENSG00000102385
GREB1L	-7,05	3,51	6,46E-03	ENSG00000141449
KLHL26	2,88	5,39	6,46E-03	ENSG00000167487
RNF128	4,97	1,43	6,46E-03	ENSG00000133135
C4orf3	2,44	7,37	6,49E-03	ENSG00000164096
ETV4	-2,48	5,45	6,49E-03	ENSG00000175832
FAM107A	3,72	6,21	6,50E-03	ENSG00000168309
RDH8	5,13	1,70	6,52E-03	ENSG00000080511
LINC02253	-5,34	1,65	6,54E-03	ENSG00000259485
RF01888	-7,23	0,70	6,60E-03	ENSG00000277124
AC002398.2	2,80	5,15	6,61E-03	ENSG00000267328
SV2C	-5,41	3,44	6,62E-03	ENSG00000122012
PXDN	-2,58	8,33	6,62E-03	ENSG00000130508
REEP2	3,05	5,75	6,62E-03	ENSG00000132563
AP002800.1	-5,91	2,83	6,63E-03	ENSG00000280032
NPHP4	-3,19	6,85	6,63E-03	ENSG00000131697
SERPINA1	3,08	3,72	6,65E-03	ENSG00000197249
TNFSF10	2,71	4,87	6,66E-03	ENSG00000121858
HTN3	6,71	5,83	6,69E-03	ENSG00000205649
AC244021.1	3,95	2,62	6,69E-03	ENSG00000227082
OBSCN	-2,79	7,63	6,71E-03	ENSG00000154358
CD48	3,39	3,00	6,72E-03	ENSG00000117091
AC106872.5	7,10	0,54	6,73E-03	ENSG00000248632
RHOXF1-AS1	-3,85	1,58	6,73E-03	ENSG00000258545
CEP126	-2,73	5,44	6,77E-03	ENSG00000110318
AL139351.1	-7,56	0,96	6,79E-03	ENSG00000276923
APOBEC3B	4,46	1,76	6,83E-03	ENSG00000179750
JPH2	3,11	3,50	6,84E-03	ENSG00000149596

FAXC	4,15	2,97	6,84E-03	ENSG00000146267
MAST1	4,37	1,34	6,85E-03	ENSG00000105613
AC063960.2	-5,04	3,50	6,85E-03	ENSG00000285973
PTPRN	4,64	2,87	6,86E-03	ENSG00000054356
RGS11	-2,59	7,31	6,90E-03	ENSG00000076344
HES6	3,36	2,98	6,91E-03	ENSG00000144485
DNER	-5,14	2,92	6,95E-03	ENSG00000187957
SELE	5,57	2,52	6,96E-03	ENSG00000007908
MTTP	-4,16	2,77	6,98E-03	ENSG00000138823
CLDN5	2,71	5,42	6,98E-03	ENSG00000184113
TCEAL7	3,79	4,01	7,00E-03	ENSG00000182916
PYGB	2,72	8,25	7,02E-03	ENSG00000100994
SPINK5	-4,27	1,20	7,04E-03	ENSG00000133710
LINC01605	4,26	1,93	7,05E-03	ENSG00000253161
FBN2	-3,89	5,66	7,05E-03	ENSG00000138829
SLFNL1-AS1	-2,74	4,55	7,06E-03	ENSG00000281207
ICAM5	3,76	2,54	7,06E-03	ENSG00000105376
POLE	-2,80	6,97	7,07E-03	ENSG00000177084
FLJ16779	5,37	1,03	7,08E-03	ENSG00000275620
FGL1	-3,80	1,85	7,08E-03	ENSG00000104760
GPIHBP1	3,36	3,28	7,12E-03	ENSG00000277494
PRAME	-4,94	7,18	7,15E-03	ENSG00000185686
AGPAT4	-2,65	6,62	7,16E-03	ENSG00000026652
CD74	2,62	9,99	7,16E-03	ENSG00000019582
TAGAP	3,31	3,44	7,19E-03	ENSG00000164691
MAEL	4,89	1,47	7,22E-03	ENSG00000143194
DNAH8	-5,08	3,11	7,26E-03	ENSG00000124721
AF001548.1	2,93	3,85	7,39E-03	ENSG00000263065
PPL	3,21	4,22	7,40E-03	ENSG00000118898
WDR86-AS1	5,36	1,68	7,46E-03	ENSG00000243836
SFRP1	2,58	6,21	7,47E-03	ENSG00000104332
SEMA5A	-2,92	5,96	7,48E-03	ENSG00000112902
UBXN10-AS1	4,73	1,26	7,48E-03	ENSG00000225986
AC036108.2	2,90	4,12	7,50E-03	ENSG00000261054
NKX2-5	-6,01	1,56	7,57E-03	ENSG00000183072
RORC	5,04	0,71	7,58E-03	ENSG00000143365
L1CAM	3,70	3,21	7,58E-03	ENSG00000198910
ACP7	4,17	1,29	7,60E-03	ENSG00000183760
JAKMIP3	-3,10	3,51	7,64E-03	ENSG00000188385
P3H3	-2,47	6,71	7,64E-03	ENSG00000110811
STOX1	-5,76	3,68	7,69E-03	ENSG00000165730
TMEM52B	6,97	0,43	7,71E-03	ENSG00000165685
PIK3C2G	-5,65	5,24	7,72E-03	ENSG00000139144
HTR2A	-4,55	1,71	7,74E-03	ENSG00000102468
GPR65	3,63	2,61	7,75E-03	ENSG00000140030
MUC4	-5,17	5,15	7,78E-03	ENSG00000145113
TEPP	4,07	1,18	7,81E-03	ENSG00000159648
KCNT2	-3,59	5,48	7,81E-03	ENSG00000162687
PKN2-AS1	-4,45	0,93	7,83E-03	ENSG00000237505
AC233266.2	-4,21	1,68	7,84E-03	ENSG00000261600
PTPN14	-2,58	7,11	7,85E-03	ENSG00000152104
AL391845.2	4,58	2,50	7,85E-03	ENSG00000233542
ITIH5	-4,49	8,34	7,89E-03	ENSG00000123243
HMGN2P15	-4,60	3,86	7,89E-03	ENSG00000214578
LINC02587	-5,25	2,81	7,90E-03	ENSG00000229108
ROBO1	-2,51	6,29	7,91E-03	ENSG00000169855
MSX2	-5,19	4,19	7,91E-03	ENSG00000120149
ABCC1	-2,61	7,05	7,91E-03	ENSG00000103222
AC024267.6	-2,90	3,97	7,93E-03	ENSG00000266642
COL11A1	4,21	8,42	7,96E-03	ENSG00000060718
PTK2B	2,63	5,89	8,01E-03	ENSG00000120899
INHBB	2,61	4,75	8,02E-03	ENSG00000163083
FTH1P10	4,76	1,24	8,05E-03	ENSG00000223361
IRX4	6,30	5,09	8,05E-03	ENSG00000113430

ARHGEF26	-3,10	4,39	8,05E-03	ENSG00000114790
HSPB6	2,61	5,40	8,06E-03	ENSG00000004776
SVIP	3,15	5,72	8,06E-03	ENSG00000198168
AL450405.1	2,97	3,71	8,11E-03	ENSG00000230202
TCF7	2,81	5,33	8,12E-03	ENSG00000081059
MAMDC2	-2,65	4,73	8,18E-03	ENSG00000165072
TMEM163	-3,87	4,24	8,22E-03	ENSG00000152128
AC112255.1	5,28	0,95	8,24E-03	ENSG00000279024
FMOD	-3,14	8,45	8,27E-03	ENSG00000122176
TGM2	-4,08	9,72	8,28E-03	ENSG00000198959
CTXN1	3,42	4,13	8,37E-03	ENSG00000178531
AL590617.2	-3,41	5,88	8,37E-03	ENSG00000225177
CAVIN1	2,40	9,19	8,38E-03	ENSG00000177469
EGFR	-2,31	5,84	8,39E-03	ENSG00000146648
GLCE	2,68	5,73	8,46E-03	ENSG00000138604
SRGAP2C	2,65	5,10	8,47E-03	ENSG00000171943
LIF	2,77	4,63	8,48E-03	ENSG00000128342
MADCAM1	-3,74	2,59	8,50E-03	ENSG00000099866
HOXC13	-5,19	2,25	8,51E-03	ENSG00000123364
PHACTR1	-2,42	5,34	8,54E-03	ENSG00000112137
HOXD10	-4,03	2,71	8,55E-03	ENSG00000128710
RGS18	3,57	1,74	8,58E-03	ENSG00000150681
COL4A5	-2,92	5,79	8,75E-03	ENSG00000188153
INAFM2	2,61	4,82	8,75E-03	ENSG00000259330
AC110048.2	-7,34	0,77	8,76E-03	ENSG00000277152
ASIC3	-2,96	3,99	8,78E-03	ENSG00000213199
HES2	5,23	4,31	8,79E-03	ENSG00000069812
DNAJC6	3,84	4,30	8,80E-03	ENSG00000116675
TDRD6	-4,50	3,42	8,80E-03	ENSG00000180113
SNTB2	2,49	6,76	8,80E-03	ENSG00000168807
TENM1	-3,35	4,65	8,82E-03	ENSG00000009694
AL121929.2	-4,73	2,71	8,82E-03	ENSG00000273108
KCNH8	4,46	0,96	8,83E-03	ENSG00000183960
FTH1	2,42	10,68	8,84E-03	ENSG00000167996
SMC1B	-5,68	1,27	8,86E-03	ENSG00000077935
SMR3B	6,66	6,27	8,90E-03	ENSG00000171201
HLA-DOA	2,99	4,37	8,91E-03	ENSG00000204252
CHST3	2,75	7,41	8,93E-03	ENSG00000122863
AC245060.5	-3,32	3,05	8,94E-03	ENSG00000274422
SPRY2	2,43	5,40	8,98E-03	ENSG00000136158
LINC00623	3,54	2,53	9,03E-03	ENSG00000226067
ZNF219	2,53	6,21	9,03E-03	ENSG00000165804
KANK1	-2,63	5,93	9,04E-03	ENSG00000107104
AL355974.3	6,83	0,31	9,05E-03	ENSG00000276269
TTN-AS1	-2,53	6,19	9,05E-03	ENSG00000237298
GAS2L2	-5,21	4,26	9,09E-03	ENSG00000270765
CARMN	2,81	5,21	9,12E-03	ENSG00000249669
UBE2U	-6,74	3,21	9,14E-03	ENSG00000177414
ARSE	4,20	2,00	9,21E-03	ENSG00000157399
COL8A2	-2,43	5,93	9,21E-03	ENSG00000171812
MS4A2	-6,42	0,06	9,24E-03	ENSG00000149534
NPY1R	-4,53	6,38	9,26E-03	ENSG00000164128
CCL3	3,03	4,66	9,29E-03	ENSG00000277632
PLEKHH1	2,54	5,03	9,33E-03	ENSG00000054690
MTND4P12	4,34	3,59	9,36E-03	ENSG00000247627
TAS2R64P	-6,79	0,35	9,37E-03	ENSG00000256274
DIO3	2,95	3,68	9,38E-03	ENSG00000197406
AC006504.1	-3,78	2,00	9,43E-03	ENSG00000261770
AC008738.5	4,83	0,60	9,44E-03	ENSG00000267727
PIFO	3,40	2,72	9,45E-03	ENSG00000173947
RTP4	3,36	3,01	9,47E-03	ENSG00000136514
TUBB2A	2,52	6,32	9,50E-03	ENSG00000137267
BAGE2	-6,84	0,39	9,51E-03	ENSG00000187172
MED28P7	-6,84	0,39	9,52E-03	ENSG00000236542

CLMAT3	2,54	6,38	9,53E-03	ENSG00000249035
SCN3A	-5,32	5,28	9,54E-03	ENSG00000153253
LINC02268	-6,45	0,08	9,57E-03	ENSG00000248174
NR4A3	3,42	4,20	9,62E-03	ENSG00000119508
ANKEF1	-4,32	2,34	9,64E-03	ENSG00000132623
CERCAM	-2,48	8,37	9,68E-03	ENSG00000167123
LINC00869	3,64	2,81	9,69E-03	ENSG00000277147
RARRES1	3,18	4,14	9,76E-03	ENSG00000118849
AATK	2,73	5,23	9,77E-03	ENSG00000181409
CHL1-AS2	6,80	0,29	9,80E-03	ENSG00000224318
AC004080.2	-5,01	1,37	9,87E-03	ENSG00000253508
AP003064.1	3,44	1,80	9,87E-03	ENSG00000255126
UBE2Q2P2	-3,82	1,36	9,88E-03	ENSG00000259429
FAM218A	3,98	1,12	9,89E-03	ENSG00000250486
AC239868.1	-2,88	4,09	9,91E-03	ENSG00000261716
PNMA2	3,27	2,32	9,91E-03	ENSG00000240694
DBNDD1	3,26	2,73	9,94E-03	ENSG00000003249
DEPP1	2,45	7,49	1,01E-02	ENSG00000165507
SFXN4	-2,38	5,22	1,01E-02	ENSG00000183605
GBP1P1	3,43	1,80	1,01E-02	ENSG00000225492
OR2L13	-4,97	1,33	1,02E-02	ENSG00000196071
AL589935.1	3,26	2,71	1,02E-02	ENSG00000232295
HERC2P9	-2,39	6,99	1,02E-02	ENSG00000206149
AL627309.6	-4,04	4,49	1,02E-02	ENSG00000268903
AC022509.1	3,54	2,03	1,02E-02	ENSG00000255750
CAMK2A	3,92	3,05	1,03E-02	ENSG00000070808
DPP10	-5,35	4,63	1,03E-02	ENSG00000175497
COL6A6	-4,93	3,15	1,03E-02	ENSG00000206384
GABRA3	-5,56	1,17	1,03E-02	ENSG00000011677
EYA1	-5,59	2,55	1,03E-02	ENSG00000104313
SLK	-2,84	8,06	1,03E-02	ENSG00000065613
BEST1	2,35	10,47	1,03E-02	ENSG00000167995
AC098613.1	4,81	0,58	1,03E-02	ENSG00000223552
AP003774.1	4,81	0,58	1,03E-02	ENSG00000181908
DNM3OS	-2,48	5,67	1,04E-02	ENSG00000230630
AQP5	4,22	1,89	1,04E-02	ENSG00000161798
AC004492.1	-5,07	0,77	1,04E-02	ENSG00000272072
ZDHHC11	-3,19	3,28	1,04E-02	ENSG00000188818
C1QTNF2	-3,88	5,06	1,05E-02	ENSG00000145861
LINC01833	-6,60	3,09	1,05E-02	ENSG00000259439
TTYH1	4,05	4,01	1,05E-02	ENSG00000167614
LINC01268	4,01	2,60	1,05E-02	ENSG00000227502
GIMAP7	2,83	4,26	1,05E-02	ENSG00000179144
NEXMIF	-5,00	1,95	1,05E-02	ENSG00000050030
SLC5A9	3,49	4,90	1,05E-02	ENSG00000117834
AL157904.1	-5,43	2,14	1,05E-02	ENSG00000272715
AC009041.1	4,85	0,63	1,05E-02	ENSG00000260496
SLC6A3	4,89	2,79	1,05E-02	ENSG00000142319
SOX11	-3,67	5,22	1,05E-02	ENSG00000176887
FSIP1	2,68	4,54	1,05E-02	ENSG00000150667
SVOP	6,74	0,23	1,06E-02	ENSG00000166111
CARNS1	2,77	4,62	1,06E-02	ENSG00000172508
AC055876.2	-5,19	0,87	1,07E-02	ENSG00000254398
TP63	4,05	1,86	1,07E-02	ENSG00000073282
ALOXE3	-5,69	3,58	1,08E-02	ENSG00000179148
HTRA3	2,59	6,38	1,08E-02	ENSG00000170801
EPHA8	-4,85	0,60	1,08E-02	ENSG00000070886
CEBPA	3,29	4,11	1,09E-02	ENSG00000245848
DOK5	3,23	3,01	1,09E-02	ENSG00000101134
IGHG4	-5,36	8,04	1,10E-02	ENSG00000211892
CRTAM	3,75	1,44	1,10E-02	ENSG00000109943
ALCAM	-2,53	6,98	1,11E-02	ENSG00000170017
GBX1	-6,09	2,53	1,11E-02	ENSG00000164900
STK10	2,50	6,77	1,11E-02	ENSG00000072786

GOLGA6L10	-3,31	2,64	1,11E-02	ENSG00000278662
CCL4	3,19	3,65	1,11E-02	ENSG00000275302
USP32P3	-3,17	4,47	1,11E-02	ENSG00000189423
AC000032.1	3,88	2,51	1,11E-02	ENSG00000260246
AC005481.1	-6,99	0,51	1,11E-02	ENSG00000222012
SLC25A24	2,74	4,05	1,11E-02	ENSG00000085491
RASL12	2,47	4,83	1,11E-02	ENSG00000103710
AL355922.2	6,64	0,15	1,12E-02	ENSG00000258599
C10orf67	4,95	0,74	1,12E-02	ENSG00000179133
RINT1	-2,35	6,47	1,12E-02	ENSG00000135249
HLA-DRB1	2,40	7,83	1,12E-02	ENSG00000196126
MILR1	3,18	2,45	1,12E-02	ENSG00000271605
SYT14	4,72	1,33	1,12E-02	ENSG00000143469
LILRA2	4,03	2,32	1,12E-02	ENSG00000239998
AC003973.3	-3,55	4,01	1,12E-02	ENSG00000279377
RGS7	4,88	0,58	1,13E-02	ENSG00000182901
C6orf118	-5,73	2,70	1,13E-02	ENSG00000112539
HMGA1	2,54	5,52	1,13E-02	ENSG00000137309
CD163L1	-3,42	4,56	1,13E-02	ENSG00000177675
PPP1R14A	2,41	4,89	1,14E-02	ENSG00000167641
ZNF692	-2,35	7,80	1,15E-02	ENSG00000171163
SLC38A1	-2,40	5,69	1,15E-02	ENSG00000111371
PLA2R1	-2,26	5,63	1,15E-02	ENSG00000153246
FRK	-3,40	4,92	1,15E-02	ENSG00000111816
RHEX	-6,49	0,11	1,15E-02	ENSG00000263961
CDKN2B	3,10	3,75	1,15E-02	ENSG00000147883
EFCAB10	-2,25	5,70	1,15E-02	ENSG00000185055
ACACA	-2,55	6,63	1,15E-02	ENSG00000278540
FAM110C	-3,55	1,94	1,15E-02	ENSG00000184731
LAYN	2,44	5,13	1,15E-02	ENSG00000204381
AC016825.1	3,28	4,23	1,16E-02	ENSG00000232767
TFPI2	4,19	3,06	1,16E-02	ENSG00000105825
SCARF2	-2,45	6,52	1,16E-02	ENSG00000244486
SIGLEC10	3,28	3,74	1,17E-02	ENSG00000142512
INHBA-AS1	-5,01	0,71	1,18E-02	ENSG00000224116
APLNR	2,98	5,07	1,18E-02	ENSG00000134817
AP005131.2	-6,58	0,17	1,18E-02	ENSG00000267366
NID1	2,77	7,83	1,18E-02	ENSG00000116962
POTEKP	-6,91	0,44	1,19E-02	ENSG00000204434
HSPA12A	2,56	6,82	1,19E-02	ENSG00000165868
INPP5E	-2,25	6,90	1,19E-02	ENSG00000148384
SIGLEC8	3,79	2,55	1,19E-02	ENSG00000105366
SCX	-2,60	4,67	1,19E-02	ENSG00000260428
SNORC	4,00	4,58	1,19E-02	ENSG00000182600
SLPI	-6,30	8,30	1,19E-02	ENSG00000124107
NMB	2,85	4,27	1,20E-02	ENSG00000197696
AC006305.1	-3,91	2,65	1,20E-02	ENSG00000206129
C7orf57	4,54	1,11	1,21E-02	ENSG00000164746
SPAG4	-3,06	3,02	1,21E-02	ENSG00000061656
AL513217.1	2,80	4,35	1,21E-02	ENSG00000223774
SPAG6	-3,72	2,26	1,21E-02	ENSG00000077327
DDAH1	2,41	4,90	1,22E-02	ENSG00000153904
AC100793.4	-4,82	2,79	1,22E-02	ENSG00000273650
GPR37	-4,27	5,77	1,22E-02	ENSG00000170775
AL807752.3	4,06	1,08	1,22E-02	ENSG00000231864
SGPP2	-4,23	2,07	1,22E-02	ENSG00000163082
AL049552.1	-3,87	1,40	1,22E-02	ENSG00000234084
AL031282.1	-2,99	3,44	1,22E-02	ENSG00000227775
LYPD6	-3,73	3,87	1,22E-02	ENSG00000187123
TCAF1	-3,10	6,66	1,22E-02	ENSG00000198420
EBLN1	-6,42	0,06	1,23E-02	ENSG00000223601
GPRIN2	-6,42	0,06	1,23E-02	ENSG00000204175
CNTN3	-3,82	2,75	1,23E-02	ENSG00000113805
ACY3	5,16	0,95	1,23E-02	ENSG00000132744

AC003991.1	3,25	3,37	1,23E-02	ENSG00000228113
PEG10	3,45	5,37	1,23E-02	ENSG00000242265
GCNT4	-3,78	4,35	1,24E-02	ENSG00000176928
ATL1	2,63	4,84	1,25E-02	ENSG00000198513
FGFBP3	3,27	2,94	1,26E-02	ENSG00000174721
RPL10P9	3,41	1,75	1,26E-02	ENSG00000233913
PTGS2	3,09	2,89	1,26E-02	ENSG00000073756
C2CD4B	4,57	1,06	1,27E-02	ENSG00000205502
EFNB1	2,40	5,05	1,27E-02	ENSG00000090776
MYOZ1	3,97	1,67	1,27E-02	ENSG00000177791
LRRC10B	3,64	1,15	1,27E-02	ENSG00000204950
RNF165	4,08	2,86	1,27E-02	ENSG00000141622
AP000697.1	-6,30	-0,02	1,28E-02	ENSG00000224269
PPM1H	-3,20	4,90	1,28E-02	ENSG00000111110
ANKRD20A17P	-4,76	1,16	1,29E-02	ENSG00000251056
LINC02323	4,77	0,50	1,29E-02	ENSG00000259230
KMO	3,15	4,07	1,30E-02	ENSG00000117009
CDSN	-4,15	1,79	1,30E-02	ENSG00000204539
SRP68P3	-3,78	1,82	1,30E-02	ENSG00000237911
SLC6A6	2,57	6,26	1,30E-02	ENSG00000131389
BST2	2,31	5,33	1,30E-02	ENSG00000130303
ZEB2	2,29	7,01	1,30E-02	ENSG00000169554
SEPT5	-3,75	1,30	1,30E-02	ENSG00000184702
DCN	2,26	8,78	1,30E-02	ENSG00000011465
MAPT	-3,34	5,27	1,31E-02	ENSG00000186868
VASH1	2,38	6,22	1,31E-02	ENSG00000071246
IGFL4	3,47	1,71	1,32E-02	ENSG00000204869
GIMAP8	2,59	4,67	1,32E-02	ENSG00000171115
MKRN3	-5,57	2,90	1,32E-02	ENSG00000179455
MROH7	-3,88	2,40	1,32E-02	ENSG00000184313
AC015909.3	7,05	0,23	1,33E-02	ENSG00000261959
WT1	7,05	0,23	1,33E-02	ENSG00000184937
FTH1P16	4,72	0,41	1,33E-02	ENSG00000227376
IGFBP5	2,37	8,55	1,33E-02	ENSG00000115461
AL691432.1	-4,09	1,04	1,33E-02	ENSG00000269737
AL138781.1	4,14	2,94	1,33E-02	ENSG00000260193
SLC35F2	-3,03	5,12	1,34E-02	ENSG00000110660
NEK6	2,40	6,43	1,34E-02	ENSG00000119408
MSC	2,92	3,19	1,34E-02	ENSG00000178860
AC148477.1	6,53	0,07	1,34E-02	ENSG00000255916
BPIFA2	3,55	3,42	1,34E-02	ENSG00000131050
RUSC1	-2,18	7,25	1,34E-02	ENSG00000160753
LZTS1	2,47	5,55	1,34E-02	ENSG00000061337
BVES-AS1	3,95	1,70	1,35E-02	ENSG00000203808
DNAH5	-4,36	4,02	1,35E-02	ENSG00000039139
IRX1	5,42	0,95	1,35E-02	ENSG00000170549
ITGB2	2,31	5,60	1,35E-02	ENSG00000160255
SYT16	-6,04	2,44	1,36E-02	ENSG00000139973
AP001099.1	-4,14	1,08	1,36E-02	ENSG00000265728
MPIG6B	-4,75	3,24	1,36E-02	ENSG00000204420
IGF2BP2	-2,96	4,68	1,36E-02	ENSG00000073792
AC079298.1	-5,06	0,76	1,36E-02	ENSG00000278981
FUT1	3,35	3,86	1,37E-02	ENSG00000174951
MEIS3	2,64	5,11	1,37E-02	ENSG00000105419
ZNF273	-2,35	5,17	1,37E-02	ENSG00000198039
EML6	-3,46	4,98	1,37E-02	ENSG00000214595
PMP22	2,42	8,24	1,37E-02	ENSG00000109099
AC007637.1	-6,35	0,00	1,38E-02	ENSG00000256139
SDS	3,60	1,14	1,38E-02	ENSG00000135094
IGLV2-14	4,33	1,54	1,38E-02	ENSG00000211666
AL031733.2	3,69	0,81	1,38E-02	ENSG00000241666
AC026785.2	4,49	0,92	1,38E-02	ENSG00000248223
POF1B	-4,53	2,38	1,38E-02	ENSG00000124429
LINC00561	-6,82	0,37	1,38E-02	ENSG00000261206

KLK2	-6,31	-0,01	1,39E-02	ENSG00000167751
GIMAP6	2,59	4,46	1,39E-02	ENSG00000133561
HAS2	2,83	3,42	1,39E-02	ENSG00000170961
LINGO1	2,68	4,43	1,39E-02	ENSG00000169783
MRC2	-2,46	7,77	1,39E-02	ENSG00000011028
AL365273.1	-2,83	3,77	1,39E-02	ENSG00000240527
AC011239.2	-4,44	3,68	1,39E-02	ENSG00000279526
FGF7	2,98	4,64	1,40E-02	ENSG00000140285
NPM2	3,71	1,69	1,40E-02	ENSG00000158806
AC018467.1	6,21	-0,22	1,40E-02	ENSG00000232451
MOG	4,17	0,73	1,40E-02	ENSG00000204655
LILRA1	3,67	1,96	1,41E-02	ENSG00000104974
GFPT2	2,45	6,07	1,41E-02	ENSG00000131459
FOLR1	6,28	-0,19	1,41E-02	ENSG00000110195
ALDH3B1	2,60	4,97	1,41E-02	ENSG00000006534
SLC30A3	3,89	2,36	1,42E-02	ENSG00000115194
BRDT	-4,41	3,14	1,42E-02	ENSG00000137948
CLEC7A	3,30	4,65	1,42E-02	ENSG00000172243
EPHB6	-3,29	6,30	1,42E-02	ENSG00000106123
RAP1A	2,17	6,60	1,42E-02	ENSG00000116473
AC026316.4	6,26	-0,18	1,42E-02	ENSG00000285051
FTH1P2	6,26	-0,18	1,43E-02	ENSG00000234975
PLCH1	-4,47	4,58	1,43E-02	ENSG00000114805
GPR88	-5,47	3,56	1,43E-02	ENSG00000181656
AFDN-DT	-3,55	1,63	1,43E-02	ENSG00000198221
GATA3-AS1	-6,19	-0,10	1,43E-02	ENSG00000197308
CRABP2	-2,90	8,33	1,43E-02	ENSG00000143320
EPHX4	3,23	4,15	1,43E-02	ENSG00000172031
BCL2	2,72	3,94	1,44E-02	ENSG00000171791
VIM-AS1	2,48	8,44	1,44E-02	ENSG00000229124
FSTL3	2,51	7,63	1,44E-02	ENSG00000070404
SOX18	2,77	5,07	1,44E-02	ENSG00000203883
RAVER2	-2,74	5,40	1,45E-02	ENSG00000162437
FOXF2	-3,43	4,95	1,45E-02	ENSG00000137273
CFAP100	-3,93	0,51	1,45E-02	ENSG00000163885
SF3B4	-2,35	7,27	1,45E-02	ENSG00000143368
CYSTM1	2,17	5,40	1,45E-02	ENSG00000120306
BNC1	-4,16	4,76	1,46E-02	ENSG00000169594
FGF18	-4,33	6,53	1,46E-02	ENSG00000156427
ADGRV1	-3,81	2,17	1,46E-02	ENSG00000164199
LINC02468	-6,72	0,30	1,47E-02	ENSG00000256499
LINC00517	6,59	-0,08	1,48E-02	ENSG00000259091
AP3B2	4,31	1,84	1,48E-02	ENSG00000103723
PLS3-AS1	-3,76	1,66	1,48E-02	ENSG00000271826
BARX1-DT	-6,52	0,14	1,48E-02	ENSG00000235601
SLC25A4	2,45	6,17	1,49E-02	ENSG00000151729
AC108474.1	-3,68	3,44	1,49E-02	ENSG00000250971
AL034397.3	3,48	2,43	1,49E-02	ENSG00000274536
AC025271.4	-6,26	-0,06	1,50E-02	ENSG00000276772
CDAN1	-2,51	6,18	1,50E-02	ENSG00000140326
SBSPON	2,44	5,37	1,51E-02	ENSG00000164764
ATP10A	-3,27	6,78	1,51E-02	ENSG00000206190
PDLIM3	2,17	6,49	1,51E-02	ENSG00000154553
AC103564.3	-5,51	2,20	1,52E-02	ENSG00000274353
TBX18	-2,76	6,42	1,52E-02	ENSG00000112837
SFTPD-AS1	-4,03	1,69	1,52E-02	ENSG00000273372
MNDA	2,87	3,18	1,52E-02	ENSG00000163563
RASGRP2	2,41	4,66	1,52E-02	ENSG00000068831
AL049557.2	3,27	1,48	1,52E-02	ENSG00000284830
CSRP2	-3,08	6,59	1,53E-02	ENSG00000175183
IL13RA2	-4,45	4,19	1,53E-02	ENSG00000123496
NOMO1	-2,27	6,40	1,53E-02	ENSG00000103512
TMEM132E	3,78	2,74	1,53E-02	ENSG00000181291
FLVCR2	3,12	3,79	1,53E-02	ENSG00000119686

SAMD12	-4,02	5,26	1,53E-02	ENSG00000177570
SLC4A2	-2,16	7,72	1,54E-02	ENSG00000164889
SORBS1	2,67	7,82	1,54E-02	ENSG00000095637
LINC00923	-4,37	4,08	1,54E-02	ENSG00000251209
LIMA1	2,19	7,60	1,54E-02	ENSG00000050405
AL451064.2	-5,49	1,09	1,55E-02	ENSG00000279659
CD180	3,54	1,77	1,55E-02	ENSG00000134061
FSIP2	-3,40	3,58	1,56E-02	ENSG00000188738
VIM	2,21	10,84	1,56E-02	ENSG00000026025
TNXB	-3,40	7,70	1,57E-02	ENSG00000168477
MMP25-AS1	-2,47	4,89	1,57E-02	ENSG00000261971
FLNC	2,54	7,52	1,57E-02	ENSG00000128591
GBAP1	-2,61	4,05	1,57E-02	ENSG00000160766
PRCD	2,64	4,41	1,58E-02	ENSG00000214140
RF02179	-4,66	0,45	1,58E-02	ENSG00000275359
COL6A5	-6,45	0,08	1,59E-02	ENSG00000172752
RNF112	-3,52	3,80	1,59E-02	ENSG00000128482
SLC16A10	-3,91	0,50	1,59E-02	ENSG00000112394
AL596223.2	6,10	-0,30	1,59E-02	ENSG00000231748
PKD1P6	-2,82	5,10	1,60E-02	ENSG00000250251
ITGA10	-4,02	9,16	1,60E-02	ENSG00000143127
ZBTB20-AS1	-6,61	0,19	1,60E-02	ENSG00000241560
AFAP1L2	2,53	7,25	1,60E-02	ENSG00000169129
AC105046.1	3,63	1,18	1,61E-02	ENSG00000261026
AC116351.2	-6,46	0,10	1,61E-02	ENSG00000272347
AC148477.7	6,33	-0,09	1,61E-02	ENSG00000279113
APOBEC3B-AS1	3,82	0,87	1,61E-02	ENSG00000249310
PHYHIP	2,86	3,68	1,61E-02	ENSG00000168490
PTGES3L	4,32	0,96	1,62E-02	ENSG00000267060
SLC12A8	3,01	5,31	1,62E-02	ENSG00000221955
BMPR1B	-2,34	4,79	1,62E-02	ENSG00000138696
CEP83	-2,17	5,53	1,62E-02	ENSG00000173588
CLEC12A	3,50	2,29	1,63E-02	ENSG00000172322
MAMLD1	2,92	5,15	1,63E-02	ENSG00000013619
MAP7	-2,86	3,98	1,63E-02	ENSG00000135525
AC022784.1	-3,79	2,33	1,63E-02	ENSG00000248538
AC099489.1	-2,57	4,24	1,64E-02	ENSG00000188897
PTPRC	2,37	4,77	1,64E-02	ENSG00000081237
ADRB1	-4,09	2,28	1,64E-02	ENSG00000043591
AC004233.3	6,37	-0,06	1,64E-02	ENSG00000272079
LINC01705	6,37	-0,06	1,64E-02	ENSG00000232679
TMC7	2,77	3,37	1,65E-02	ENSG00000170537
PCP4	-4,67	2,56	1,65E-02	ENSG00000183036
TCEAL8	2,20	6,10	1,65E-02	ENSG00000180964
AC148477.2	-4,42	3,85	1,65E-02	ENSG00000256542
AL133415.1	2,45	8,78	1,65E-02	ENSG00000234961
PPP1R15A	2,21	6,83	1,66E-02	ENSG00000087074
TTYH3	-2,22	7,43	1,67E-02	ENSG00000136295
HTR7	4,74	0,49	1,67E-02	ENSG00000148680
IRS2	2,94	5,96	1,67E-02	ENSG00000185950
RBM38	2,50	5,14	1,67E-02	ENSG00000132819
BMS1P10	-4,46	3,20	1,67E-02	ENSG00000237238
FTH1P11	3,61	1,09	1,67E-02	ENSG00000237264
AC021088.1	-3,78	1,31	1,68E-02	ENSG00000250619
TNFAIP8L1	2,28	4,89	1,68E-02	ENSG00000185361
RABGAP1L	-2,48	7,19	1,68E-02	ENSG00000152061
DSP	-4,05	5,11	1,68E-02	ENSG00000096696
CADPS2	-2,37	6,09	1,68E-02	ENSG00000081803
WDPCP	-2,61	4,47	1,71E-02	ENSG00000143951
TNFSF14	3,49	1,34	1,71E-02	ENSG00000125735
PRDM16	-3,06	5,42	1,71E-02	ENSG00000142611
CHST11	2,39	5,52	1,71E-02	ENSG00000171310
BCL11A	3,64	1,40	1,71E-02	ENSG00000119866
SELPLG	2,54	4,64	1,72E-02	ENSG00000110876

ATF5	2,31	4,88	1,72E-02	ENSG00000169136
PTAFR	2,91	3,30	1,73E-02	ENSG00000169403
CAV1	2,23	8,19	1,73E-02	ENSG00000105974
AOC3	2,15	5,71	1,73E-02	ENSG00000131471
AC009779.3	3,56	2,00	1,73E-02	ENSG00000258311
FENDRR	-4,03	3,80	1,74E-02	ENSG00000268388
TMEM26	-3,41	3,77	1,75E-02	ENSG00000196932
SMC2-AS1	-4,41	2,46	1,75E-02	ENSG00000270332
GRIA3	-3,24	3,17	1,76E-02	ENSG00000125675
VWA7	-3,82	1,72	1,76E-02	ENSG00000204396
CNKSR2	-3,53	5,91	1,76E-02	ENSG00000149970
ERG	-2,58	7,08	1,76E-02	ENSG00000157554
HAS2-AS1	3,82	1,18	1,76E-02	ENSG00000248690
MINPP1	-3,09	6,85	1,76E-02	ENSG00000107789
FUT8-AS1	3,52	1,31	1,76E-02	ENSG00000276116
ADCY9	-2,68	6,51	1,76E-02	ENSG00000162104
PPFIA4	-3,15	5,85	1,77E-02	ENSG00000143847
AC148477.5	-4,51	3,58	1,78E-02	ENSG00000278872
MFSD2A	3,22	2,95	1,78E-02	ENSG00000168389
AC027288.3	4,05	0,64	1,79E-02	ENSG00000257894
EVI2B	2,80	3,88	1,80E-02	ENSG00000185862
KRT18	-3,65	8,85	1,80E-02	ENSG00000111057
DKK3	-2,39	8,17	1,81E-02	ENSG00000050165
ASH2LP3	-6,51	0,13	1,81E-02	ENSG00000225724
ST8SIA2	-3,62	4,61	1,81E-02	ENSG00000140557
MAPK8IP3	-2,15	8,88	1,81E-02	ENSG00000138834
AF111169.3	2,76	3,83	1,81E-02	ENSG00000259081
LINC02594	5,68	2,06	1,82E-02	ENSG00000267440
AC129492.1	-4,70	1,50	1,82E-02	ENSG00000214999
GRIA2	6,86	0,06	1,82E-02	ENSG00000120251
CACNA1H	2,47	4,97	1,82E-02	ENSG00000196557
NR2F2	2,46	7,05	1,83E-02	ENSG00000185551
P2RY12	3,93	1,43	1,83E-02	ENSG00000169313
MGST1	-3,75	4,41	1,83E-02	ENSG00000008394
AC073410.1	-3,79	0,41	1,83E-02	ENSG00000236047
JPH1	3,63	3,17	1,83E-02	ENSG00000104369
AC145124.1	4,80	0,64	1,84E-02	ENSG00000255495
RN7SKP23	-2,82	3,05	1,84E-02	ENSG00000280039
IQCA1	-2,82	3,68	1,84E-02	ENSG00000132321
DCDC1	-5,28	2,62	1,84E-02	ENSG00000170959
FTL	2,25	10,22	1,84E-02	ENSG00000087086
CRHBP	-3,68	4,47	1,84E-02	ENSG00000145708
FBLN7	-2,58	3,74	1,85E-02	ENSG00000144152
SYNPO2	2,14	6,15	1,85E-02	ENSG00000172403
TRPC1	-2,07	5,62	1,86E-02	ENSG00000144935
LXN	2,39	4,83	1,86E-02	ENSG00000079257
SELL	2,79	2,88	1,86E-02	ENSG00000188404
TPM1-AS	2,76	4,23	1,86E-02	ENSG00000259498
PHF21B	3,79	2,29	1,86E-02	ENSG00000056487
BMS1P22	-6,56	0,18	1,86E-02	ENSG00000232775
ADORA2B	3,39	4,12	1,87E-02	ENSG00000170425
LGR6	3,54	5,02	1,87E-02	ENSG00000133067
FAM102B	2,42	4,70	1,87E-02	ENSG00000162636
AKAP9	-2,09	7,38	1,87E-02	ENSG00000127914
PAMR1	-3,81	7,47	1,87E-02	ENSG00000149090
LOH12CR2	3,25	1,68	1,87E-02	ENSG00000205791
SASH3	2,91	3,08	1,87E-02	ENSG00000122122
KLHL17	-2,05	5,72	1,88E-02	ENSG00000187961
MLXIPL	3,46	2,78	1,89E-02	ENSG00000009950
LINC01684	-3,89	3,16	1,89E-02	ENSG00000237484
C22orf31	-3,80	0,41	1,89E-02	ENSG00000100249
AC244517.11	-4,02	0,98	1,90E-02	ENSG00000280029
SHF	2,54	4,55	1,90E-02	ENSG00000138606
AC034238.1	3,03	2,20	1,90E-02	ENSG00000240535

CCDC25	2,29	6,70	1,90E-02	ENSG00000147419
MAGEA1	-6,01	2,53	1,91E-02	ENSG00000198681
ISL2	-5,21	0,87	1,91E-02	ENSG00000159556
AP005131.6	-3,83	0,83	1,92E-02	ENSG00000267702
DIO2	2,35	6,68	1,92E-02	ENSG00000211448
PLA2G2A	3,35	5,64	1,92E-02	ENSG00000188257
MFAP2	-3,16	7,04	1,92E-02	ENSG00000117122
LINC01001	-3,17	2,03	1,93E-02	ENSG00000230724
EIF3L	2,37	5,97	1,93E-02	ENSG00000100129
AL133325.3	-3,18	3,55	1,93E-02	ENSG00000278041
MPP6	-2,62	4,68	1,93E-02	ENSG00000105926
OCLM	-6,27	-0,04	1,94E-02	ENSG00000262180
PPFIA2	-2,65	4,72	1,94E-02	ENSG00000139220
NHSL1	-2,89	6,12	1,94E-02	ENSG00000135540
SH3KBP1	2,17	6,21	1,94E-02	ENSG00000147010
CLEC4GP1	-5,95	-0,27	1,94E-02	ENSG00000268297
PRAM1	3,28	3,04	1,95E-02	ENSG00000133246
AC018804.1	-5,95	-0,27	1,95E-02	ENSG00000227632
DPF3	3,30	2,44	1,95E-02	ENSG00000205683
CHRNA1	3,41	1,27	1,96E-02	ENSG00000138435
MAP4	2,18	8,38	1,97E-02	ENSG00000047849
ADM	-2,77	5,83	1,97E-02	ENSG00000148926
ADRA1B	-4,53	2,88	1,98E-02	ENSG00000170214
OR2M3	-6,45	0,09	1,98E-02	ENSG00000228198
AC084855.1	-3,10	2,27	1,98E-02	ENSG00000254744
NAV2	-2,36	6,09	1,99E-02	ENSG00000166833
BCAT1	2,45	3,99	1,99E-02	ENSG00000060982
SLC29A1	-3,01	8,19	1,99E-02	ENSG00000112759
AC145423.3	-3,72	0,35	1,99E-02	ENSG00000278112
AC092338.2	-3,72	0,35	1,99E-02	ENSG00000260790
CTHRC1	2,20	5,76	2,00E-02	ENSG00000164932
TREM1	6,14	-0,24	2,00E-02	ENSG00000124731
NUP62CL	-4,17	2,37	2,01E-02	ENSG00000198088
MAGI2	-2,75	4,88	2,01E-02	ENSG00000187391
MYO3A	4,36	1,68	2,01E-02	ENSG00000095777
AL512622.1	-3,53	1,79	2,01E-02	ENSG00000235010
AC025259.3	2,42	8,50	2,02E-02	ENSG00000259884
HILS1	4,04	1,13	2,02E-02	ENSG00000188662
TBX1	-3,09	5,18	2,02E-02	ENSG00000184058
CLDN23	-3,43	3,37	2,03E-02	ENSG00000253958
CCDC160	-4,72	1,53	2,03E-02	ENSG00000203952
SLC6A1	2,53	4,08	2,03E-02	ENSG00000157103
AL136164.4	3,23	2,01	2,03E-02	ENSG00000279312
IGBP1	2,06	5,56	2,03E-02	ENSG00000089289
RARRES2	2,25	5,07	2,03E-02	ENSG00000106538
CELF5	3,40	1,17	2,04E-02	ENSG00000161082
NEB	-2,50	4,04	2,04E-02	ENSG00000183091
AL023284.4	-3,70	0,74	2,04E-02	ENSG00000260418
AC116345.1	3,31	2,59	2,04E-02	ENSG00000249743
KIAA1841	-2,18	4,94	2,05E-02	ENSG00000162929
AL589880.1	6,05	-0,37	2,05E-02	ENSG00000229939
ALOX12B	-5,09	2,76	2,05E-02	ENSG00000179477
CCDC144A	-3,53	2,32	2,05E-02	ENSG00000170160
DPYSL2	2,24	7,72	2,05E-02	ENSG00000092964
LINC01597	-3,84	1,53	2,06E-02	ENSG00000205611
IQCA1L	-6,07	-0,19	2,06E-02	ENSG00000278685
APOL3	2,37	5,36	2,06E-02	ENSG00000128284
SCCPDH	2,44	6,42	2,06E-02	ENSG00000143653
AC010719.1	-3,21	1,36	2,07E-02	ENSG00000270933
LMX1A	-3,84	0,45	2,07E-02	ENSG00000162761
WISP1	-3,01	6,63	2,07E-02	ENSG00000104415
FAM3C	-2,04	6,92	2,07E-02	ENSG00000196937
ITGA3	2,53	7,10	2,08E-02	ENSG00000005884
GBP4	2,36	4,33	2,08E-02	ENSG00000162654

ANGPTL7	3,95	2,45	2,08E-02	ENSG00000171819
TFAP2A-AS2	3,51	2,16	2,08E-02	ENSG00000285278
ALDH1L2	2,49	4,53	2,08E-02	ENSG00000136010
AC098869.2	6,19	-0,20	2,08E-02	ENSG00000250893
MPPED2	-3,01	4,25	2,09E-02	ENSG00000066382
PRSS35	-3,35	3,56	2,09E-02	ENSG00000146250
AP005131.7	-3,80	0,42	2,10E-02	ENSG00000272746
ATP4B	-6,10	-0,17	2,10E-02	ENSG00000186009
CYP26B1	-2,43	4,36	2,10E-02	ENSG00000003137
MYCT1	2,77	4,42	2,10E-02	ENSG00000120279
SEC14L5	3,52	1,62	2,10E-02	ENSG00000103184
CD24	3,05	2,64	2,10E-02	ENSG00000272398
C14orf132	2,13	5,34	2,11E-02	ENSG00000227051
PI4KAP1	-2,13	5,51	2,11E-02	ENSG00000274602
AC025280.3	-3,72	1,95	2,11E-02	ENSG00000285848
AC093525.6	3,86	1,26	2,11E-02	ENSG00000261613
TMEM156	3,89	1,07	2,12E-02	ENSG00000121895
AC139256.1	-2,57	3,91	2,12E-02	ENSG00000260735
AC105052.2	-3,28	1,11	2,13E-02	ENSG00000213385
CCDC184	3,33	1,10	2,13E-02	ENSG00000177875
MSC-AS1	2,74	3,09	2,13E-02	ENSG00000235531
SLC9A5	2,55	5,17	2,14E-02	ENSG00000135740
ITGB5	-2,03	7,96	2,14E-02	ENSG00000082781
B3GALT5	4,10	2,62	2,15E-02	ENSG00000183778
FRMD5	2,43	5,08	2,16E-02	ENSG00000171877
MCHR1	4,47	0,33	2,16E-02	ENSG00000128285
CYBB	2,27	5,01	2,16E-02	ENSG00000165168
HIST1H2BH	-5,05	2,05	2,16E-02	ENSG00000275713
TTC39A	-3,22	1,67	2,16E-02	ENSG00000085831
HSPB7	2,77	5,33	2,16E-02	ENSG00000173641
ATP1B1	-2,08	6,31	2,18E-02	ENSG00000143153
SLC1A5	2,01	5,51	2,18E-02	ENSG00000105281
AL133346.1	2,86	3,32	2,18E-02	ENSG00000227220
LINC01088	2,97	2,72	2,19E-02	ENSG00000249307
COBLL1	-2,38	6,37	2,19E-02	ENSG00000082438
PABPC1P4	3,33	1,92	2,19E-02	ENSG00000255642
ETNK2	-2,67	5,77	2,19E-02	ENSG00000143845
ANXA1	2,05	8,06	2,20E-02	ENSG00000135046
PCOLCE	-2,54	8,90	2,20E-02	ENSG00000106333
PRKAB2	2,26	6,20	2,21E-02	ENSG00000131791
MEIOC	2,64	4,04	2,21E-02	ENSG00000180336
MICE	-4,42	0,27	2,21E-02	ENSG00000273340
YJEFN3	-2,45	4,53	2,22E-02	ENSG00000250067
PCSK5	-2,44	5,46	2,23E-02	ENSG00000099139
AP001189.1	2,87	2,36	2,23E-02	ENSG00000236304
NDST3	-3,96	2,40	2,23E-02	ENSG00000164100
CD207	3,36	0,58	2,24E-02	ENSG00000116031
PLPPR5	3,86	1,44	2,24E-02	ENSG00000117598
AC011558.1	2,13	5,04	2,24E-02	ENSG00000279753
TGFB2	-2,72	3,78	2,24E-02	ENSG00000092969
AL390729.1	4,67	0,54	2,24E-02	ENSG00000224167
TAF13	2,32	4,56	2,24E-02	ENSG00000197780
LINC01563	4,33	0,20	2,25E-02	ENSG00000236819
CDC20P1	-4,13	1,75	2,25E-02	ENSG00000231007
AL008729.2	-2,51	4,16	2,25E-02	ENSG00000272379
GDPD2	4,61	1,92	2,25E-02	ENSG00000130055
TMEM158	2,76	3,33	2,25E-02	ENSG00000249992
ZNF575	2,49	4,24	2,26E-02	ENSG00000176472
ITGAX	2,68	5,90	2,26E-02	ENSG00000140678
CBX6	2,05	6,53	2,26E-02	ENSG00000183741
RPLP0P2	2,97	4,03	2,26E-02	ENSG00000243742
LINC01310	3,13	1,53	2,27E-02	ENSG00000205632
LINC01748	4,66	1,76	2,27E-02	ENSG00000226476
INTS4P1	-2,80	3,36	2,27E-02	ENSG00000164669

SCUBE1	-3,25	3,26	2,28E-02	ENSG00000159307
AP001330.5	3,64	1,51	2,28E-02	ENSG00000271882
AC010973.2	-2,01	6,88	2,29E-02	ENSG00000244151
LINC02551	-4,01	1,22	2,29E-02	ENSG00000254842
AC112721.1	4,32	0,94	2,29E-02	ENSG00000222022
MRAS	2,18	7,16	2,30E-02	ENSG00000158186
LIPH	-4,77	2,58	2,30E-02	ENSG00000163898
GPRC5D	-3,67	0,32	2,30E-02	ENSG00000111291
KCNIP1	3,78	1,89	2,30E-02	ENSG00000182132
KCNQ2	4,22	2,26	2,31E-02	ENSG00000075043
RORA-AS1	-2,96	4,35	2,31E-02	ENSG00000245534
PKP1	-3,87	4,00	2,32E-02	ENSG00000081277
CFAP47	-4,50	3,75	2,32E-02	ENSG00000165164
AL121985.1	2,82	2,71	2,32E-02	ENSG00000228863
OR7E38P	-5,98	-0,24	2,32E-02	ENSG00000183444
GFRA1	3,43	8,89	2,32E-02	ENSG00000151892
KIAA0040	-2,15	6,20	2,33E-02	ENSG00000235750
ZNF300	-2,01	5,46	2,33E-02	ENSG00000145908
ERC2	-3,37	0,99	2,33E-02	ENSG00000187672
IL6	3,83	3,07	2,33E-02	ENSG00000136244
AL445437.1	-6,25	-0,05	2,34E-02	ENSG00000279778
AL627309.7	-5,07	1,80	2,34E-02	ENSG00000269981
CHEK2P2	-6,25	-0,05	2,34E-02	ENSG00000259156
AC019197.1	-4,20	2,56	2,34E-02	ENSG00000236283
DHCR7	-2,51	3,84	2,34E-02	ENSG00000172893
SLC2A10	-2,04	5,39	2,34E-02	ENSG00000197496
TFEC	2,87	3,21	2,35E-02	ENSG00000105967
LINP1	6,08	-0,28	2,35E-02	ENSG00000223784
UNC13A	3,09	2,38	2,35E-02	ENSG00000130477
HGF	2,91	3,31	2,35E-02	ENSG00000019991
KIT	-2,38	4,99	2,35E-02	ENSG00000157404
PRRG3	3,04	3,09	2,35E-02	ENSG00000130032
TM6SF2	-4,61	2,74	2,36E-02	ENSG00000213996
AC137056.1	3,36	2,39	2,36E-02	ENSG00000262995
AC141557.2	-6,05	-0,20	2,36E-02	ENSG00000278635
IGBP1-AS2	2,57	3,45	2,37E-02	ENSG00000220925
MPPED1	4,36	1,16	2,37E-02	ENSG00000186732
IGFBP7-AS1	2,14	6,47	2,37E-02	ENSG00000245067
LAPTM5	2,15	6,67	2,38E-02	ENSG00000162511
AC004918.3	-3,96	0,92	2,38E-02	ENSG00000270157
SMIM5	2,98	3,23	2,38E-02	ENSG00000204323
DNAH2	-3,44	3,19	2,38E-02	ENSG00000183914
MT1X	-2,59	3,78	2,38E-02	ENSG00000187193
EMB	-2,90	6,58	2,38E-02	ENSG00000170571
TMEM98	2,14	6,01	2,39E-02	ENSG00000006042
FHL2	2,45	6,05	2,40E-02	ENSG00000115641
DUSP6	2,25	7,18	2,41E-02	ENSG00000139318
B3GLCT	-2,02	5,44	2,41E-02	ENSG00000187676
RAET1L	-4,84	0,56	2,41E-02	ENSG00000155918
DAPP1	3,12	1,90	2,41E-02	ENSG00000070190
CSF2RB	2,78	3,31	2,41E-02	ENSG00000100368
TCN2	2,38	4,84	2,41E-02	ENSG00000185339
AC114296.1	4,58	2,74	2,41E-02	ENSG00000250378
MBOAT1	-2,15	4,87	2,42E-02	ENSG00000172197
PCDH7	3,08	4,43	2,43E-02	ENSG00000169851
CTSK	-2,36	8,17	2,43E-02	ENSG00000143387
RRN3P3	-2,38	4,56	2,43E-02	ENSG00000257122
ACSBG2	-3,55	2,05	2,43E-02	ENSG00000130377
RCSD1	2,30	4,25	2,44E-02	ENSG00000198771
LINC01115	-6,13	-0,14	2,44E-02	ENSG00000237667
SPDYE2	-6,13	-0,14	2,44E-02	ENSG00000205238
PARAL1	-6,32	0,00	2,45E-02	ENSG00000243961
RFTN1	2,04	5,10	2,46E-02	ENSG00000131378
SEMA5B	-2,91	6,30	2,46E-02	ENSG00000082684

AC106820.4	-3,49	3,06	2,47E-02	ENSG00000260293
SLAMF9	-5,75	2,29	2,47E-02	ENSG00000162723
AC015909.2	4,70	1,59	2,47E-02	ENSG00000253730
SLC52A1	-3,44	2,65	2,47E-02	ENSG00000132517
KCND3	3,15	2,43	2,47E-02	ENSG00000171385
SIX4	-2,75	3,71	2,48E-02	ENSG00000100625
C11orf96	2,22	5,67	2,49E-02	ENSG00000187479
FGF22	3,48	0,66	2,49E-02	ENSG00000070388
AC103564.2	-4,71	3,22	2,49E-02	ENSG00000232760
TGFB2-AS1	-3,57	0,25	2,50E-02	ENSG00000232480
RPL13AP7	-3,57	0,25	2,50E-02	ENSG00000213885
HOXC10	-2,23	5,98	2,50E-02	ENSG00000180818
ATXN7L1	-2,52	5,12	2,51E-02	ENSG00000146776
TREM2	3,14	3,92	2,51E-02	ENSG00000095970
FEV	3,89	0,54	2,52E-02	ENSG00000163497
PLIN3	2,12	6,63	2,52E-02	ENSG00000105355
CACNB1	-2,09	5,49	2,52E-02	ENSG00000067191
AC092490.1	6,47	-0,20	2,52E-02	ENSG00000249790
CSAG1	-5,40	3,17	2,52E-02	ENSG00000198930
CMYA5	2,42	5,81	2,52E-02	ENSG00000164309
COG7	-1,96	6,29	2,52E-02	ENSG00000168434
SCO2	4,30	0,17	2,53E-02	ENSG00000130489
AC025884.1	-3,96	0,53	2,53E-02	ENSG00000258732
WNK2	-2,87	4,23	2,53E-02	ENSG00000165238
LRRIQ1	5,99	-0,41	2,55E-02	ENSG00000133640
PTPRF	-2,31	7,56	2,56E-02	ENSG00000142949
CABP1	2,91	2,45	2,56E-02	ENSG00000157782
GSTM3P1	-3,88	0,47	2,56E-02	ENSG00000227693
PTCH1	2,40	5,88	2,56E-02	ENSG00000185920
GJA3	-4,61	3,37	2,57E-02	ENSG00000121743
SCUBE3	2,69	4,06	2,57E-02	ENSG00000146197
DNAH12	3,33	4,32	2,57E-02	ENSG00000174844
COL5A3	2,05	6,82	2,57E-02	ENSG00000080573
GEM	2,06	6,48	2,58E-02	ENSG00000164949
TNNC1	3,22	2,33	2,58E-02	ENSG00000114854
SSC5D	-2,25	5,31	2,59E-02	ENSG00000179954
NPIPP1	-2,04	5,32	2,60E-02	ENSG00000188599
OGFRL1	2,14	5,37	2,60E-02	ENSG00000119900
DPY19L2	-2,43	6,97	2,61E-02	ENSG00000177990
NKILA	2,94	2,33	2,61E-02	ENSG00000278709
ZDHHC2	-2,14	5,20	2,61E-02	ENSG00000104219
GNG7	2,71	3,61	2,61E-02	ENSG00000176533
CALCRL	2,19	4,97	2,61E-02	ENSG00000064989
AC106786.1	-4,96	1,32	2,62E-02	ENSG00000223652
PGM5P4	3,61	0,20	2,62E-02	ENSG00000225398
SLC10A4	-3,74	5,22	2,62E-02	ENSG00000145248
CASP1	2,50	3,83	2,63E-02	ENSG00000137752
ZFPM2	-2,92	5,24	2,63E-02	ENSG00000169946
ITIH6	-4,83	3,38	2,63E-02	ENSG00000102313
DRD1	-4,22	3,40	2,63E-02	ENSG00000184845
FAM157A	-3,25	1,25	2,63E-02	ENSG00000236438
SDCBP2-AS1	-2,67	2,92	2,63E-02	ENSG00000234684
B3GALT1	-3,64	3,52	2,64E-02	ENSG00000172318
AC116407.2	-3,75	3,58	2,66E-02	ENSG00000277511
CADM4	2,55	5,34	2,66E-02	ENSG00000105767
SLC11A1	2,50	4,32	2,66E-02	ENSG00000018280
CPSF1P1	-4,43	0,28	2,67E-02	ENSG00000214076
AC073133.2	-3,65	0,30	2,67E-02	ENSG00000233878
GPSM1	-2,11	7,47	2,68E-02	ENSG00000160360
LRP10	2,21	8,14	2,68E-02	ENSG00000197324
OSGIN2	2,61	4,08	2,68E-02	ENSG00000164823
CDCA7	-3,51	4,81	2,69E-02	ENSG00000144354
PAQR5	3,15	2,16	2,69E-02	ENSG00000137819
PCBP3	2,99	2,85	2,69E-02	ENSG00000183570

KLK1	-4,38	4,58	2,69E-02	ENSG00000167748
AGAP2	2,28	4,78	2,69E-02	ENSG00000135439
CLCNKA	-3,38	1,66	2,69E-02	ENSG00000186510
GOLGA2P10	-2,45	3,76	2,69E-02	ENSG00000255769
FAM41C	-4,94	1,68	2,69E-02	ENSG00000230368
MSRB3	2,03	7,42	2,70E-02	ENSG00000174099
PLEK2	3,20	1,03	2,70E-02	ENSG00000100558
STK26	-3,12	5,49	2,71E-02	ENSG00000134602
AC091059.1	2,88	2,21	2,71E-02	ENSG00000266002
AC108865.2	-4,86	0,59	2,71E-02	ENSG00000272218
DACH1	-3,40	4,17	2,72E-02	ENSG00000276644
LRP5	-2,02	5,46	2,72E-02	ENSG00000162337
PERM1	-2,67	3,09	2,72E-02	ENSG00000187642
TNFRSF8	3,25	1,94	2,73E-02	ENSG00000120949
ZNF503	-2,56	5,88	2,73E-02	ENSG00000165655
HOXA10	-2,33	4,80	2,74E-02	ENSG00000253293
FCGR2C	3,49	4,16	2,74E-02	ENSG00000244682
MYBPHL	4,22	0,11	2,74E-02	ENSG00000221986
LINC01060	4,22	0,11	2,74E-02	ENSG00000249378
C16orf82	4,22	0,11	2,75E-02	ENSG00000234186
GPR143	-3,90	2,42	2,75E-02	ENSG00000101850
DDR1	2,05	6,76	2,75E-02	ENSG00000204580
AL162591.2	3,21	1,83	2,75E-02	ENSG00000273110
HCG22	3,86	0,51	2,75E-02	ENSG00000228789
SNHG18	-2,56	4,65	2,75E-02	ENSG00000250786
CACHD1	-2,21	4,93	2,76E-02	ENSG00000158966
SLC5A3	2,31	6,99	2,77E-02	ENSG00000198743
AC105446.1	3,33	1,39	2,78E-02	ENSG00000227053
CES5AP1	4,55	1,86	2,78E-02	ENSG00000215478
SYNDIG1	-3,04	3,64	2,78E-02	ENSG00000101463
AP003396.5	1,91	5,71	2,78E-02	ENSG00000263873
EDN2	-3,41	0,77	2,78E-02	ENSG00000127129
AC009542.2	-5,48	1,93	2,78E-02	ENSG00000276067
SH3D21	-2,08	6,32	2,78E-02	ENSG00000214193
PECAM1	2,08	7,77	2,79E-02	ENSG00000261371
FAM155B	4,14	0,61	2,79E-02	ENSG00000130054
MT-TY	2,87	5,72	2,79E-02	ENSG00000210144
GGTA1P	2,18	4,82	2,79E-02	ENSG00000204136
AL592146.1	-2,88	4,85	2,80E-02	ENSG00000261065
GPC6	-2,23	4,58	2,80E-02	ENSG00000183098
IL18R1	-2,89	3,82	2,80E-02	ENSG00000115604
FTH1P7	3,23	0,76	2,80E-02	ENSG00000232187
AC018638.5	-1,99	5,81	2,81E-02	ENSG00000243679
AC023794.3	3,30	1,42	2,82E-02	ENSG00000250654
AL359881.2	-4,28	0,16	2,82E-02	ENSG00000270035
MYLK3	3,18	1,05	2,83E-02	ENSG00000140795
IL6R	2,63	4,75	2,83E-02	ENSG00000160712
ARHGAP15	2,49	3,40	2,83E-02	ENSG00000075884
SYTL5	-4,35	1,73	2,83E-02	ENSG00000147041
CDHR3	-2,72	4,64	2,84E-02	ENSG00000128536
ARHGAP6	-2,23	5,64	2,84E-02	ENSG00000047648
PTP4A2P2	3,98	1,15	2,84E-02	ENSG00000254481
CASC8	-4,14	0,63	2,84E-02	ENSG00000246228
FTLP3	2,93	2,10	2,85E-02	ENSG00000226608
AC112198.2	5,77	-0,53	2,85E-02	ENSG00000232517
GREB1	2,96	4,63	2,86E-02	ENSG00000196208
THSD4	-1,94	5,55	2,86E-02	ENSG00000187720
AL035252.3	3,45	1,88	2,86E-02	ENSG00000277938
WNT10B	3,23	4,39	2,87E-02	ENSG00000169884
TRAF1	2,20	7,12	2,87E-02	ENSG00000056558
CYFIP2	2,11	4,92	2,87E-02	ENSG00000055163
GLIS1	-3,18	2,82	2,87E-02	ENSG00000174332
AC245884.1	4,65	0,89	2,88E-02	ENSG00000227407
LRRC8B	2,73	6,86	2,88E-02	ENSG00000197147

FOXF1	-2,56	4,49	2,89E-02	ENSG00000103241
AC080038.3	-2,92	3,97	2,90E-02	ENSG00000279713
SOCS3	2,12	7,30	2,90E-02	ENSG00000184557
AC004080.1	-5,28	1,72	2,90E-02	ENSG00000253308
AC079684.1	-3,45	0,54	2,90E-02	ENSG00000274943
MALAT1	-2,18	10,30	2,91E-02	ENSG00000251562
AC091588.3	-2,74	3,20	2,91E-02	ENSG00000266283
AC092354.2	-4,12	2,30	2,91E-02	ENSG00000272370
DPH3	2,06	4,82	2,92E-02	ENSG00000154813
RSPO4	-3,36	2,35	2,92E-02	ENSG00000101282
MAN1C1	2,14	4,75	2,92E-02	ENSG00000117643
AC241377.3	3,96	0,68	2,94E-02	ENSG00000281741
OR51E2	3,83	0,53	2,94E-02	ENSG00000167332
AL117190.2	-2,23	7,36	2,94E-02	ENSG00000258663
GRIK3	2,97	3,74	2,95E-02	ENSG00000163873
PENK	-4,27	4,34	2,95E-02	ENSG00000181195
AC009495.3	-3,49	0,19	2,95E-02	ENSG00000235192
TUBE1	-2,04	5,93	2,96E-02	ENSG00000074935
SLX4	-2,03	4,93	2,96E-02	ENSG00000188827
ITGBL1	2,59	4,18	2,97E-02	ENSG00000198542
RPS6KA6	-2,52	3,26	2,97E-02	ENSG00000072133
DUXAP8	-2,89	3,44	2,97E-02	ENSG00000206195
RSU1	2,21	6,98	2,97E-02	ENSG00000148484
PLSCR3	-5,77	-0,39	2,97E-02	ENSG00000187838
HEYL	2,06	4,88	2,99E-02	ENSG00000163909
AP000696.1	-4,98	1,37	2,99E-02	ENSG00000231324
CCNI2	2,69	3,37	3,00E-02	ENSG00000205089
DHRS13	-2,43	3,97	3,01E-02	ENSG00000167536
TGFBR2	1,91	6,76	3,01E-02	ENSG00000163513
BCL6B	2,23	4,54	3,01E-02	ENSG00000161940
PCDHB11	-3,04	2,72	3,01E-02	ENSG00000197479
LHX9	-3,20	3,90	3,01E-02	ENSG00000143355
KIAA1324L	-2,54	5,13	3,02E-02	ENSG00000164659
FOXL2	-5,33	1,79	3,02E-02	ENSG00000183770
MAPK8IP1	2,22	5,63	3,02E-02	ENSG00000121653
TRAPPC3L	3,15	0,77	3,03E-02	ENSG00000173626
IGHV3-73	-5,54	2,10	3,03E-02	ENSG00000211976
AC092384.2	-3,20	1,33	3,03E-02	ENSG00000259881
AP000356.3	-3,35	1,89	3,04E-02	ENSG00000284128
TTC28-AS1	-2,07	5,83	3,04E-02	ENSG00000235954
PLOD2	-2,42	7,22	3,04E-02	ENSG00000152952
PVT1	-2,08	4,75	3,04E-02	ENSG00000249859
RAB40B	2,19	6,02	3,04E-02	ENSG00000141542
PMEPA1	2,05	7,68	3,04E-02	ENSG00000124225
BTG2	1,96	6,79	3,04E-02	ENSG00000159388
UCP1	4,39	0,31	3,04E-02	ENSG00000109424
HOXA13	-4,08	4,46	3,05E-02	ENSG00000106031
AC093724.1	-3,81	2,04	3,05E-02	ENSG00000213222
TST	2,37	4,08	3,05E-02	ENSG00000128311
FILIP1	2,50	3,46	3,05E-02	ENSG00000118407
ANXA9	2,82	2,82	3,06E-02	ENSG00000143412
VPS13B	-1,94	6,35	3,06E-02	ENSG00000132549
CCNO	-3,07	2,84	3,07E-02	ENSG00000152669
RAC2	2,25	4,30	3,07E-02	ENSG00000128340
EGFR-AS1	-4,24	0,14	3,07E-02	ENSG00000224057
FKBP1C	4,21	0,04	3,08E-02	ENSG00000198225
AC020909.2	3,09	2,28	3,08E-02	ENSG00000268518
WDR91	-2,00	6,67	3,09E-02	ENSG00000105875
CR381670.1	-3,87	2,65	3,09E-02	ENSG00000278931
CHD7	2,14	4,75	3,10E-02	ENSG00000171316
AC007620.2	2,36	3,81	3,10E-02	ENSG00000242539
GTF2IP20	-2,07	5,05	3,10E-02	ENSG00000272645
MDFI	-2,14	6,05	3,10E-02	ENSG00000112559
L3MBTL1	-2,16	5,18	3,11E-02	ENSG00000185513

IL1RL2	-3,06	1,75	3,11E-02	ENSG00000115598
HERC2	-2,33	8,19	3,11E-02	ENSG00000128731
AL591428.1	-5,71	-0,43	3,11E-02	ENSG00000285652
AC060780.1	-2,03	5,13	3,12E-02	ENSG00000267002
ITGA9	-2,03	5,79	3,13E-02	ENSG00000144668
HCN3	-2,36	3,82	3,13E-02	ENSG00000143630
PLPP2	-2,64	6,23	3,14E-02	ENSG00000141934
ADAM12	-2,04	4,84	3,14E-02	ENSG00000148848
MMP11	-2,08	7,00	3,14E-02	ENSG00000099953
PCA3	2,85	3,08	3,15E-02	ENSG00000225937
SLC25A27	-2,91	6,64	3,15E-02	ENSG00000153291
EML4	-2,12	7,42	3,15E-02	ENSG00000143924
SRCIN1	-2,53	5,01	3,16E-02	ENSG00000277363
AC005703.6	2,59	3,81	3,16E-02	ENSG00000279660
NALCN	3,64	1,79	3,17E-02	ENSG00000102452
FRRS1L	-3,16	2,09	3,17E-02	ENSG00000260230
TYRO3	2,34	4,86	3,17E-02	ENSG00000092445
AL021026.1	5,78	-0,49	3,18E-02	ENSG00000225243
IL34	2,45	5,25	3,18E-02	ENSG00000157368
HECW1	-4,19	1,80	3,18E-02	ENSG00000002746
P3H4	-1,96	6,52	3,18E-02	ENSG00000141696
ADH6	-3,08	2,17	3,18E-02	ENSG00000172955
TRNP1	2,33	4,43	3,18E-02	ENSG00000253368
HYMAI	-3,21	1,53	3,19E-02	ENSG00000283122
CBX7	1,89	5,77	3,19E-02	ENSG00000100307
LFNG	2,25	4,77	3,19E-02	ENSG00000106003
CYP4F11	-5,48	2,04	3,19E-02	ENSG00000171903
AL645608.7	3,23	1,83	3,19E-02	ENSG00000272512
HRCT1	2,85	2,58	3,19E-02	ENSG00000196196
MTCO1P12	2,58	6,83	3,20E-02	ENSG00000237973
ACTR3B	2,42	5,72	3,20E-02	ENSG00000133627
C6orf223	-5,81	-0,36	3,20E-02	ENSG00000181577
MID1IP1	2,17	5,39	3,20E-02	ENSG00000165175
PACSIN3	2,29	5,98	3,21E-02	ENSG00000165912
AC090510.3	-2,56	4,50	3,21E-02	ENSG00000278769
SIX2	3,05	2,10	3,21E-02	ENSG00000170577
PRTG	-2,84	3,43	3,22E-02	ENSG00000166450
LRRC2	3,09	0,92	3,22E-02	ENSG00000163827
PANX3	-5,95	-0,26	3,22E-02	ENSG00000154143
Z98259.1	-5,95	-0,26	3,23E-02	ENSG00000260972
TPTE2P2	-5,95	-0,26	3,23E-02	ENSG00000272281
CPLANE1	-1,87	5,52	3,23E-02	ENSG00000197603
LCP1	1,92	5,16	3,23E-02	ENSG00000136167
AC087521.1	2,36	4,78	3,24E-02	ENSG00000244953
EGR2	2,72	6,00	3,24E-02	ENSG00000122877
ARFGEF2	-2,19	6,87	3,25E-02	ENSG00000124198
GMFG	2,24	4,81	3,26E-02	ENSG00000130755
MT1F	2,53	4,43	3,26E-02	ENSG00000198417
UBXN10	2,78	2,03	3,27E-02	ENSG00000162543
KDM5B	-1,99	7,06	3,27E-02	ENSG00000117139
LINC01105	-5,11	1,64	3,27E-02	ENSG00000232044
CSRNP3	-2,23	5,06	3,28E-02	ENSG00000178662
EIF5	1,88	6,85	3,28E-02	ENSG00000100664
NRN1	2,38	4,58	3,29E-02	ENSG00000124785
MDGA2	-4,88	4,21	3,29E-02	ENSG00000139915
IFI27L2	2,30	5,62	3,30E-02	ENSG00000119632
AC079089.1	-4,72	1,51	3,31E-02	ENSG00000246528
AL445649.1	-3,89	1,35	3,31E-02	ENSG00000276740
DAB2	1,96	6,81	3,31E-02	ENSG00000153071
NUP210	2,22	4,27	3,31E-02	ENSG00000132182
TMEM178B	3,38	4,34	3,31E-02	ENSG00000261115
GPSM3	2,30	5,09	3,32E-02	ENSG00000213654
NLRP3	2,82	2,76	3,33E-02	ENSG00000162711
C11orf87	-3,86	1,77	3,33E-02	ENSG00000185742

SLC17A7	2,80	2,12	3,34E-02	ENSG00000104888
USP32P1	-3,19	2,35	3,35E-02	ENSG00000188933
AC005339.1	2,41	3,53	3,35E-02	ENSG00000268565
PIM1	2,07	4,70	3,36E-02	ENSG00000137193
AL512662.2	-4,32	1,37	3,36E-02	ENSG00000283913
BOC	-2,20	7,92	3,36E-02	ENSG00000144857
KLHL2	1,99	5,26	3,36E-02	ENSG00000109466
AL021707.5	2,22	4,61	3,36E-02	ENSG00000244491
HES4	2,20	5,55	3,36E-02	ENSG00000188290
FOXI2	-5,05	4,33	3,38E-02	ENSG00000186766
LPIN3	-2,18	6,97	3,38E-02	ENSG00000132793
AC026369.1	-3,99	0,91	3,38E-02	ENSG00000249695
AL031595.1	2,53	3,42	3,38E-02	ENSG00000279933
SYNM	2,28	6,90	3,39E-02	ENSG00000182253
LINC00622	2,99	1,48	3,39E-02	ENSG00000260941
GAL	3,29	0,93	3,40E-02	ENSG00000069482
TMEM63C	3,27	1,96	3,40E-02	ENSG00000165548
IFI44L	2,09	5,25	3,40E-02	ENSG00000137959
ALB	-3,16	1,34	3,40E-02	ENSG00000163631
ZNF208	-2,89	2,80	3,40E-02	ENSG00000160321
HYAL1	-2,27	4,67	3,41E-02	ENSG00000114378
AL031600.1	-3,53	1,66	3,41E-02	ENSG00000260051
UVSSA	-1,89	6,39	3,41E-02	ENSG00000163945
AF181450.1	-3,73	0,35	3,41E-02	ENSG00000253576
GDF15	2,09	5,11	3,41E-02	ENSG00000130513
PLCB2	2,41	6,15	3,42E-02	ENSG00000137841
AP001636.3	3,86	1,06	3,42E-02	ENSG00000255240
AC004765.1	-5,83	-0,36	3,43E-02	ENSG00000285704
SLC7A7	2,42	3,72	3,43E-02	ENSG00000155465
RARRES3	2,17	4,28	3,43E-02	ENSG00000133321
ANKRD29	2,37	4,24	3,43E-02	ENSG00000154065
CSTA	2,90	2,46	3,44E-02	ENSG00000121552
SYNGR3	-3,65	2,47	3,44E-02	ENSG00000127561
SH3BP5	1,99	6,71	3,44E-02	ENSG00000131370
DLEU7	3,58	0,29	3,44E-02	ENSG00000186047
LINC02458	3,44	0,66	3,44E-02	ENSG00000246363
AC004585.1	5,77	-0,57	3,45E-02	ENSG00000266088
AC008780.2	5,77	-0,57	3,45E-02	ENSG00000249436
CERKL	-2,70	5,46	3,45E-02	ENSG00000188452
RASAL1	4,20	2,74	3,45E-02	ENSG00000111344
AC016026.1	-5,68	-0,45	3,45E-02	ENSG00000093100
AL355377.2	4,08	-0,06	3,45E-02	ENSG00000279489
PCDHB5	-2,68	2,61	3,46E-02	ENSG00000113209
HIST2H2BD	-2,25	3,94	3,46E-02	ENSG00000220323
HIST1H2BJ	3,12	0,73	3,46E-02	ENSG00000124635
UBASH3A	2,82	1,31	3,48E-02	ENSG00000160185
PLPPR4	2,53	3,98	3,48E-02	ENSG00000117600
AC092375.2	3,21	0,95	3,49E-02	ENSG00000260306
BAIAP2L1	-2,31	5,24	3,49E-02	ENSG00000006453
N4BP3	2,81	1,84	3,49E-02	ENSG00000145911
LINC00920	4,12	-0,03	3,49E-02	ENSG00000246898
AL031666.1	4,12	-0,03	3,50E-02	ENSG00000231119
TRIML2	-3,99	4,08	3,50E-02	ENSG00000179046
AGGF1P2	4,38	0,22	3,51E-02	ENSG00000233435
CCDC43	2,08	5,39	3,51E-02	ENSG00000180329
GZMK	2,74	2,34	3,52E-02	ENSG00000113088
ST6GAL2	2,67	5,15	3,52E-02	ENSG00000144057
AC243962.1	4,26	0,20	3,52E-02	ENSG00000225370
ITLN1	4,89	2,57	3,52E-02	ENSG00000179914
CCR5	2,57	2,54	3,52E-02	ENSG00000160791
NYAP1	-4,07	3,65	3,53E-02	ENSG00000166924
ACAN	-2,78	6,68	3,53E-02	ENSG00000157766
FAM84B	-2,62	5,90	3,53E-02	ENSG00000168672
NUP210L	-3,38	0,11	3,53E-02	ENSG00000143552

ZIC2	-5,04	1,58	3,54E-02	ENSG00000043355
MDGA1	2,25	6,75	3,54E-02	ENSG00000112139
MPP3	2,91	5,81	3,54E-02	ENSG00000161647
AC015922.3	2,55	2,83	3,54E-02	ENSG00000276855
GOLGA8A	-2,07	8,97	3,55E-02	ENSG00000175265
NPR1	2,52	4,03	3,55E-02	ENSG00000169418
AC243829.4	2,46	4,38	3,55E-02	ENSG00000277089
PTGER1	-3,89	1,35	3,55E-02	ENSG00000160951
AC026471.3	-3,54	0,60	3,56E-02	ENSG00000260740
AC007906.2	3,02	1,81	3,56E-02	ENSG00000277639
TRAF3IP3	2,46	3,58	3,56E-02	ENSG00000009790
LINC01736	4,29	0,23	3,56E-02	ENSG00000228058
TMEM100	2,73	2,37	3,56E-02	ENSG00000166292
AL138963.3	1,97	9,89	3,57E-02	ENSG00000273149
AL354811.1	-3,55	0,22	3,57E-02	ENSG00000278177
FOXL1	2,70	2,82	3,57E-02	ENSG00000176678
OR2W3	-5,81	-0,37	3,57E-02	ENSG00000238243
POPDC3	3,33	4,16	3,57E-02	ENSG00000132429
KCNC3	2,80	2,91	3,57E-02	ENSG00000131398
CNTNAP1	-2,20	6,04	3,58E-02	ENSG00000108797
SLC4A4	3,26	1,73	3,58E-02	ENSG00000080493
C10orf55	2,53	3,31	3,59E-02	ENSG00000222047
AC016597.1	-5,79	-0,37	3,59E-02	ENSG00000261329
GOLGA7B	2,70	2,58	3,60E-02	ENSG00000155265
INKA2-AS1	2,96	1,25	3,61E-02	ENSG00000227811
FRMD1	4,86	2,80	3,62E-02	ENSG00000153303
SIX1	-2,72	4,64	3,62E-02	ENSG00000126778
RERG	2,09	4,73	3,63E-02	ENSG00000134533
KLHDC9	-2,61	2,66	3,63E-02	ENSG00000162755
ST6GALNAC5	3,03	3,90	3,63E-02	ENSG00000117069
LRGUK	-3,00	2,95	3,63E-02	ENSG00000155530
FAM9B	-4,15	0,07	3,63E-02	ENSG00000177138
STK39	-2,15	4,99	3,63E-02	ENSG00000198648
GOLM1	-1,85	7,34	3,64E-02	ENSG00000135052
CIPC	2,32	4,12	3,64E-02	ENSG00000198894
DCAF12L2	-3,85	1,10	3,64E-02	ENSG00000198354
ZG16B	3,49	3,45	3,65E-02	ENSG00000162078
GPR34	2,42	3,74	3,65E-02	ENSG00000171659
GPR83	5,61	-0,64	3,65E-02	ENSG00000123901
ARL4D	2,56	3,65	3,65E-02	ENSG00000175906
ROR2	-1,81	5,69	3,65E-02	ENSG00000169071
ASTN2	2,20	4,51	3,66E-02	ENSG00000148219
SLC35E2A	-1,92	5,55	3,66E-02	ENSG00000215790
ADCY1	2,61	2,52	3,67E-02	ENSG00000164742
ALDH1A2	-3,74	4,82	3,67E-02	ENSG00000128918
Z84488.1	2,75	2,13	3,67E-02	ENSG00000244158
OLFM4	4,10	-0,02	3,67E-02	ENSG00000102837
ALDH1A1	2,25	3,82	3,68E-02	ENSG00000165092
AL390728.3	-3,41	0,13	3,68E-02	ENSG00000224014
AC099548.2	-3,41	0,13	3,68E-02	ENSG00000253882
METRN	1,96	5,30	3,68E-02	ENSG00000103260
MAGEA4	-5,86	-0,32	3,68E-02	ENSG00000147381
SUN2	2,04	7,79	3,69E-02	ENSG00000100242
SCD5	2,06	5,93	3,69E-02	ENSG00000145284
MYL12A	1,86	7,85	3,70E-02	ENSG00000101608
CCR2	2,89	1,97	3,70E-02	ENSG00000121807
FRMD8	1,96	6,38	3,70E-02	ENSG00000126391
QPCT	2,55	2,58	3,70E-02	ENSG00000115828
TRIM58	-3,68	0,32	3,71E-02	ENSG00000162722
SLC28A3	3,94	1,30	3,71E-02	ENSG00000197506
RGS5	1,84	7,10	3,72E-02	ENSG00000143248
TPM1	1,89	8,68	3,73E-02	ENSG00000140416
AC068385.1	2,25	4,14	3,73E-02	ENSG00000255498
ZNF608	2,09	4,77	3,74E-02	ENSG00000168916

GINS3	2,53	3,57	3,74E-02	ENSG00000181938
AL359091.4	-2,90	1,10	3,74E-02	ENSG00000272696
WARS	1,95	7,22	3,75E-02	ENSG00000140105
AC008676.3	2,82	2,38	3,75E-02	ENSG00000285868
SLC38A8	4,03	-0,01	3,75E-02	ENSG00000166558
AC011495.3	3,52	1,17	3,76E-02	ENSG00000268677
SYT5	3,08	1,64	3,76E-02	ENSG00000129990
ZSWIM5	-2,38	4,20	3,77E-02	ENSG00000162415
MYL1	5,63	-0,59	3,77E-02	ENSG00000168530
ZNF99	-3,52	0,21	3,78E-02	ENSG00000213973
PMEL	-2,16	4,44	3,78E-02	ENSG00000185664
AC005005.3	2,50	3,07	3,78E-02	ENSG00000273387
MAGEH1	-2,43	6,63	3,79E-02	ENSG00000187601
SLC6A17	3,21	1,32	3,79E-02	ENSG00000197106
LUARIS	-4,05	1,12	3,80E-02	ENSG00000231638
AP005131.1	-4,14	0,07	3,80E-02	ENSG00000267136
LINC02102	-4,14	0,07	3,80E-02	ENSG00000248677
MIR210HG	-2,33	4,22	3,80E-02	ENSG00000247095
MFSD13B	-3,50	0,18	3,81E-02	ENSG00000230872
CSPG4P10	-2,56	3,23	3,81E-02	ENSG00000276710
MIOX	4,65	3,52	3,81E-02	ENSG00000100253
DUSP5	1,88	5,13	3,81E-02	ENSG00000138166
ELOVL5	1,99	6,57	3,82E-02	ENSG00000012660
AC097493.4	4,19	0,15	3,82E-02	ENSG00000284648
TCF15	2,79	1,71	3,82E-02	ENSG00000125878
KCNG2	3,06	2,08	3,82E-02	ENSG00000178342
SIRPB2	2,91	2,03	3,82E-02	ENSG00000196209
ASCL2	3,19	1,54	3,82E-02	ENSG00000183734
BX119927.1	6,17	-0,43	3,83E-02	ENSG00000283178
USHBP1	2,71	3,88	3,84E-02	ENSG00000130307
AL591845.1	-2,24	4,10	3,84E-02	ENSG00000116883
NSA2	1,85	6,10	3,85E-02	ENSG00000164346
TMEM132B	2,79	2,83	3,85E-02	ENSG00000139364
SELP	3,39	3,97	3,85E-02	ENSG00000174175
AC241644.1	5,71	-0,54	3,86E-02	ENSG00000227139
LINC00706	5,71	-0,54	3,86E-02	ENSG00000281186
MIR5689HG	5,71	-0,54	3,87E-02	ENSG00000229401
CDH5	1,96	6,40	3,87E-02	ENSG00000179776
MGAT5B	-3,10	2,66	3,87E-02	ENSG00000167889
PLPBP	2,00	4,65	3,87E-02	ENSG00000147471
EMILIN1	-2,45	7,70	3,88E-02	ENSG00000138080
PCOLCE-AS1	-2,86	6,73	3,88E-02	ENSG00000224729
NRL	2,89	1,37	3,89E-02	ENSG00000129535
BIRC6-AS2	-5,66	-0,46	3,90E-02	ENSG00000279897
ARRB1	2,15	4,17	3,90E-02	ENSG00000137486
FSIP2-AS1	-3,26	1,26	3,91E-02	ENSG00000231646
LINC01945	-4,22	1,38	3,91E-02	ENSG00000236485
HOXC11	-2,41	3,18	3,91E-02	ENSG00000123388
ANOS1	-2,36	5,12	3,92E-02	ENSG00000011201
SLC9A7	2,31	5,16	3,92E-02	ENSG00000065923
MIR1184-1	-3,16	1,34	3,93E-02	ENSG00000221533
LINC01778	3,70	1,15	3,93E-02	ENSG00000223382
C12orf56	-3,98	3,27	3,93E-02	ENSG00000185306
DLX6-AS1	-3,69	3,37	3,93E-02	ENSG00000231764
TFF3	4,16	0,00	3,93E-02	ENSG00000160180
TREML1	4,36	1,56	3,93E-02	ENSG00000161911
TFDP1	1,88	5,58	3,94E-02	ENSG00000198176
ADGRD1	2,31	4,79	3,94E-02	ENSG00000111452
UGT2B7	4,23	1,08	3,94E-02	ENSG00000171234
FASLG	3,15	0,41	3,95E-02	ENSG00000117560
CDH4	-2,28	6,22	3,95E-02	ENSG00000179242
RPL3	1,91	10,12	3,95E-02	ENSG00000100316
AL590369.1	-2,74	1,51	3,96E-02	ENSG00000234789
BCORL1	-1,91	5,16	3,97E-02	ENSG00000085185

ITIH2	2,97	1,34	3,97E-02	ENSG00000151655
TMEM273	2,38	4,54	3,97E-02	ENSG00000204161
ELOVL4	2,76	2,45	3,98E-02	ENSG00000118402
EFNA3	-2,77	3,17	3,98E-02	ENSG00000143590
PPP1R1B	3,48	1,75	3,98E-02	ENSG00000131771
LUM	1,84	8,21	3,99E-02	ENSG00000139329
CR381653.2	-2,26	3,78	3,99E-02	ENSG00000279208
MFNG	2,29	3,89	4,00E-02	ENSG00000100060
SLC16A4	2,46	3,65	4,00E-02	ENSG00000168679
FAAH	-2,02	4,82	4,00E-02	ENSG00000117480
PLAU	1,99	5,45	4,00E-02	ENSG00000122861
CASK	-1,87	6,10	4,00E-02	ENSG00000147044
WDR19	-1,83	5,63	4,00E-02	ENSG00000157796
SLC45A3	-2,30	4,34	4,01E-02	ENSG00000158715
IGSF21	-3,07	4,88	4,01E-02	ENSG00000117154
ANKRD36	-2,13	4,19	4,03E-02	ENSG00000135976
HLA-DPB1	2,20	7,89	4,03E-02	ENSG00000223865
HEY1	2,14	4,99	4,04E-02	ENSG00000164683
ABHD11-AS1	2,57	4,37	4,05E-02	ENSG00000225969
AC245140.2	1,94	7,15	4,05E-02	ENSG00000280195
BOK-AS1	2,74	1,05	4,05E-02	ENSG00000234235
DUSP1	1,85	8,68	4,06E-02	ENSG00000120129
IGSF8	-2,38	6,59	4,06E-02	ENSG00000162729
AC211476.2	-5,55	-0,53	4,06E-02	ENSG00000272843
LINC00894	-2,28	3,69	4,07E-02	ENSG00000235703
CBR3	2,17	3,97	4,07E-02	ENSG00000159231
AC009495.1	-4,02	-0,02	4,07E-02	ENSG00000229195
DNM1	-2,03	7,54	4,07E-02	ENSG00000106976
STAG3L5P	-2,53	3,40	4,07E-02	ENSG00000242294
AL139241.1	2,87	1,95	4,07E-02	ENSG00000230928
GNB4	1,84	5,20	4,08E-02	ENSG00000114450
BEST4	-3,07	3,16	4,08E-02	ENSG00000142959
RORA	-2,22	6,44	4,08E-02	ENSG00000069667
MSTN	-2,89	2,83	4,09E-02	ENSG00000138379
TMEM116	-2,29	4,74	4,09E-02	ENSG00000198270
NR4A1	2,01	8,97	4,09E-02	ENSG00000123358
AC104083.1	2,23	3,89	4,09E-02	ENSG00000260244
DTX1	2,61	4,98	4,11E-02	ENSG00000135144
C22orf46	2,15	4,52	4,11E-02	ENSG00000184208
ARPIN	1,91	5,01	4,12E-02	ENSG00000242498
ENPP4	2,19	4,24	4,12E-02	ENSG00000001561
AMER2	-5,21	1,80	4,12E-02	ENSG00000165566
KATNAL2	-2,20	4,46	4,12E-02	ENSG00000167216
AL391832.2	-3,42	0,13	4,13E-02	ENSG00000238005
MTFP1	-3,68	0,29	4,13E-02	ENSG00000242114
KLK6	3,94	-0,08	4,13E-02	ENSG00000167755
RN7SL356P	3,94	-0,08	4,13E-02	ENSG00000266553
AC016738.1	-2,61	3,70	4,14E-02	ENSG00000223947
S100A14	4,64	0,26	4,14E-02	ENSG00000189334
PROS1	1,82	5,84	4,14E-02	ENSG00000184500
AC092171.5	-2,94	1,57	4,15E-02	ENSG00000273084
PRSS33	4,18	1,83	4,15E-02	ENSG00000103355
VAT1	1,99	7,44	4,16E-02	ENSG00000108828
GJB3	3,70	0,47	4,16E-02	ENSG00000188910
CAMK2D	1,92	6,32	4,16E-02	ENSG00000145349
KCNC1	3,92	3,17	4,17E-02	ENSG00000129159
NR1H3	1,88	5,36	4,17E-02	ENSG00000025434
ARRDC4	1,88	4,98	4,17E-02	ENSG00000140450
AL033379.1	-3,35	1,13	4,18E-02	ENSG00000224384
ANXA13	-3,83	1,76	4,18E-02	ENSG00000104537
GDAP1	2,62	2,83	4,18E-02	ENSG00000104381
EIF1AXP1	4,33	0,02	4,18E-02	ENSG00000236698
ZNF560	-5,58	-0,51	4,19E-02	ENSG00000198028
AGAP14P	-2,87	1,08	4,19E-02	ENSG00000279058

SLC13A4	-2,45	3,54	4,19E-02	ENSG00000164707
FABP7	4,73	1,69	4,20E-02	ENSG00000164434
LBX2-AS1	2,35	3,58	4,21E-02	ENSG00000257702
SKIV2L	-1,88	6,49	4,21E-02	ENSG00000204351
SDR42E1	-3,29	2,48	4,21E-02	ENSG00000184860
FAM228B	1,90	5,53	4,22E-02	ENSG00000219626
KRT19	4,04	2,89	4,22E-02	ENSG00000171345
PDLIM4	1,96	6,60	4,22E-02	ENSG00000131435
LNX1	-2,01	5,42	4,22E-02	ENSG00000072201
RASSF9	-2,43	3,96	4,23E-02	ENSG00000198774
CD53	2,16	4,92	4,24E-02	ENSG00000143119
MMD	2,23	4,94	4,24E-02	ENSG00000108960
AC008750.1	3,66	0,92	4,24E-02	ENSG00000254760
AC009065.2	-1,90	5,12	4,24E-02	ENSG00000259933
HOXC-AS3	-2,76	2,61	4,24E-02	ENSG00000251151
AL592430.2	-4,30	0,16	4,25E-02	ENSG00000282121
AC016582.1	-2,96	1,28	4,25E-02	ENSG00000225868
CHST9	-5,76	-0,39	4,25E-02	ENSG00000154080
AC010913.1	-3,50	0,55	4,26E-02	ENSG00000272702
AC055733.4	-5,17	1,77	4,26E-02	ENSG00000284095
TPSAB1	-2,65	2,22	4,27E-02	ENSG00000172236
KIF6	2,87	1,39	4,28E-02	ENSG00000164627
ACSS1	1,89	5,04	4,28E-02	ENSG00000154930
PRR5L	2,36	3,62	4,28E-02	ENSG00000135362
TMOD2	1,98	4,58	4,29E-02	ENSG00000128872
SIGLEC7	3,00	1,56	4,29E-02	ENSG00000168995
IGSF10	-2,42	4,29	4,29E-02	ENSG00000152580
AL669831.1	-2,48	2,43	4,29E-02	ENSG00000228327
LINC02202	3,09	1,08	4,29E-02	ENSG00000245812
CFAP44	-1,82	7,03	4,29E-02	ENSG00000206530
GLT8D2	-1,90	6,08	4,30E-02	ENSG00000120820
C1QTNF3	-2,62	4,90	4,30E-02	ENSG00000082196
OAS2	2,03	4,52	4,30E-02	ENSG00000111335
IGFBP7	1,80	9,13	4,31E-02	ENSG00000163453
EVI2A	2,49	3,21	4,31E-02	ENSG00000126860
LILRB4	2,50	3,83	4,31E-02	ENSG00000186818
APCDD1L	3,28	2,39	4,31E-02	ENSG00000198768
AC020663.3	-5,69	-0,44	4,31E-02	ENSG00000275056
WLS	-1,89	6,39	4,31E-02	ENSG00000116729
DIPK2B	1,92	5,52	4,32E-02	ENSG00000147113
ANO5	-2,36	4,47	4,33E-02	ENSG00000171714
AC008738.3	2,69	3,65	4,33E-02	ENSG00000267580
TIE1	2,37	7,64	4,34E-02	ENSG00000066056
ZNF436	1,82	5,44	4,35E-02	ENSG00000125945
AC008750.2	3,82	0,52	4,35E-02	ENSG00000255441
BIN1	2,00	6,14	4,35E-02	ENSG00000136717
PPP1R13L	-2,00	5,70	4,35E-02	ENSG00000104881
RAB11FIP5	-1,85	6,06	4,36E-02	ENSG00000135631
AJ239328.1	3,38	0,84	4,36E-02	ENSG00000280604
TPPP3	2,05	5,64	4,36E-02	ENSG00000159713
LINC02615	-2,28	3,74	4,37E-02	ENSG00000251432
AC091182.2	3,36	0,12	4,37E-02	ENSG00000253746
GPR141	-3,20	2,04	4,37E-02	ENSG00000187037
POFUT2	-1,75	7,22	4,39E-02	ENSG00000186866
AC016738.2	-3,25	0,39	4,40E-02	ENSG00000230140
FCMR	2,59	1,84	4,40E-02	ENSG00000162894
ENPP2	-1,87	5,98	4,40E-02	ENSG00000136960
MEI1	-2,50	3,09	4,41E-02	ENSG00000167077
NECTIN3	2,40	3,52	4,41E-02	ENSG00000177707
AL450326.1	3,18	-0,04	4,42E-02	ENSG00000230555
NR3C2	-2,55	4,28	4,42E-02	ENSG00000151623
GLDN	2,21	5,57	4,42E-02	ENSG00000186417
PGF	1,76	5,65	4,42E-02	ENSG00000119630
SCARNA7	-3,43	2,24	4,43E-02	ENSG00000238741

PPFIA2-AS1	-5,14	1,75	4,43E-02	ENSG00000257467
SEMA3C	2,78	6,14	4,45E-02	ENSG00000075223
CR392039.4	-3,79	0,78	4,46E-02	ENSG00000279720
GMFB	1,87	5,95	4,46E-02	ENSG00000197045
BX470102.2	2,92	1,49	4,46E-02	ENSG00000285867
AFDN	-1,88	7,44	4,46E-02	ENSG00000130396
AC090559.1	3,08	0,76	4,47E-02	ENSG00000255197
MFAP3L	2,45	3,05	4,47E-02	ENSG00000198948
KNDC1	3,52	6,91	4,48E-02	ENSG00000171798
CABIN1	-1,99	7,83	4,50E-02	ENSG00000099991
LINC02577	3,19	2,11	4,51E-02	ENSG00000228742
AP005329.1	1,88	6,48	4,51E-02	ENSG00000264235
GATA3	-2,62	4,27	4,51E-02	ENSG00000107485
GLT1D1	-3,39	2,01	4,52E-02	ENSG00000151948
TRPV4	2,35	4,83	4,53E-02	ENSG00000111199
BHMT	2,85	3,44	4,53E-02	ENSG00000145692
FRG1DP	-3,49	2,40	4,54E-02	ENSG00000282870
SLAMF7	2,41	2,79	4,54E-02	ENSG00000026751
PKP2	-3,05	5,19	4,54E-02	ENSG00000057294
CLIC4	1,86	8,48	4,54E-02	ENSG00000169504
NPAS2	-2,14	5,65	4,55E-02	ENSG00000170485
TFPI	1,87	4,92	4,55E-02	ENSG00000003436
ADAMTS19	-4,75	1,21	4,56E-02	ENSG00000145808
UNC80	3,30	1,77	4,56E-02	ENSG00000144406
CUL7	-1,76	6,78	4,58E-02	ENSG00000044090
AC099568.2	3,93	-0,14	4,58E-02	ENSG00000272931
RGS16	1,77	5,54	4,58E-02	ENSG00000143333
PNMA6A	-2,76	1,69	4,58E-02	ENSG00000235961
TTC17	-1,77	7,47	4,58E-02	ENSG00000052841
AP000688.2	4,03	0,03	4,58E-02	ENSG00000233393
TP53I11	1,87	5,14	4,58E-02	ENSG00000175274
PRKG2	-3,72	2,65	4,60E-02	ENSG00000138669
GOLGA7	1,76	6,11	4,61E-02	ENSG00000147533
SLC14A1	2,91	1,63	4,61E-02	ENSG00000141469
AC009237.15	-3,53	0,59	4,62E-02	ENSG00000273305
AMIGO2	2,54	2,65	4,63E-02	ENSG00000139211
GFOD1-AS1	3,91	-0,16	4,63E-02	ENSG00000237786
PHLDA1	1,83	7,36	4,63E-02	ENSG00000139289
EME2	-1,75	7,93	4,63E-02	ENSG00000197774
APBB1IP	2,45	3,62	4,64E-02	ENSG00000077420
AC005277.1	3,98	-0,14	4,64E-02	ENSG00000263707
CHGB	3,98	-0,14	4,64E-02	ENSG00000089199
APOBEC3G	2,20	4,03	4,64E-02	ENSG00000239713
AL157871.1	1,87	6,14	4,64E-02	ENSG00000258504
DOCK5	-1,96	7,20	4,64E-02	ENSG00000147459
E2F8	2,79	2,40	4,64E-02	ENSG00000129173
IGFBP2	1,75	5,66	4,65E-02	ENSG00000115457
AC083855.2	2,84	1,79	4,65E-02	ENSG00000285906
AC092364.2	-5,49	-0,56	4,65E-02	ENSG00000269845
SPATS2	-1,74	6,23	4,66E-02	ENSG00000123352
IGFBP4	1,74	8,50	4,66E-02	ENSG00000141753
MFSD6	2,05	5,45	4,66E-02	ENSG00000151690
IRF8	2,33	4,43	4,67E-02	ENSG00000140968
ITGA4	-2,87	5,29	4,67E-02	ENSG00000115232
GRIK5	2,59	3,10	4,67E-02	ENSG00000105737
FAM138E	5,46	-0,70	4,67E-02	ENSG00000248893
AP001107.5	2,73	1,06	4,67E-02	ENSG00000254510
RALGPS1	-2,07	4,09	4,68E-02	ENSG00000136828
LINC02398	-4,04	1,14	4,68E-02	ENSG00000256287
TMCC3	1,98	4,36	4,69E-02	ENSG00000057704
RFX2	-2,21	5,68	4,70E-02	ENSG00000087903
TDRD9	-3,00	3,65	4,70E-02	ENSG00000156414
LRSAM1	-1,74	5,91	4,71E-02	ENSG00000148356
UPP1	1,80	5,82	4,71E-02	ENSG00000183696

ST8SIA1	-3,29	3,71	4,71E-02	ENSG00000111728
SFTPA2	-4,40	0,24	4,71E-02	ENSG00000185303
SCAMP5	2,26	4,07	4,71E-02	ENSG00000198794
ACOT11	-2,38	3,46	4,72E-02	ENSG00000162390
PDHB	1,79	6,28	4,72E-02	ENSG00000168291
DNASE1	-1,74	7,29	4,72E-02	ENSG00000213918
AADAT	-2,24	4,67	4,73E-02	ENSG00000109576
CAMSAP2	-1,83	6,87	4,73E-02	ENSG00000118200
ENO2	-1,96	7,38	4,74E-02	ENSG00000111674
LINC00924	3,13	2,06	4,74E-02	ENSG00000259134
RFLNA	-2,84	4,85	4,74E-02	ENSG00000178882
HSD11B2	2,81	1,68	4,74E-02	ENSG00000176387
FBXW4P1	-2,47	3,37	4,74E-02	ENSG00000230701
CTBP2P8	-5,73	-0,42	4,75E-02	ENSG00000234383
ZNF727	-5,53	-0,54	4,75E-02	ENSG00000214652
AOC1	-5,73	-0,42	4,75E-02	ENSG00000002726
C3orf22	-5,53	-0,54	4,75E-02	ENSG00000180697
AL445183.2	-5,73	-0,42	4,75E-02	ENSG00000235563
CCDC106	1,94	4,98	4,75E-02	ENSG00000173581
SYT6	2,92	1,11	4,75E-02	ENSG00000134207
ZNF106	1,72	5,97	4,75E-02	ENSG00000103994
MIR6730	5,55	-0,64	4,75E-02	ENSG00000276830
ANKFN1	-4,73	2,56	4,76E-02	ENSG00000153930
MYPN	3,89	-0,11	4,77E-02	ENSG00000138347
EML2	1,97	4,76	4,77E-02	ENSG00000125746
ELFN1	-2,28	3,84	4,77E-02	ENSG00000225968
C3orf33	-2,65	1,83	4,77E-02	ENSG00000174928
MAN1A1	-2,35	6,98	4,78E-02	ENSG00000111885
AC009065.3	-3,14	-0,04	4,78E-02	ENSG00000260447
VSTM5	-2,67	1,37	4,78E-02	ENSG00000214376
LINC02210	-1,94	4,88	4,80E-02	ENSG00000204650
SRF	1,99	6,98	4,80E-02	ENSG00000112658
RPL5	1,78	9,14	4,80E-02	ENSG00000122406
MRC1	-2,44	4,36	4,80E-02	ENSG00000260314
ANOS2P	-5,06	1,68	4,80E-02	ENSG00000241859
NME7	-1,88	5,36	4,82E-02	ENSG00000143156
AC015921.1	3,56	0,31	4,82E-02	ENSG00000262884
AC004754.1	-2,11	3,89	4,82E-02	ENSG00000269881
LRP1B	-3,41	3,33	4,83E-02	ENSG00000168702
SCAT2	-2,74	1,49	4,83E-02	ENSG00000257596
SLC7A5	1,84	6,05	4,83E-02	ENSG00000103257
AC105206.2	3,18	-0,09	4,83E-02	ENSG00000254064
TMEM159	1,90	5,37	4,84E-02	ENSG00000011638
HMGN2P3	3,43	0,15	4,85E-02	ENSG00000230330
FRMD4A	1,96	7,22	4,85E-02	ENSG00000151474
MED15P8	-5,56	-0,52	4,85E-02	ENSG00000248796
ARRDC3-AS1	-3,13	-0,06	4,86E-02	ENSG00000281357
C11orf21	2,59	3,16	4,86E-02	ENSG00000110665
CCDC144CP	-2,69	2,36	4,86E-02	ENSG00000154898
CHI3L2	3,29	1,39	4,86E-02	ENSG00000064886
FLT3	3,26	1,38	4,86E-02	ENSG00000122025
MEIS3P1	2,61	1,12	4,86E-02	ENSG00000179277
TIAM2	-1,83	5,52	4,86E-02	ENSG00000146426
HTRA1	-1,74	8,13	4,87E-02	ENSG00000166033
OSGIN1	2,17	4,71	4,87E-02	ENSG00000140961
AC027237.3	2,94	0,84	4,88E-02	ENSG00000259426
PLK2	1,78	6,93	4,89E-02	ENSG00000145632
AL359697.1	-3,34	1,84	4,89E-02	ENSG00000279623
HDC	3,26	4,83	4,91E-02	ENSG00000140287
GUCY1B1	1,75	5,27	4,91E-02	ENSG00000061918
BIRC6-AS1	-5,41	-0,61	4,92E-02	ENSG00000230046
LINC02600	-3,79	3,87	4,93E-02	ENSG00000250986
SFTPD	-2,98	1,75	4,94E-02	ENSG00000133661
FAM124B	2,77	1,32	4,94E-02	ENSG00000124019

SNAPC4	-1,73	5,70	4,95E-02	ENSG00000165684
GPD1L	1,98	4,43	4,96E-02	ENSG00000152642
AL031846.2	2,19	3,48	4,96E-02	ENSG00000279833
WNT5B	-2,30	4,57	4,96E-02	ENSG00000111186
FBXO27	2,59	2,81	4,97E-02	ENSG00000161243
PCDH1	2,11	3,82	4,97E-02	ENSG00000156453
AC131025.2	3,08	-0,11	4,97E-02	ENSG00000275871
C6orf141	4,08	0,58	4,97E-02	ENSG00000197261
NPAS3	-2,65	4,10	4,97E-02	ENSG00000151322
RHOT2	-1,82	8,43	4,98E-02	ENSG00000140983
PBX4	-2,19	3,78	4,98E-02	ENSG00000105717
AC132938.5	-3,27	0,03	4,99E-02	ENSG00000279744
PCAT19	2,51	3,63	4,99E-02	ENSG00000267107
